# Multiscale Coupling From Mastication to Retronasal Aroma Perception: The PG‐DTCFN Model and Multiphysics Simulation

**DOI:** 10.1002/advs.76523

**Published:** 2026-07-17

**Authors:** Che Shen, Xiongfeng He, Zihao Li, Zidong Chen, Jiajin Sun, Xinyu Jiang, Yunhui Zhang, Lizhang Wu, Ran Wang, Jingnan Lu, Bo Wang, Kezhou Cai, Baocai Xu

**Affiliations:** ^1^ Engineering Research Center of Bio‐process Ministry of Education Hefei University of Technology Hefei China; ^2^ College of Food Science and Technology Bohai University Jinzhou China; ^3^ School of Computer Sciences Universiti Sains Malaysia Gelugor Penang Malaysia

**Keywords:** bolus, deep learning, electroencephalogram (EEG), gas chromatography–ion mobility spectrometry (GC–IMS), grilled lamb skewers, oral processing, retronasal olfaction

## Abstract

Retronasal olfaction is central to food flavor perception, yet its multiscale mechanisms from the oral processing to the central nervous system lack systematic elucidation. This study employs grilled lamb skewers as a model, integrating multi‐dimensional data and simulations to construct a research paradigm tracing the pathway from bolus release to central perception. Results reveal that extended grilling time increases bolus hardness and chewing force, with the 12 min sample exhibiting a collision intensity of 1.128, establishing a dual‐kinetic profile for aroma release. Efficiently transported aroma molecules target the olfactory cleft region, with characteristic volatile compounds substantially enriched at 30 s of chewing. Electroencephalogram (EEG) analysis distinguishes basic neural encoding of isolated aroma from multisensory cross‐modal integration during eating, with enhanced coupling between prefrontal alpha asymmetry and midline theta power (*r* = 0.36), delineating an inferred three‐stage framework from peripheral input to reward‐associated cortical responses. This framework is proposed based on EEG spatiotemporal patterns and source localization results, providing a testable model for future mechanistic studies. A physics‐guided dual‐timescale cross‐field interaction network achieves high‐accuracy, interpretable prediction of retronasal aroma intensity, while a complementary multiphysics simulation reproduces the complete physical process from chewing to nasal transport. This study pioneers a unified framework integrating macro‐scale processing, meso‐scale bolus evolution, micro‐scale molecular transport, and neural responses, providing a transferable methodological paradigm for flavor science.

## Graphical abstract

Using grilled lamb skewers as a model system, this work builds a multiscale coupling framework from oral processing to retronasal aroma perception, reveals dual‐kinetic release patterns and Electroencephalogram‐characterized central encoding features, and proposes an interpretable physics‐guided deep learning model validated by multiphysics simulation, offering a transferable paradigm for flavor science.

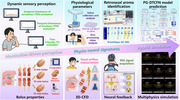

## 1 Introduction

Human perception of food flavor is fundamentally a dynamic process of multisensory integration. Within this process, retronasal olfaction plays a pivotal role. During chewing and swallowing, volatile compounds released from food are transported retrogradely through the nasopharynx to the olfactory epithelium. In the central nervous system, these olfactory signals undergo obligatory integration with taste signals, ultimately forming a unified flavor percept [1, 2]. Unlike orthonasal olfaction, which detects odors from the external environment, retronasal olfaction is primarily engaged during eating. The two pathways not only differ anatomically but also engage distinct perceptual and cognitive mechanisms [3, 4]. Critically, retronasal olfaction plays a decisive role in the holistic perception of food aroma [5]. A classic demonstration is the significant reduction in flavor perception—commonly described as food tasting bland—when nasal airflow is obstructed. This occurs because volatile compounds cannot reach the olfactory epithelium via the retronasal route. This phenomenon underscores the core function of retronasal olfaction in food recognition and flavor integration during oral processing. It also explains why retronasal olfaction serves as a key bridge connecting the food matrix, oral processing, and central nervous system perception.

However, the complexity of retronasal olfactory perception stems from its dynamic nature, which is regulated by multiple interacting factors. The perceived intensity and character of retronasal aroma are influenced by external factors, such as the physicochemical properties of the aromatic compounds and the composition and texture of the food matrix. They are also modulated by internal, individual physiological factors. These include chewing force and frequency, saliva secretion rate and composition, as well as oral and nasal airflow conditions [5, 6]. Saliva plays a particularly crucial role in this process. This is attributed to salivary mucins, which can bind to specific aroma compounds. This binding reduces their concentration in the oral headspace and alters their release kinetics. Consequently, saliva participates in flavor integration and can prolong the duration of aroma perception [7]. Furthermore, the surface area of the food bolus particles influences the rate of aroma release into the gaseous phase [8]. A particularly significant point is that the concentration of aroma compounds released during the oral processing of food can be 10 to 100 times higher than that perceived orthonasally from the same food source [2]. Therefore, compared to the orthonasal route, the retronasal pathway offers a distinct advantage in the perception and integration of a food's overall flavor [1, 9]. These complex regulatory factors are interwoven, spanning multiple scales from oral processing and molecular transport to neural encoding. This intricate interplay makes the mechanistic dissection of retronasal olfaction highly challenging. As a result, its dynamic principles and causal chains have not yet been systematically elucidated.

Elucidating the mechanisms of retronasal olfaction requires a research model with distinctive aroma characteristics, a representative oral processing pattern, and significant perceptual differences. Grilled lamb skewers aptly meet these criteria. As a quintessential traditional Chinese thermally processed meat product, its flavor formation depends on the Maillard reaction and lipid oxidation. During high‐temperature grilling, these reactions generate characteristic volatile compounds, including aldehydes, ketones, alcohols, as well as heterocyclic compounds like pyrazines and furans [10 ^,^ 11]. More importantly, the physicochemical changes within the food bolus during the mastication of grilled lamb skewers are significant. The associated retronasal aroma release exhibits rich and dynamic kinetics. This combination provides an ideal carrier for investigating the complete chain of events, from aroma release within the bolus to central nervous system perception. Therefore, systematically analyzing the formation and perception mechanisms of the retronasal aroma in grilled lamb skewers holds significant value. It not only contributes to understanding the fundamental flavor principles of traditional Chinese meat products but also offers an excellent research paradigm for uncovering the generalizable laws governing retronasal olfaction.

Despite the clear advantages of grilled lamb skewers as an ideal model for studying retronasal olfaction, existing research on their flavor has predominantly focused on static aroma analysis at the orthonasal level [12, 13, 14, 15, 16]. A systematic investigation into the characteristics and dynamic perception mechanisms of the retronasal aroma released during oral processing is still lacking. This gap is precisely where the key to understanding the perceptual mechanisms behind phenomena like “food tasting bland” may be found. Traditional sensory evaluation and analytical methods exhibit clear limitations for studying such dynamic and complex systems. In recent years, the rise of a suite of interdisciplinary techniques has created new possibilities to address this gap. For aroma compound analysis, gas chromatography–ion mobility spectrometry (GC–IMS) has been successfully applied to study retronasal aroma release in foods such as bread [2], cured meat [17], stewed beef [18], and yellow wine [19]. In characterizing perception dynamics, methods like time‐intensity (TI) and temporal dominance of sensations (TDS) can capture and quantify the temporal evolution of aroma perception in real time. At the level of neural mechanisms, Electroencephalography (EEG) offers a unique advantage in tracking rapid brain function changes related to olfactory perception, thanks to its millisecond‐level temporal resolution [14, 20]. Regarding the physiological basis, integrating computational fluid dynamics (CFD) with 3D nasal cavity reconstructions from CT scans allows for precise simulation of odorant molecule transport and distribution within the nasal cavity. This approach can mechanistically link aroma release to central nervous system perception [21]. Furthermore, the advent of deep learning and explainable learning paradigms helps overcome the limited interpretability of traditional “black‐box” models [22, 23]. These approaches enable the deep integration of physical prior knowledge governing food flavor perception with data‐driven methods. The potential outcome is high‐accuracy, highly interpretable prediction of these complex dynamic processes.

**SCHEME 1:**
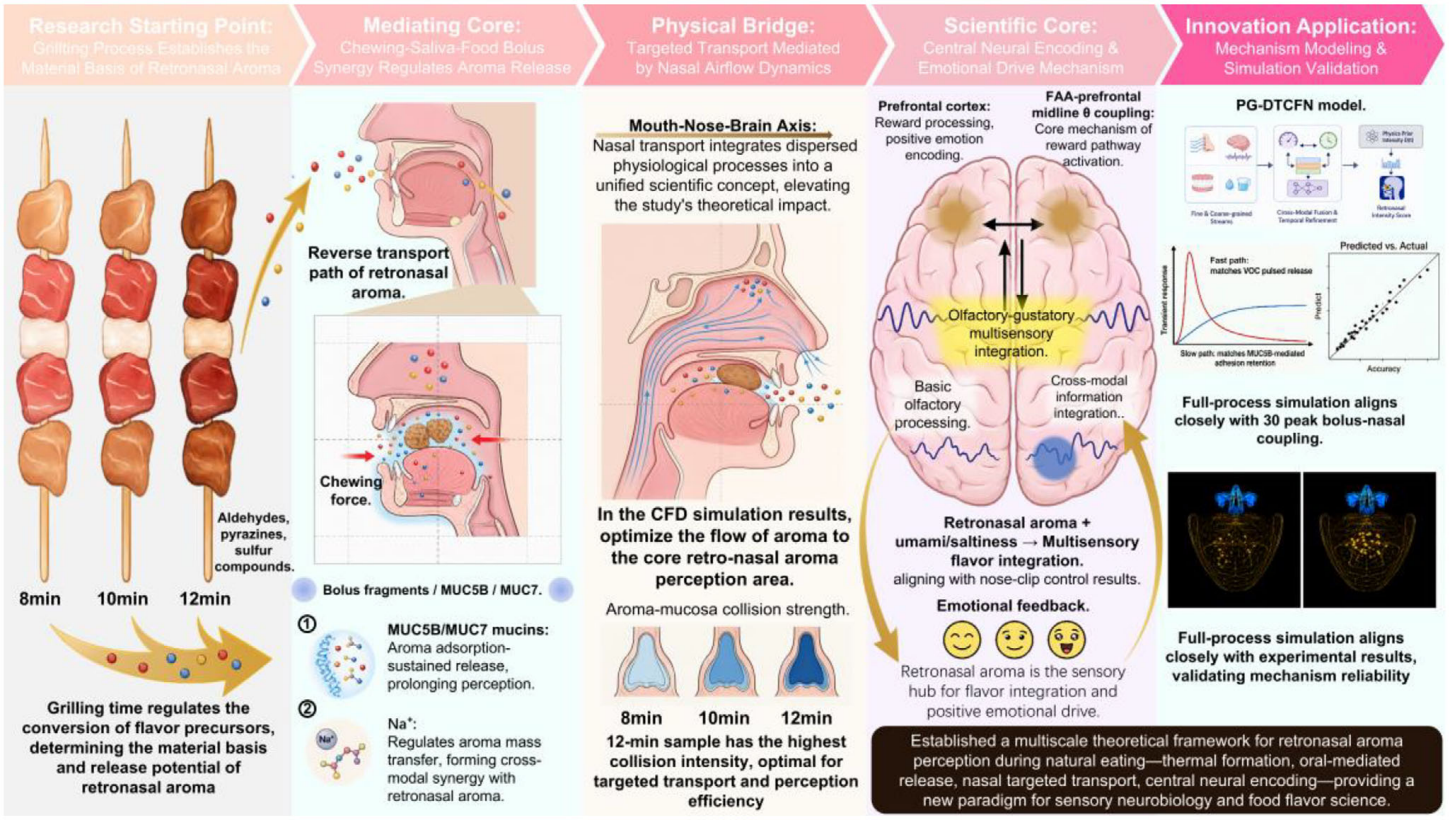
Schematic diagram of a multi‐scale research system integrating dynamic sensory perception, food bolus physicochemical characterization, oral behavior analysis, nasal gas simulation and volatile compound detection, EEG signal interpretation, deep learning modeling, and multiphysics simulation.

While the techniques have been applied across various food systems, most studies remain confined to a single dimension, lacking systematic integration across the complete perceptual chain. More importantly, the fusion of multi‐scale data and the mechanistic modeling spanning from oral processing to central nervous system perception remain significant challenges in the field of food sensory science. To address this, the present study moved beyond traditional single‐dimension paradigms (Scheme 1). For the first time, a comprehensive, multidimensional, and cross‐scale research framework has been constructed. Specifically, this study integrates TDS and TI methods to dynamically analyze the perceptual dominance and intensity changes of retronasal aroma. The regulatory mechanism of chewing behavior on bolus evolution and aroma release was elucidated by measuring bolus properties, including moisture, fat, and Na^+^ content, texture profile, and microstructural parameters. This was combined with analyses of saliva pH, mucin (MUC5B/MUC7) concentration, and video‐based quantification of chewing force and amplitude. A 3D nasal cavity model (based on the NasalSeg CT dataset) was combined with mastication respiratory waveforms to establish a synchronously coupled high‐frequency particle flow field model, quantifying aroma transport efficiency and olfactory mucosa collision intensity via CFD. The material basis of aroma release was identified by monitoring the volatile compound profiles in nasopharyngeal exhalate using GC‐IMS. Electroencephalography (EEG) coupled with SHapley Additive exPlanations (SHAP) explainable learning was employed to decode the central neural encoding of retronasal aroma perception and the associated activation of reward pathways. Furthermore, a physics‐guided dual‐timescale cross‐field interaction network (PG‐DTCFN) deep learning model was developed (Figure 7). This model enables high‐accuracy and highly interpretable prediction of retronasal aroma perception intensity. Simultaneously, a multi‐physics integrated simulation framework coupling chewing mechanics, bolus dynamic evolution, and nasal respiration was established.

Building on the preceding research framework, this study aims to systematically elucidate the formation and perception mechanisms of retronasal aroma using grilled lamb skewers as a model, specifically addressing three core scientific questions: how the thermal processing characteristics of the food matrix determine the release kinetics of retronasal aroma by regulating oral processing behavior and bolus evolution; what physical and chemical factors regulate the transport efficiency of aroma molecules in the nasal airflow field and how their spatial distribution correlates with perceived intensity; and how retronasal aroma signals achieve cross‐modal integration with taste signals in the central nervous system and drive positive emotional responses via reward pathways. By investigating these questions, this study seeks to construct a theoretical framework that integrates macro‐scale processing techniques, meso‐scale bolus evolution, micro‐scale molecular transport, and neural encoding into a unified system. This research not only provides a new theoretical paradigm for understanding the coupling principles between oral processing and olfactory perception but also offers scientific basis and computational tools for flavor‐oriented optimization of food processing techniques, flavor compensation strategies for low‐salt foods, and the design of personalized sensory experiences.

## 2 Results

### 2.1 Dynamic Sensory Perception of Grilled Lamb Skewers

#### 2.1.1 TDS and TDE Assessment for Grilled Lamb Skewers

**FIGURE 1:**
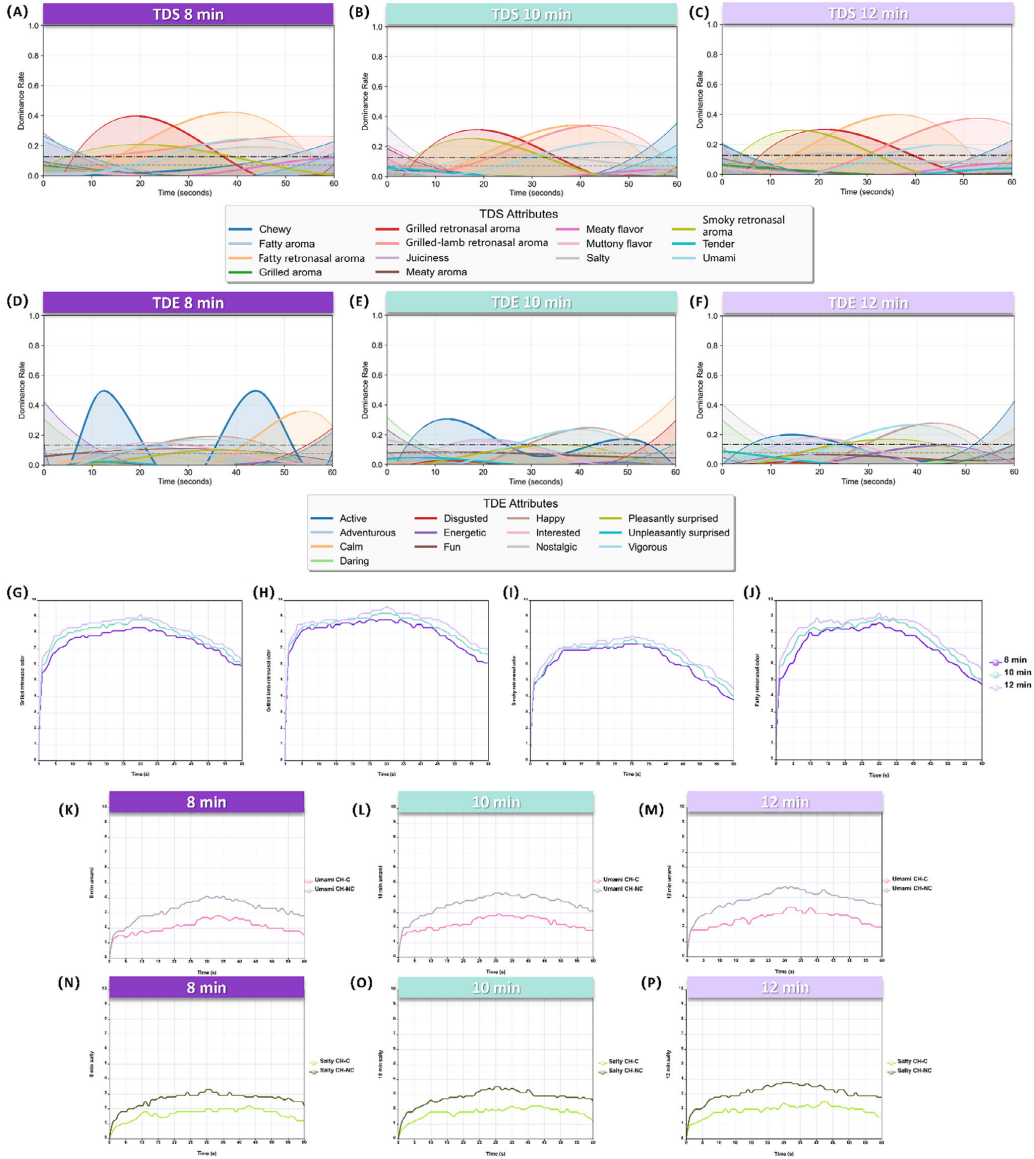
Dynamic sensory perception, emotional response, and retronasal olfaction‐mediated flavor enhancement of grilled lamb skewers with different grilling times. (A–C) Temporal Dominance of Sensations (TDS) analysis illustrated the changes in dominant attributes during the 60‐s chewing process of grilled lamb skewers with grilling times of 8 min (A), 10 min (B), and 12 min (C). (D–F) Temporal dominance of emotion (TDE) analysis illustrated the changes in dominant emotions during the 60‐s chewing process of grilled lamb skewers with grilling times of 8 min (D), 10 min (E), and 12 min (F). (G–J) Time‐intensity (TI) analysis of the dynamic perception intensity of four retronasal aroma attributes during mastication, including grilled retronasal aroma (G), grilled‐lamb retronasal aroma (H), smoky retronasal aroma (I), and fatty retronasal aroma (J), for grilled lamb skewers with 8, 10, and 12 min grilling times. (K–M) TI comparison of umami perception intensity between the nasal clip group (CH‐C, retronasal olfaction blocked) and non‐nasal clip group (CH‐NC, intact retronasal olfaction pathway) during mastication, for grilled lamb skewers with 8 (K), 10 (L), and 12 min (M) grilling times, verifying the umami‐enhancing effect of retronasal olfaction. (N–P) TI comparison of salty perception intensity between CH‐C and CH‐NC groups during mastication, for grilled lamb skewers with 8 (N), 10 (O), and 12 min (P) grilling times, verifying the saltiness‐enhancing effect of retronasal olfaction. (*Note*: The TDS opportunity line (gray dashed line) is 0.071, and the significance line (black dashed line) is 0.126. The TDE opportunity line is 0.076, and the significance line is 0.132.

As shown in Figure 1, Tables S1, and S2, TDS and TDE methods were employed, with significant lines set at 0.126 and 0.132, respectively. This analysis aimed to investigate the dynamic sensory changes and emotional responses during the 60‐s mastication of lamb skewers grilled for 8, 10, and 12 min.

Figure 1A,D showed that the 8‐min sample exhibited a diverse range of dominant sensory attributes. In the early chewing phase, attributes like Tender, Juiciness, and Meaty flavor were dominant. As chewing progressed, the four retronasal aroma attributes gradually became dominant. The Grilled‐lamb retronasal aroma became a dominant attribute as early as 11 s and remained so throughout mastication. The Fatty retronasal aroma (*DR*_max _= 0.4303, *AAS *= 1.28) and Grilled retronasal aroma (*DR*_max _= 0.4051, *AAS* = 0.97) were first perceived as significant (*T*_first_) before 16 s. Their durations of significant dominance (*D*_sig_) reached 39.66 and 31.08 s, respectively, establishing strong and sustained dominance from the early‐middle to late chewing stages. The Smoky retronasal aroma also showed stable, significant dominance (*AAS* = 0.71). Notably, the temporal windows of dominance for basic tastes like Salty and Umami overlapped highly with those of the retronasal aromas, indicating a multisensory synergy. In the late chewing phase, attributes like Chewiness, Tenderness, and Muttony flavor became dominant as the aroma diminished. Correspondingly, during the mid‐chewing phase dominated by retronasal aromas, the 8‐min sample elicited significant dominance of positive emotions such as Happy (*DR*_max _= 0.1923, *D*_sig _= 22.92 s) and Interested (*DR*_max _= 0.1572, *D*_sig _= 9.36 s). The peak and duration of these emotional responses aligned precisely with the peak periods of the retronasal attributes. This indicates that the rapid release of retronasal aroma, combined with taste synergy under short grilling time, effectively triggers basic positive emotional responses. Concurrently, moderate heat treatment preserved favorable texture and fundamental tastes.

Figure 1B illustrates that the 10‐min sample was also dominated by the four retronasal aroma attributes, accompanied by attributes like Chewiness, Juiciness, Tenderness, Meaty flavor, and Meaty aroma in the early and late chewing phases, as well as the taste attributes Umami and Salty. Specifically, the Grilled‐lamb retronasal aroma (*DR*_max _= 0.3625, *AAS* = 1.08, *D*_sig _= 38.07 s) formed a tri‐dominant pattern during the mid‐chewing phase with the Fatty retronasal aroma (*DR*_max _= 0.3636, *AAS* = 1.01, *D*_sig _= 35.23 s) and the Grilled retronasal aroma (*DR*_max _= 0.3350, *AAS* = 0.9, *D*_sig _= 28.27 s). The dominant perception and persistence of the Smoky retronasal aroma were also significantly enhanced compared to the 8‐min sample. This suggests that with chewing, the retronasal aromas become more prominent, transitioning from fatty and grilled notes toward characteristic lamb flavor, with the perceptual dominance extending further into the mid and late chewing stages. Correspondingly, the Happy emotion for the 10‐min sample appeared in the mid‐chewing phase, with its dominance window extending further into the mid‐late stages (Figure 1E). Its *DR*_max_ (0.2687) was higher than that of the 8‐min sample. Notably, the Pleasantly surprise emotion was perceived as dominantly significant for the first time, and its dominant region coincided precisely with the core dominance interval of the retronasal aroma attributes. This indicates that the increased richness and persistence of retronasal aroma perception due to longer grilling can further strengthen consumers' positive emotional responses and evoke a more diverse range of positive emotional experiences. This dominant perception profile is attributed to the prolonged grilling time, promoting the deeper conversion of characteristic lamb flavor precursors.

As shown in Figure 1C, the 12‐min sample demonstrated the strongest characteristic flavor dominance, the most sustained dominance across the entire cycle, and the broadest perceptual coverage. It was also absolutely dominated by the four retronasal aroma attributes, while attributes like Chewiness, Tenderness, Meaty flavor, Muttony flavor, Salty, and Umami also reached significant dominance levels. Among the aromas, the Smoky retronasal aroma and the Grilled retronasal aroma jointly constituted the dominant attributes in the early and mid‐chewing phases, jointly enhancing the complexity and persistence of retronasal aroma perception. Unlike the previous samples, the Smoky retronasal aroma was perceived significantly earlier (*T*_first _= 3.52 s), and its *DR*_max_ and AAS increased further to 0.2990 and 0.88, respectively, elevating it from a secondary feature to a core dominant attribute. Furthermore, the *DR*_max_ for the Grilled‐lamb retronasal aroma reached 0.3804 (AAS = 1.11, *D*_sig _= 38.07 s). Due to the enhanced dominance of the smoky aroma, the first significant perception of the grilled‐lamb aroma was delayed. The Fatty retronasal aroma (*DR*_max _= 0.4055, *D*_sig _= 36.92 s, AAS = 1.3) maintained its dominant role while broadening the overall perceptual duration window for the 12‐min sample. Interestingly, during the mid‐chewing phase, the synergistic effect between the basic tastes (Salty, Umami) and the retronasal aromas was strongest, creating a full and multi‐dimensional sensory experience. Correspondingly, during the mid‐chewing phase of the 12‐min sample, the *DR*_max_, duration, and AAS for positive emotions like Happy, Interested, and Pleasantly surprise were the highest among the three groups, with no significant negative emotional responses detected throughout the mastication (Figure 1F). The temporal characteristics of the positive emotions closely matched the full‐cycle dominance of the retronasal aroma attributes, suggesting that retronasal aroma perception is a key sensory pathway for eliciting pleasant emotions. The underlying reason is that sufficient heat treatment facilitates deep Maillard reactions and lipid oxidation. This leads to a comprehensive optimization of dominance and sustained advantage of the retronasal aroma attributes in the 12‐min sample, which synergizes with other attributes to form a more complex sensory experience. Therefore, retronasal aroma perception is the core sensory driver for consumers who sustained positive emotional responses.

#### 2.1.2 TI Assessment for Retronasal Aroma Attributes

Based on the TDS results, four retronasal aroma attributes were identified as dominant and sustained throughout the perception process. To clarify the dynamic perceptual characteristics of retronasal aroma during the mastication of lamb skewers, a time‐intensity (TI) analysis was further conducted. This analysis tracked the intensity changes of the four retronasal aromas (Fatty, Grilled, Grilled‐lamb, and Smoky) during chewing. As shown in Figure 1G–J, significant differences in these aroma attributes were observed among the lamb skewer samples grilled for different durations.

TI curve analysis revealed that all four retronasal aroma attributes exhibited a typical dynamic pattern across the three samples, characterized by a rapid rise, peak intensity during mid‐chewing, followed by a gradual decline. The peak intensity, rate of increase, and duration of perception were significantly dependent on grilling time. Compared to the other two samples, the 8‐min sample showed the lowest peak intensity for all aroma attributes. The perception of retronasal aroma during mid‐mastication was short‐lived, and the overall aroma release was insufficient and weak in persistence. The 10‐min sample demonstrated increased peak intensities compared to the 8‐min sample. Its mid‐mastication duration was prolonged, and the dynamic curves became smoother, indicating enhanced aroma fullness and stability. The 12‐min sample achieved the highest peak intensities for all attributes. It also exhibited the longest duration of perception during the mid‐chewing phase and maintained a high‐intensity perception throughout the entire mastication cycle. This indicates that the 12‐min sample, under the action of chewing, released aroma most completely and with the strongest persistence. Furthermore, a longitudinal comparison across all samples showed that the perception of retronasal aroma intensity peaked at 30 s of chewing. This timing likely corresponds to the promotion of bolus formation by chewing and saliva, which subsequently enhances the aroma release from the grilled lamb skewers.

To further clarify the synergistic mechanism between retronasal aroma and basic taste perception, a controlled experiment comparing a nose‐clip condition (CH‐C) and a no‐nose‐clip condition (CH‐NC) was conducted. The presence of retronasal aroma significantly enhanced the perceived intensity of both umami and saltiness. This synergistic effect was positively correlated with grilling time. For umami perception, the difference in perceived intensity between the CH‐NC and CH‐C groups increased significantly with longer grilling time. The difference was minimal for the 8‐min sample. It became noticeably larger for the 10‐min sample. In the 12‐min sample, the umami intensity in the CH‐NC group reached its peak, and the difference from the CH‐C group was most pronounced. This indicates that high‐intensity, long‐lasting retronasal aroma can synergistically enhance umami perception throughout the entire chewing cycle. A similarly significant synergistic enhancement was observed for saltiness perception. The effect of retronasal aroma on saltiness was relatively weak in the 8‐min sample. For the 10‐min sample, the saltiness intensity in the CH‐NC group was already significantly higher than in the CH‐C group. In the 12‐min sample, the saltiness intensity in the CH‐NC group peaked, showing the greatest difference from the CH‐C group. Notably, the Fatty and Smoky retronasal aromas formed a profound synergy with saltiness, significantly enriching the layering and persistence of salty perception.

The TDS and TI results clarified the perceptual dominance and intensity variation patterns of retronasal aroma, but the underlying material basis and physiological mechanisms still require further investigation. To this end, we conducted a systematic analysis from multiple dimensions, including the physicochemical properties of food bolus, oral behavior, nasal transport, volatile compound profiles, and central nervous system responses.

### 2.2 Characterization of Bolus Properties at Different Chewing Durations

**FIGURE 2:**
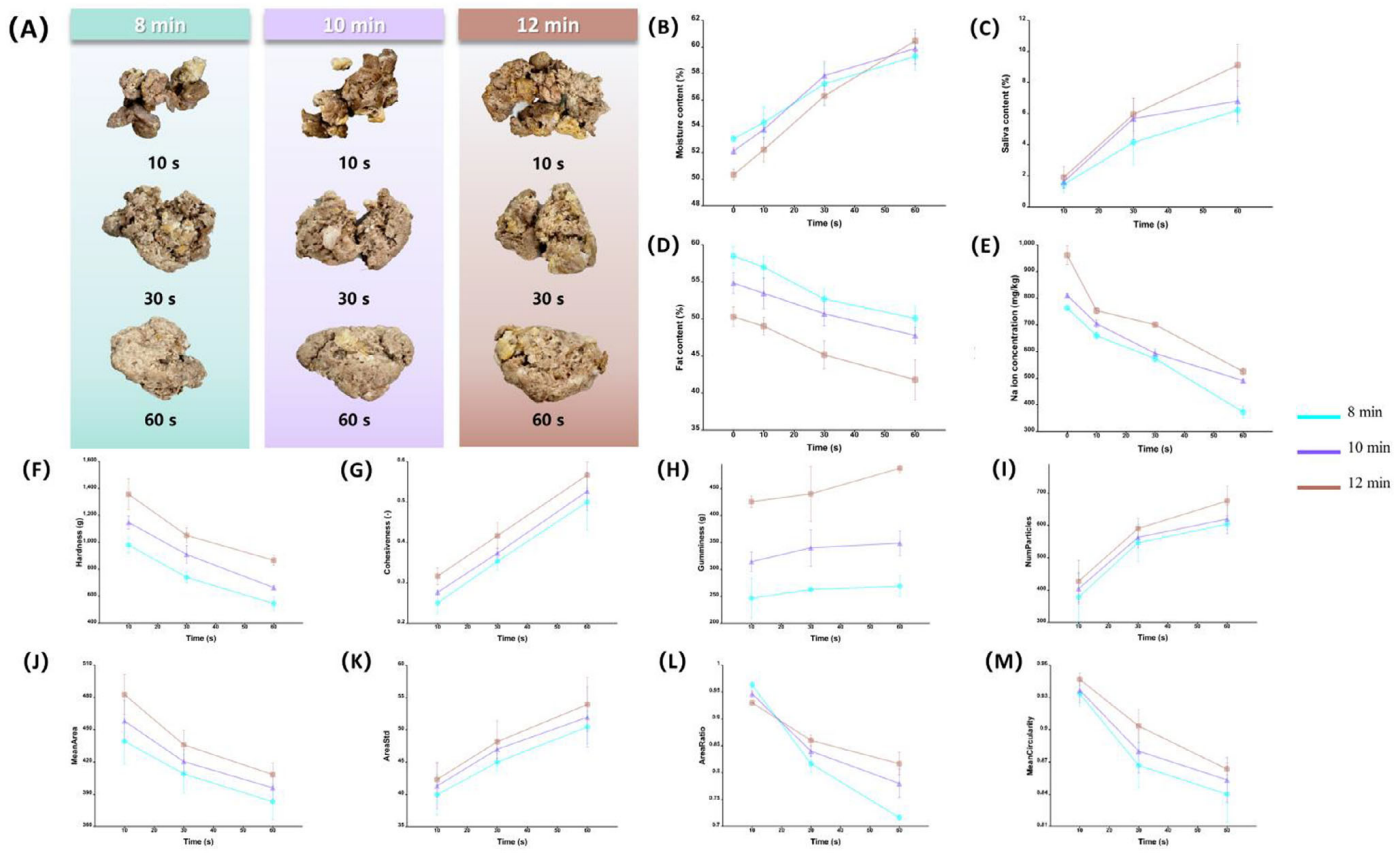
Dynamic evolution of bolus properties during oral processing of grilled lamb skewers. (A) Macroscopic morphological evolution of boluses from lamb skewers with different roasting times (8, 10, and 12 min) at three critical chewing time points (10, 30, and 60 s), visualizing the transition from mechanical fragmentation to homogeneous agglomeration. (B–E) Temporal changes in bolus physicochemical composition during 60 s mastication, including moisture content (B), saliva incorporation (C), fat content (D), and Na^+^ concentration (E). (F–H) Temporal evolution of bolus textural properties via texture profile analysis, including hardness (F), cohesiveness (G), and gumminess (H). (I–M) Quantitative analysis of bolus microstructure dynamics, including number of particles (I), mean particle area (J), standard deviation of particle area (K), area ratio (L), and mean circularity (M). The numerical values display the meaning, and the error bars represent the standard deviation.

To investigate the evolution of the bolus during the mastication of lamb skewers grilled for 8, 10, and 12 min, key chewing time points of 10, 30, and 60 s were selected to represent critical stages of bolus formation. As shown in Figure 2A, during the initial chewing phase (10 s), the lamb meat began to undergo mechanical breakdown through cutting and compression by the teeth, resulting in a loose and heterogeneous bolus. As chewing progressed (30 s), under the combined action of saliva lubrication and repeated tongue kneading, the fragmented meat particles gradually aggregated and homogenized, initially forming a bolus structure with a certain degree of cohesion. By the late chewing phase (60 s), the bolus underwent further integration. Its texture, viscosity, and lubricity stabilized, ultimately forming a bolus morphology suitable for swallowing. This process fully illustrates the dynamic structural evolution of the lamb skewer from fragmentation and mixing to final bolus formation.

Subsequently, the influence of grilling time and chewing behavior on bolus formation characteristics and quality attributes was explored from three aspects, including physicochemical composition, texture profile, and microstructure. The statistical analysis results of the responses were shown in Table S3. Analysis of physicochemical composition (Figure 2B–E) showed that with prolonged chewing time, the moisture content and saliva uptake of the bolus increased significantly across all three sample groups, with the 12‐min group showing the highest values at 60 s. Longer grilling time led to more complete protein denaturation and denser muscle fibers, thereby enhancing saliva adsorption capacity. Conversely, fat content and Na^+^ concentration decreased with extended chewing time, with the most significant reduction observed in the 12‐min group. This is related to the dissolution of fat and Na^+^ into the saliva phase during chewing. It also reflects that prolonged grilling accelerates thermal degradation and loss of fat (imparting a more intense fatty aroma) while enhancing the release and migration capacity of Na^+^ ions.

Texture profile analysis results (Figure 2F–H) indicated that bolus hardness decreased with longer chewing time. The 12‐min group exhibited the highest initial hardness at all points of time, which is associated with muscle fiber shrinkage and tissue densification caused by extended grilling. Cohesiveness and gumminess, however, increased with chewing time, with the 12‐min group showing the greatest increase. This suggests that the bolus formed from the 12‐min sample in the late chewing stage more readily formed a structurally stable, cohesive mass, closely related to the thorough hydration by moisture and saliva and the redistribution of proteins and fats.

Bolus microstructure parameters (Figure 2I–M) provided further insight into how chewing and saliva regulated the breakdown behavior of samples with different grilling times. The number of particles (NumParticles) increased with chewing time, consistently being highest for the 12‐min group, indicating that longer grilling made the meat more prone to breakage into finer particles. The mean particle area (MeanArea) decreased with chewing time, while the standard deviation of area (AreaStd) increased, reflecting a broadening particle size distribution. This change stems from the combined effects of fragmentation and aggregation during chewing. Initially, large fragments dominate, which gradually break down into smaller particles as chewing progresses. In the later stages, under the action of mucin, the fine particles aggregate into clusters, leading to minimize average area but increased size variability. The significantly higher AreaStd for the 12‐min group's bolus indicates stronger inter‐particle adhesion. Changes in the area ratio (AreaRatio) and mean circularity (MeanCircularity) showed that particle morphology became more regular and rounded with chewing. The 8‐min group exhibited the most pronounced morphological change due to its initially larger and more loosely packed particles. During the mastication of the 12‐min sample, more abundant mucin secretion facilitated the uniform coating and packing of fine particles into a dense bolus with clear boundaries.

In summary, systematic analysis of the bolus’ physicochemical composition, texture profile, and microstructure revealed that grilling time, by modulating the initial texture and chemical composition of the lamb meat, profoundly influenced the dynamic evolution of the bolus during chewing. The bolus formed from the 12‐min grilled sample exhibited higher initial hardness, greater saliva adsorption capacity, finer particle distribution, and higher cohesiveness. These characteristics collectively constitute the material foundation and structural carrier for aroma release. However, differences in bolus properties do not exist in isolation—they fundamentally reflect the adaptive response of oral processing behavior to lamb skewers with different grilling times. Chewing behavior, as the core driving link connecting bolus properties to aroma release, directly determines how consumers transform boluses with distinct physical properties into a unified flavor perception experience. Therefore, this study further quantified the oral behavior differences among subjects processing lamb skewers with different grilling times from two dimensions, salivary physiological parameters and chewing kinematics, to elucidate how bolus properties influence the release and perception of retronasal aroma by regulating chewing behavior.

### 2.3 Characterization of Oral Behavior

Saliva parameters, including pH, mucin content (MUC5B, MUC7), and sodium ion concentration, were measured to analyze physiological differences between resting and chewing states. This analysis aimed to clarify the regulatory role of chewing behavior in bolus formation and retronasal aroma release from grilled lamb skewers. As shown in Table S4, salivary pH increased significantly during chewing (*p* < 0.05), regardless of participant gender. The elevated pH can directly alter the acid‐base properties of the oral microenvironment, potentially promoting the hydrolysis and oxidation of aroma precursors within the bolus. Salivary mucins (MUC5B, MUC7), which are core glycoproteins, showed a significant chewing‐induced increase. As key components for oral mucosal adhesion and bolus structure formation [24], mucins are gradually released during chewing. Their increased content can enhance bolus viscoelasticity and stability, facilitating thorough mixing of the grilled lamb tissue with saliva to form a homogeneous bolus system. Sodium ions (Na^+^), which regulate saliva osmolality and ionic strength, are important factors linking taste perception to retronasal aroma release. The Na^+^ concentration also increased significantly during chewing, demonstrating its involvement in bolus formation and aroma release.

Chewing behavior is the core driver of food texture breakdown, bolus formation, and flavor release. Its dynamic characteristics directly influence sensory perception. The chewing process for 8‐min, 10‐min, and 12‐min grilled lamb skewers was analyzed via video using OpenCV. As shown in Figure S1, all three samples exhibited a typical dynamic pattern of chewing force during the 60‐s cycle, characterized by an initial rapid rise, mid‐term fluctuations, and a gradual decline. Both the peak chewing force and the mean force throughout the cycle increased significantly with longer grilling time.

The chewing force for all samples reached its first peak within the initial 10 s, with the 12‐min sample showing the highest peak value. During mid‐chewing (20–40 s), the 12‐min sample maintained a significantly higher force level than the 8‐min and 10‐min samples. A second peak occurred around 30 s, though the overall trend was a fluctuating decline. Even at swallowing readiness (60 s), the chewing force for the 12‐min sample remained significantly higher (*p *< 0.05), indicating a consistently greater chewing force demand.

Consistent with the chewing force trend, chewing amplitude and movement fluctuation characteristics also showed significant dependence on grilling time. The 8‐min sample maintained a low chewing amplitude throughout, with a maximum not exceeding 0.2 (Figure S2A). Movement fluctuations were small in amplitude and low in dispersion, showing a steady increase followed by a slow decline without sharp variations. The 10‐min sample exhibited significantly greater chewing amplitude and fluctuation intensity, with a maximum amplitude nearing 0.2 (Figure S2B). Movement dispersion increased noticeably during mid‐chewing, presenting more frequent fluctuations. The 12‐min sample reached the highest chewing amplitude among the groups (Figure S2C). Intense movement fluctuations appeared early (0–10 s), with a peak amplitude exceeding 0.3. The movement dispersion and fluctuation frequency throughout the cycle were significantly higher than for the other samples, stabilizing only toward the end of chewing.

These quantified oral behavior results provide essential behavioral baseline data for subsequent analysis of chewing‐driven nasal airflow dynamics and the neural response mechanisms underlying flavor perception.

### 2.4 Characterization of Nasal Gas

**FIGURE 3:**
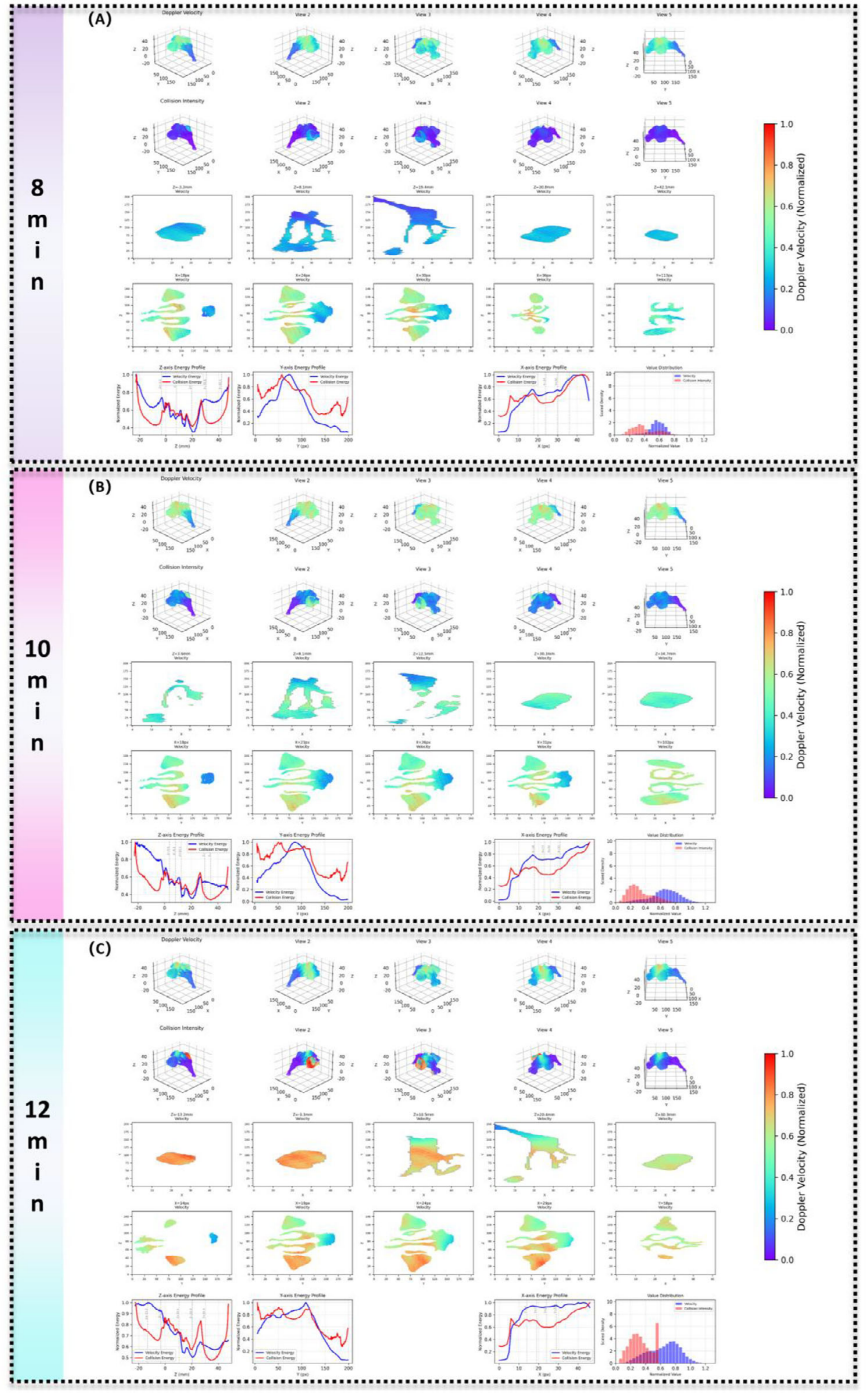
Computational fluid dynamics (CFD) simulation of intranasal airflow dynamics and aroma molecule transport during mastication of grilled lamb skewers with different grilling times. (A–C) Synchronously coupled high‐frequency particle flow field simulation based on a standardized 3D nasal cavity model reconstructed from 130 clinical head CT datasets, with boundary conditions calibrated using real‐time respiratory data measured during mastication via a respiratory flow sensor. (A) Simulation results for the 8 min grilled lamb skewers. Panels from top to bottom: 3D visualization of intranasal airflow Doppler velocity field; 3D spatial distribution of collision intensity between aroma molecules and nasal mucosa; *Z*‐axis cross‐sectional distribution of airflow velocity; *Z*‐axis cross‐sectional distribution of collision intensity; axial energy profiles (*Z‐*axis, *X*‐axis); and normalized value distribution histograms of airflow velocity and collision intensity. (B) Simulation results for the 10 ‐ min grilled lamb skewers, with an identical panel layout to (A), illustrating the enhanced aroma transport efficiency and molecule‐mucosa collision effect induced by prolonged grilling time. (C) Simulation results for the 12 ‐ min grilled lamb skewers, with an identical panel layout to (A), demonstrating the highest collision intensity, most efficient targeted transport to the olfactory cleft (the core functional region for retronasal olfaction), and most stable airflow dynamics among all groups.

A synchronously coupled high‐frequency particle flow field model, based on a 3D‐CFD system, was employed to characterize the nasal airflow patterns and aroma molecule transport during the mastication of lamb skewers grilled for 8, 10, and 12 min (Figure 3). This aimed to establish the mechanistic link between the bolus formed during oral processing, retronasal aroma release, and olfactory perception for different grilling times. The study analyzed two core physical quantities for the three grilling durations. These quantities were the Doppler velocity, which represents the nasal airflow velocity field, and the collision intensity, which quantifies the probability of aroma molecules colliding with the nasal mucosa. Visualization was achieved through multi‐view 3D rendering, axial cross‐sections, and energy distribution analysis. The key simulation parameter results were shown in Table S5.

For the 8‐min sample, aroma molecules exhibited a distinct spatial distribution pattern during retronasal perception (Figure 3A). 3D visualization showed that airflow interaction and molecular collisions with the mucosa were concentrated mainly in the mid‐to‐upper nasal cavity (*Z *≈ 20–40 mm). This region corresponds closely to the anatomical location of the olfactory cleft, identifying it as the core functional area for retronasal perception. *Z*‐axis horizontal slice analysis indicated that the cross‐section at *Z* = 19.4 mm had the widest airflow distribution and the most significant velocity gradient, serving as an efficient channel for aroma delivery to the retronasal olfactory region. Airflow was sparse in the lower regions (*Z* < 0 mm), resulting in weaker aromatic effects, and contracted in the upper regions (*Z* > 40 mm), leading to reduced perceptual activity. Axis energy profiles revealed that the peaks for airflow energy and collision energy were located at *Z* = 22.5 mm and *Z* = 22 mm, respectively. Their high degree of overlap confirmed that *Z *≈ 22 mm is the precise height at which the interaction between aroma molecules and the olfactory mucosa is strongest. The *X*‐axis energy profile showed the airflow energy peak was at *X* ≈ 35 px, corresponding to the mid‐posterior segment of the nasal cavity. As shown in Table S5, the mean collision intensity (0.220) was lower than the mean airflow velocity (0.310). Collision intensities were primarily distributed in the 0.2–0.6 range, while velocity energies concentrated in the 0.4–0.6 range. This suggests that sustained molecular collisions at low‐to‐medium intensity are the dominant driver of aroma perception. In summary, the key region for retronasal perception of the 8‐min sample is the overlapping area of the mid‐to‐upper nasal cavity (*Z* ≈ 20–25 mm) and the mid‐posterior segment (*X* ≈ 30–40 px). The aerodynamic characteristics of this region provide the structural basis for the efficient capture and perception of aroma molecules.

Compared to the 8‐min sample, the 10‐min sample shared the same core spatial distribution pattern but exhibited subtle differences in energy peaks and intensities (Figure 3B). 3D visualization results maintained the core positioning in the mid‐to‐upper nasal cavity (*Z* ≈ 20–40 mm). *Z*‐axis horizontal slices indicated the efficient aroma transport channel corresponded to the *Z* = 12.5 mm cross‐section, while the airflow characteristics in the lower (*Z *< 0 mm) and upper (*Z* > 35 mm) regions remained consistent with the 8‐min sample. Axis energy profile analysis revealed that the airflow energy peak (*Z* = 20.6 mm) and the collision energy peak (*Z *= 22 mm) for the 10‐min sample still overlapped height. Compared to the *Z *≈ 22.5 mm peak position of the 8‐min sample, this represented a slight anterior shift, confirming *Z *≈ 21–22 mm as the precise height of strongest aromatic action. The *X*‐axis energy profile showed energy peaks remained concentrated in the *X* ≈ 30–40 px interval, and the *Y*‐axis profile indicated energy peaks were distributed in the *Y *≈ 50–150 px range, maintaining a stable anterior–posterior distribution of aroma within the nasal cavity. Furthermore, both the mean collision intensity (0.651) and mean airflow velocity (0.329) for the 10‐min sample were higher than those for the 8‐min sample. This indicates that the bolus from lamb skewers grilled for a longer time had more full contact with the oral mucosa during chewing, leading to significantly enhanced aroma release and molecular collision. Histograms of numerical distribution showed a shift of collision intensity toward higher value intervals, further corroborating the increase in aroma perceptual activity. In summary, the key retronasal perception region for the 10‐min sample is consistent with that of the 8‐min sample. Chewing lamb skewers grilled for a longer duration strengthened molecular collisions, thereby improving olfactory perceptual efficiency in this core region.

Compared to the 8‐min and 10‐min samples, the 12‐min sample‐maintained stability in its core spatial localization (Figure 3C). However, it exhibited further evolution in energy peaks, collision intensity, and distribution characteristics. The 3D visualization results confirmed the sustained localization of the core functional region in the mid‐to‐upper nasal cavity (*Z* ≈ 20–40 mm). The *Z*‐axis horizontal slice corresponding to the efficient aroma transport channel was identified at the *Z* = 20.4 mm cross‐section. In this layer, the airflow distribution range, velocity gradient, and intensity all surpassed those of the previous two samples. While airflow contraction persisted in the lower (*Z* < 0 mm) and upper (*Z* > 30 mm) regions, the airflow intensity was significantly enhanced. Axis energy profile analysis revealed a slight increase in the vertical separation between the airflow energy peak (located at *Z* = 13.2 mm) and the collision energy peak (located at *Z* = 22.5 mm) for the 12‐min sample. Notably, the collision energy peak coincided precisely with that of the 8‐min sample. In contrast, the airflow energy peak shifted slightly toward the lower nasal cavity. This shift reflects subtle adjustments in the aroma molecule transport pathway resulting from the longer grilling time. The *X*‐axis energy profile indicated a broadening of the energy peak interval to *X *≈ 25–40 px, suggesting a more uniform lateral distribution of aroma within the nasal cavity. The *Y*‐axis energy profile maintained a stable distribution within the *Y *≈ 50–150 px range. As detailed in Table S5, the 12‐min sample demonstrated significantly higher mean collision intensity (1.128) and mean airflow velocity (0.591) than the previous two samples. Combining this with the established finding that volatile compound concentration increases with longer grilling time suggests that the 12‐min sample released aroma more completely during chewing [14]. This led to a further enhancement of molecular collisions, which significantly improved olfactory perceptual efficiency and intensity. Furthermore, the numerical distribution histogram showed a clear shift of collision intensity toward the 0.6–1 interval. This observation further corroborates the trend that aroma perceptual activity strengthens with extended grilling time. In summary, the key region for the retronasal perception of the 12‐min sample remained the overlapping area of the mid‐to‐upper nasal cavity (*Z *≈ 20–25 mm) and the mid‐posterior segment (*X *≈ 25–40 px). The mastication of lamb skewers grilled for the longest duration enhanced collision intensity and broadened the lateral distribution range. These effects collectively served to further optimize the efficiency of aroma capture and perception within this core region.

To assess whether the 10% uncertainty in inlet velocity (as calibrated against the airflow sensor) affects the spatial localization of aroma–mucosa collisions, we performed an ablation analysis by perturbing the measured inlet velocity envelope by −10% and +10% while keeping all other simulation parameters identical. As shown in Table S6, when the inlet velocity was decreased by 10% (from the baseline 1.84 to 1.66 m/s) or increased by 10% (to 2.02 m/s), the collision hotspot (region of highest collision intensity) remained stable within the mid‐to‐upper nasal cavity: the *Z*‑axis position of the hotspot varied only between 22.1 and 22.9 mm (baseline: 22.5 mm), and the *X*‑axis position varied between 31 and 33 px (baseline: 32 px). The normalized collision intensity maps under both perturbations maintained high spatial similarity to the baseline simulation, with map correlations of 0.983 (–10%) and 0.978 (+10%). These results demonstrate that the conclusion regarding the spatial localization of retronasal aroma transport (i.e., the olfactory cleft region, *Z* ≈ 20–25 mm, *X* ≈ 25–40 px) is robust against the 10% uncertainty in inlet velocity.

The nasal gas simulations revealed the spatial localization and intensity characteristics of collisions between aroma molecules and the olfactory mucosa. They clarified the differences in the transport efficiency of aroma molecules within the nasal flow field for samples with different grilling times. However, increased collision intensity is merely the physical prerequisite for aroma perception. The specific aroma molecules that reach the olfactory epithelium and how their identity and abundance change with grilling time still require clarification at the material level. Therefore, to address this, volatile compounds exhaled from the nasopharynx during the mastication of lamb skewers with different grilling times were further captured and analyzed.

### 2.5 Determination of VOCs Profiles During Oral Processing by GC‐IMS

Retronasal aroma components exhaled from the nasopharynx were monitored using a BreathSpec GCIMS. A total of 48 volatile organic compounds (VOCs) were detected and semiquantified, including monomers and dimers. Their peak volume data are shown in Table S7. All peak volumes are expressed in arbitrary units (a.u.), representing the integrated ion signal intensity over retention time and drift time. These compounds mainly consisted of aldehydes (10), ketones (12), alcohols (11), esters (9), pyrazines (3), an acid (1), and a sulfur ‐ containing compound (1). For lamb skewers with different grilling times, most signals of VOCs appeared in the retention time range of 100–300 s and the drift time range of 1‐1.8 ms. Quantitative analysis of peak volumes revealed a dynamic pattern: aroma substances started to be released at 10 s of chewing, reached their peak at 30 s, and significantly decayed by 60 s. For most VOCs, the peak volume at 30 s was significantly higher than that at 10 and 60 s (*p* < 0.05). This indicates that both the variety and concentration of aroma substances released via the retronasal route reached their maximum during the middle stage of chewing. This pattern was consistent across all grilling time groups. For example, in the 12‐min grilling group, the peak volume of ethyl formate‐M was 345.30 at 10 s, increased to 654.93 at 30 s, and then dropped sharply to 100.65 at 60 s (*p* < 0.05). In the same group, 3‐methylbutanal also increased from 305.31 (10 s) to 686.47 (30 s) and then decreased to 119.16 (60 s).

The peak volumes of retronasal aroma substances differed significantly among lamb skewers with different grilling times (Figure S3B,C; Table S7). Overall, the concentration tended to increase with longer grilling time. The 12‐ min grilling group showed the greatest diversity of aroma substances and generally the highest signal intensity at each chewing time point. At 10 s of chewing, some substances in this group already had significantly higher release levels. For instance, 2‐propanethiol dimer reached 707.07 a.u., which was significantly higher than in the 8 and 10 min groups (*p* < 0.05). At the 30 s peak period, this group uniquely exhibited the highest abundances of several key substances, including ethyl formate‐M and ‐*D*, 3methylbutanal, 2methyltetrahydrofuran3one, ethyl 2methylpropanoate, and 2propanethiol*‐D*. The 10‐min grilling group showed stronger release than the 8 min group. For some substances, such as 2‐ethylpyrazine and dimethyl disulfide, the maximum values occurred at 30 s and were significantly higher than those in the 8‐ and 12‐min groups at the same time point (*p* < 0.05). Although the overall release levels in the 8‐min group were lower, VOCs such as 2‐methylbutanoic acid still showed a clear and significantly highest peak at 30 s of chewing, confirming that a midchewing release peak is an inherent temporal feature.

Across all three grilling groups, most aroma substances showed their largest peak volume at 30 s of chewing, and by 60 s the values generally returned to levels close to or below those at 10 s. The differences in abundance and temporal profiles of characteristic aroma signals among the grilling groups were highly consistent with the differences in retronasal attribute intensity observed in the TDS and TI experiments. This further demonstrates that grilling time, by modulating flavor formation processes such as lipid oxidation and the Maillard reaction during thermal processing of lamb, alters the timing and amount of aroma substances released during chewing. The large release of retronasal aroma substances at the 30 s peak, as confirmed by quantitative peak volume analysis, likely constitutes the core material basis driving the strong retronasal aroma perception during the middle chewing stage.

Nevertheless, physical impaction of volatile compounds on the olfactory epithelium is only the starting point of perception. How these quantitative differences at the material level are converted into signals recognizable by the central nervous system, and thereby drive flavor enhancement and emotional responses, still needs to be elucidated from the perspective of neural coding.

### 2.6 EEG Analysis of Grilled Lamb Skewers With Different Grilling Times

To clarify the central neural encoding mechanisms underlying the perception of retronasal aroma in grilled lamb skewers, electroencephalography (EEG) combined with SHapley Additive exPlanations (SHAP) interpretable learning was employed. The analysis focused on the spectral and spatiotemporal dynamic characteristics of EEG signals from participants under three conditions: CH‐NC (eating without intervention), CH‐C (wearing a nose clip to block retronasal aroma perception), and CH‐R (input with artifacts removed, retaining only the retronasal aroma‐related EEG signal). Participants consumed lamb skewers grilled for 8, 10, and 12 min. To validate the specificity of the CH‑R signals, we performed additional control experiments based on our previously established EEG paradigms. Orthonasal smelling (without eating) and odorless texture‐matched chewing confirmed that CH‐R signals were specifically attributed to retronasal aroma, rather than orthonasal input or somatosensory/chewing artifacts (Figures S4,S5). The study therefore aimed to quantify the contribution of different EEG channels to the perception of retronasal aroma, thereby providing electrophysiological evidence for how retronasal aroma modulates flavor perception and positive emotional responses.

#### 2.6.1 EEG Neural Response Characteristics of Retronasal Aroma Perception and Identification of Core Regulatory Channels

Analysis of the area under the curve (AUC) of power spectral density (PSD) in Figure 4A showed that the PSD‐AUC in the low‐frequency bands (*δ* and *θ* bands) differed most significantly between the CH‐NC and CH‐C conditions. These bands had the highest discriminatory power for distinguishing the two conditions, followed by the *α* and *β* bands, with the γ band contributing the least. This indicates that low‐frequency EEG oscillations are core neural markers reflecting retronasal aroma input. Frontal alpha asymmetry (FAA), a key indicator of reward processing and positive emotional response, exhibited significantly condition‐dependent temporal dynamics. As shown in Figure 4B, the FAA curves for the CH‐NC and CH‐C conditions diverged clearly throughout the entire chewing cycle. The between‐group differences were most pronounced for the 8‐min and 12‐min samples. This demonstrates that sustained retronasal aroma input can significantly alter the neural oscillation patterns in the prefrontal cortex during eating. Furthermore, this alteration is most evident at grilling times that produce the most representative flavor characteristics. Correlation analysis (Figure 4C) further revealed a statistically significant correlation between FAA and prefrontal midline θ power under the CH‐NC condition (*r* = 0.36, 95% CI: 0.19–0.51), whereas no significant correlation was observed under the CH‐C condition (*r* = 0.10, 95% CI: −0.08–0.27). To statistically compare the two conditions, a Fisher's *z*‐test was performed using a conservative effective sample size (n_eff = 120 per condition, accounting for repeated 10‑s windows across participants and chewing cycles). The difference between the two correlation coefficients was significant (Delta *z* = 0.277, *Z* = 2.12, *p* = 0.034; Table S8). This differential pattern indicates that retronasal aroma input strengthens the coupling between reward‐related EEG markers (left‑lateralized FAA) and attention/emotional processing markers (midline *θ*). Left‑lateralized FAA has been established in the literature as an electrophysiological index of approach motivation and positive affect [25, 26, 27, 28]. The enhanced FAA‑*θ* coupling under retronasal aroma input therefore suggests that retronasal aroma contributes to the neural processes underlying positive emotional responses during eating. The spatiotemporal distribution characteristics of the EEG signals further revealed the core brain regions regulating retronasal aroma perception. The brain topographic maps in Figure 4D show that under the CH‐NC condition, the spatial distribution of EEG power was more stable across the six‐time windows of the chewing cycle. Notably, the EEG responses were stronger during the 20–30, 30–40, and 40–50 s time windows, aligning with previous sensory evaluation and GC‐IMS results. In contrast, the power distribution under the CH‐C condition showed greater spatiotemporal variability. This indicates that the continuous input of retronasal aroma provides the central nervous system with stable multisensory signals, resulting in a more stable spatiotemporal encoding pattern of the EEG response. Blocking retronasal aroma leaves only chewing and taste signals, which fail to form a stable neural code, leading to significantly increased dispersion in the central response. The summarized results of the full‐band power spectrum features (Figure 4E) and the AUC by brain region (Figure 4F) further verified that θ‐α band power in the frontal and central regions is the core neural feature distinguishing the CH‐NC and CH‐C conditions. These two brain regions are core functional areas for olfactory‐gustatory multisensory integration, oral motor perception, and flavor reward encoding. This clarifies the regulatory mechanism from peripheral sensory input to central neural integration.

FIGURE 4Neurophysiological coding mechanism underlying retronasal olfaction‐mediated flavor perception: spatiotemporal electroencephalogram (EEG) characteristics and interpretable machine learning dissection. (A) Power spectral density (PSD) analysis across five EEG frequency bands (delta, theta, alpha, beta, gamma) comparing the intact retronasal pathway (CH‐NC) and blocked pathway (CH‐C) conditions. (B) Temporal dynamics of frontal alpha asymmetry (FAA) during 60 s mastication for 8, 10, and 12 min samples under CH‐NC and CH‐C conditions. (C) Correlation between FAA and frontal‐midline theta power: moderate positive correlation in CH‐NC (*r* = 0.36) versus markedly weakened correlation in CH‐C (*r* = 0.10). (D) Spatiotemporal scalp topographies across sequential 10 s windows of the 60 s mastication period for CH‐C (left) and CH‐NC (right) groups. Continuous retronasal input (CH‐NC) yields stable spatial power distribution, while its absence (CH‐C) leads to greater spatiotemporal variability. (E) Averaged PSD curves and corresponding scalp topographies for *δ*, *θ*, *α*, *β*, and *γ* bands. (F) Summarized PSD‐AUC across five functional brain regions (frontal, parietal‐occipital, left temporal, central, right temporal). (G–K) SHAP‐based interpretable analysis: (G) channel importance ranking; (H) temporal waterfall plot of SHAP values across 60 s; (I) summary bar plot of channel contributions; (J) spatiotemporal SHAP heatmaps comparing CH‐C and CH‐NC; (K) channel‐level SHAP scatter plot quantifying positive (red) and negative (blue) contributions to retronasal perception.

**Figure:**
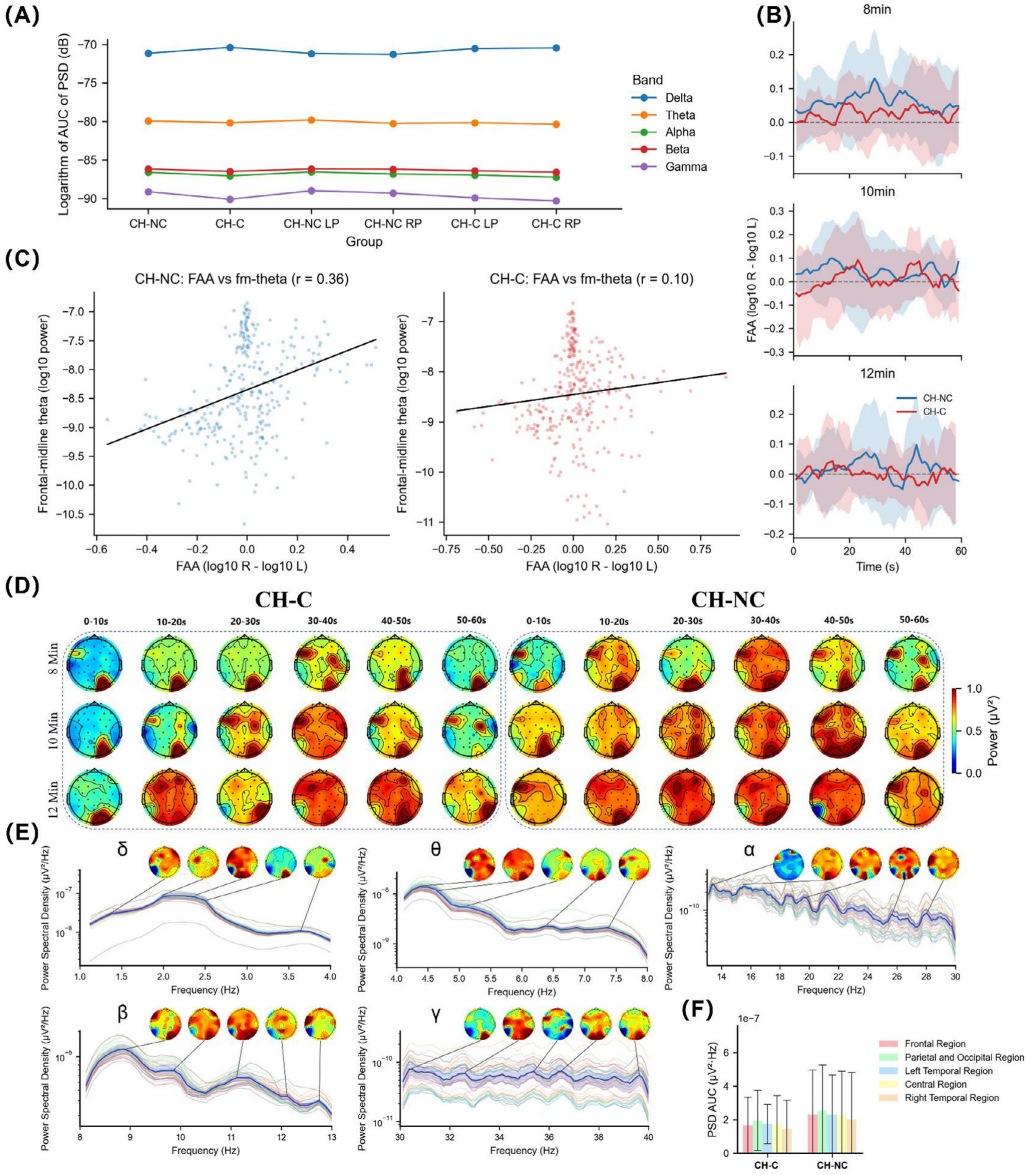

**Figure:**
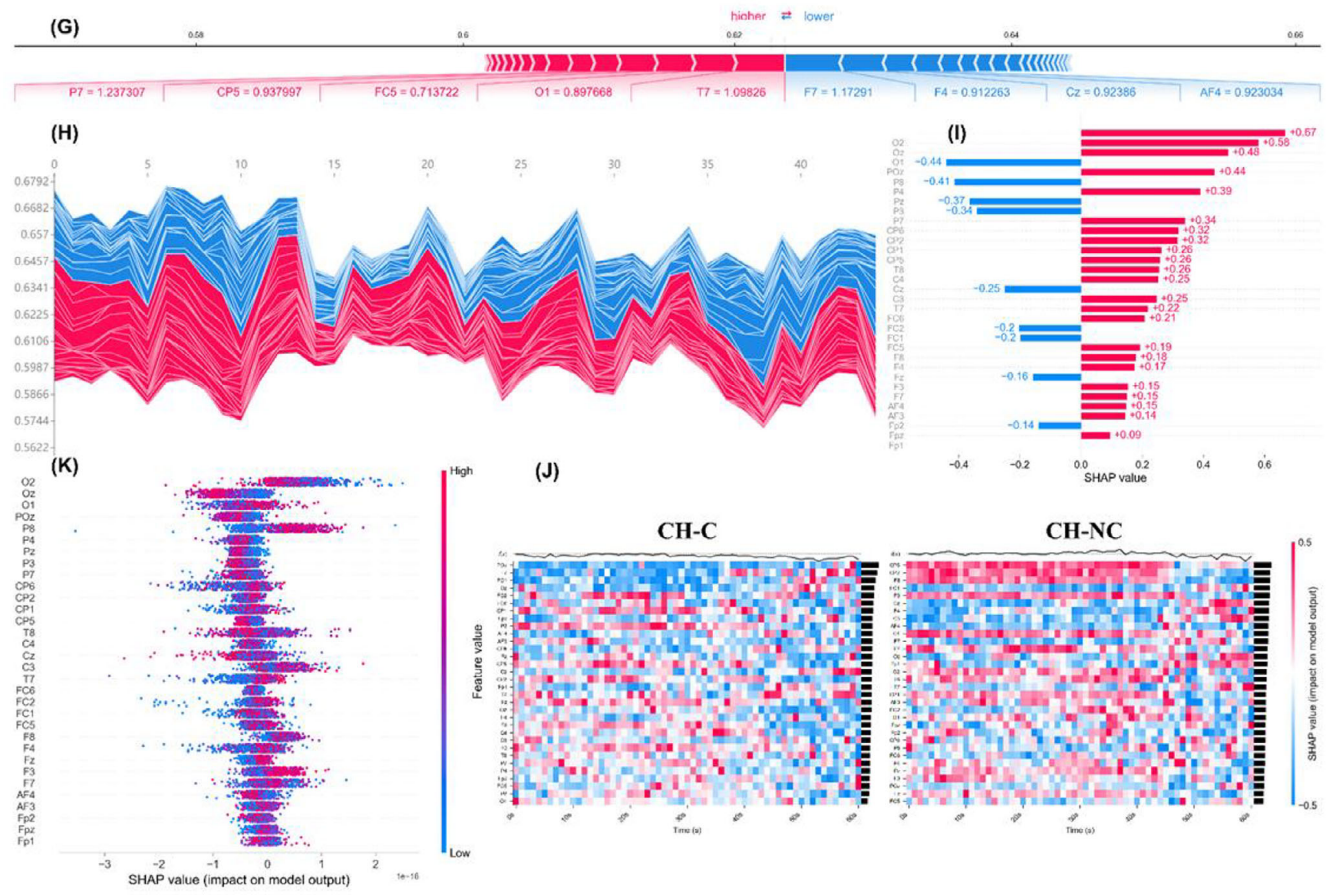

To localize the cortical sources underlying these scalp EEG patterns, source localization analysis was performed (Figure S6). Under the CH‑NC condition, widespread cortical activation was observed across the frontal, parietal, temporal, occipital, and cingulate regions, and the activation intensity increased progressively with grilling time. In contrast, the CH‑C condition showed markedly reduced activation, with only weak residual activity in the frontal, temporal, and cingulate areas. The correlation‑based connectivity maps further revealed that CH‑NC engaged extensive inter‑regional coherence, whereas CH‑C exhibited sparse connectivity. Consequently, the estimated cortical energy distribution was broad in CH‑NC (fronto‑parieto‑temporal‑occipital network) but contracted in CH‑C, mainly confined to the frontal cortex. These source‑level results corroborate the scalp findings and demonstrate that retronasal aroma input drives a wide cortical network involving sensory, integrative, and reward‑related regions.

Based on SHAP interpretability analysis, this study quantified the contribution of different EEG channels to the perception differences in retronasal aroma. Single‐channel analysis of variance showed that channels such as T7, F7, F4, and AF4 had the highest *F*‐values, identifying them as core channels for distinguishing the CH‐NC and CH‐C conditions (Figure 4G). Among these, the left temporal lobe and left prefrontal cortex (T7, F7) are core brain regions for olfactory processing and multisensory integration, while the right prefrontal cortex (F4, AF4) is closely related to reward and positive emotion encoding. The temporal distribution of SHAP values (Figure 4H) showed that the contribution of core channels persisted throughout the entire 60‐s chewing cycle. The positive contribution was most prominent during mid‐chewing (15–45 s), which aligns perfectly with the time characteristics of peak retronasal aroma intensity in the TDS and TI results. This confirms a high degree of temporal consistency between central neural responses and peripheral sensory perception.

The SHAP analysis identified occipital and parietal channels (O2, Cz, PO8, and P8) as having the highest contribution to the model output for distinguishing CH‐NC from CH‐C conditions (Figure 4I). At the scalp level, this finding indicates that these electrode positions are particularly sensitive to the presence of retronasal aroma input. The occipital‐parietal regions, although traditionally associated with visual processing, have been shown to participate in cross‐modal attention and multisensory integration even in the absence of visual input [29]. In the context of closed‐eye chewing, alpha oscillations recorded from occipital electrodes can reflect top–down attentional modulation rather than visual inhibition. Thus, the high contribution of these channels is likely to reflect the engagement of attentional and integrative networks during retronasal aroma perception. The SHAP heatmap (Figure 4J) and the beeswarm plot (Figure 4K) provided supplementary verification. Under the CH‐NC conditions, the feature contributions of the EEG channels showed a more regular and concentrated pattern of high contribution. This further proves that the presence of retronasal aroma makes the central neural encoding pattern for flavor perception more stable and focused.

#### 2.6.2 Analysis of Central Neural Response Characteristics to Isolated Retronasal Aroma Input and Its Multisensory Integration Mechanism

To clarify the central neural encoding principles of retronasal aroma itself by isolating the interference from chewing movement and oral taste input, EEG signals under the CH‐R condition were analyzed. As shown in Figure 5A, isolated retronasal aroma input significantly increased the PSD‐AUC levels across all frequency bands. The increase was most pronounced in the low‐frequency *δ* and *θ* bands, which exhibited the highest discriminatory power for different grilling times. This pattern completely aligns with the spectral characteristics observed under the CH‐NC condition. This indicates that retronasal aroma achieves perceptual encoding at the central level by modulating low‐frequency oscillations, regardless of whether it is accompanied by oral chewing and taste input. FAA analysis revealed a weak, non‐significant linear correlation (*r* = 0.06) between FAA and prefrontal midline *θ* power under the isolated retronasal aroma input (Figure 5B). In contrast, this correlation was stronger (*r *= 0.36) under the CH‐NC condition and diminished (*r *= 0.10) when retronasal aroma was blocked (CH‐C). This difference clarifies that the multisensory integration of retronasal aroma with taste and chewing movement is the core prerequisite for strengthening the neural coupling between reward‐related responses and attentional processing. Isolated retronasal aroma primarily drives basic olfactory perceptual encoding. Its cross‐modal synergy with taste, however, is necessary to activate stronger reward pathways. This provides a neural mechanism‐level explanation for the earlier finding that retronasal aroma synergistically enhances positive emotional responses with saltiness and umami. The FAA temporal dynamics in Figure 5C show that under isolated aroma input, the 12‐min sample exhibited the largest FAA fluctuation amplitude and the most significant deviation from baseline. This directly corresponds to the behavioral result that the 12‐min sample elicited the strongest retronasal aroma perception and positive emotional response under the eating condition. It demonstrates that the enhanced retronasal aroma characteristics resulting from optimized grilling time can, by themselves, drive stronger prefrontal reward‐related neural responses.

FIGURE 5EEG dynamics and interpretable decoding of isolated retronasal olfactory input (CH‐R) in the absence of oral somatosensory and gustatory cues. (A) Power spectral density (PSD) analysis across five EEG frequency bands (Delta, Theta, Alpha, Beta, and Gamma) under isolated retronasal input (CH‐R). (B) Correlation between frontal alpha asymmetry (FAA) and frontal‐midline theta power in CH‐R. (C) Temporal dynamics of FAA during 60 s stimulus period for 8, 10, and 12 min samples under CH‐R. (D) Spatiotemporal scalp topographies across sequential 10 s windows of the 60 s stimulus period for CH‐R with different roasting times. (E) Averaged PSD curves and corresponding scalp topographies for *δ*, *θ*, *α*, *β*, and *γ* bands, showing frequency‐specific power distribution and cortical localization. (F) Summarized PSD‐AUC across five functional brain regions (frontal, parietal‐occipital, left temporal, central, right temporal). (G–K) SHAP‐based interpretable analysis under CH‐R: (G) channel importance ranking; (H) temporal waterfall plot of SHAP values; (I) summary bar plot of channel contributions; (J) spatiotemporal SHAP heatmap; (K) channel‐level SHAP scatter plot quantifying positive (red) and negative (blue) contributions to isolated retronasal perception.

**Figure:**
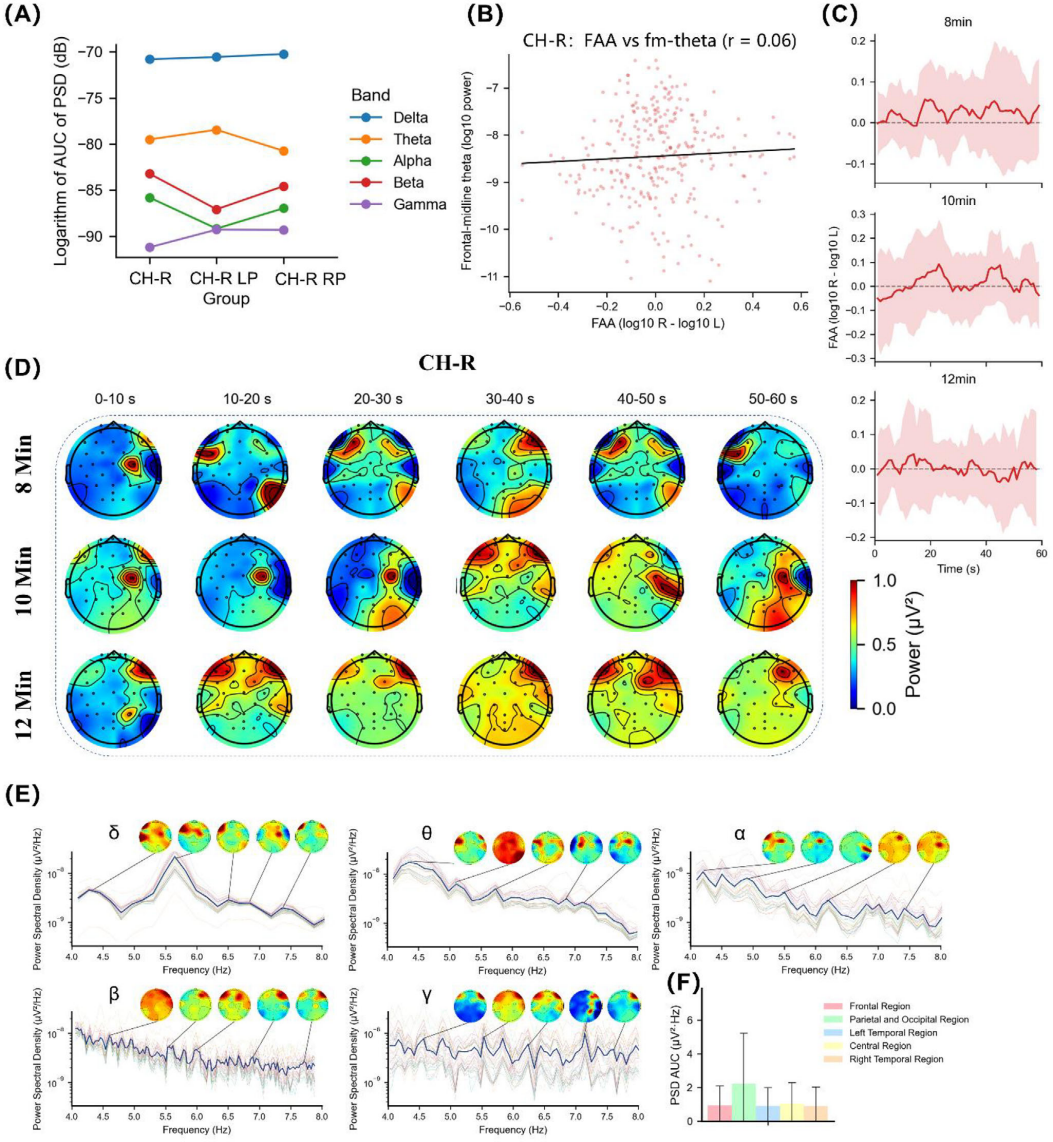

**Figure:**
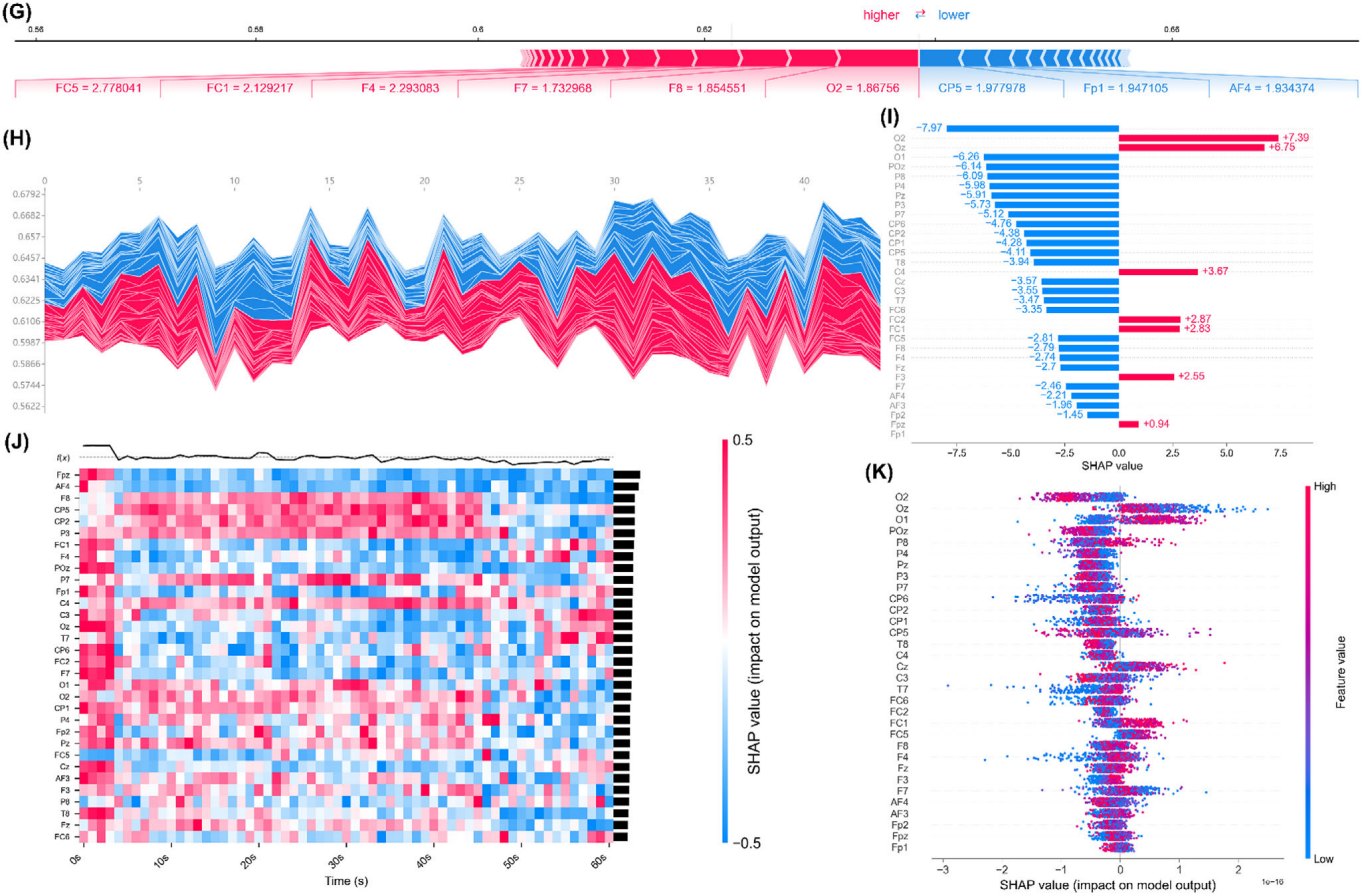

EEG topographic analysis showed that brain power distribution under isolated retronasal aroma input (CH‐R) changed regularly with grilling time (Figure 5D). The 8 min sample exhibited a power distribution pattern with high dispersion and strong spatiotemporal variability. As grilling time extended to 10 and 12 min, power distribution gradually concentrated in the frontal and central regions, and the neural encoding pattern became increasingly stable. This pattern was consistent with observations under normal eating conditions, where the CH‐NC group showed high stability and the CH‐C group displayed large variability. This consistency indicates that sustained retronasal aroma input is a core factor maintaining the stability of central neural encoding of flavor perception. Full‐band power spectral features and summarized regional AUC results further confirmed this trend (Figure 5E,F). Under isolated retronasal aroma input, *θ–α* band power in the frontal and central regions made the largest contribution to differentiating samples with different grilling times.

It should be noted that topographies reflect the spatial distribution of electrical potentials on the scalp surface. Affected by volume conduction effects, they cannot be directly equated with activation of specific brain regions. To further verify the specificity of retronasal aroma‑evoked neural responses at the anatomical level and provide spatial localization support for the scalp‑level findings above, supplementary sLORETA‑based EEG source localization was performed (Figure S6). The figure presents the spatial distribution, correlation‑based connectivity, and energy distribution of EEG responses under different experimental conditions. The top panel shows the response distribution for the 8, 10, and 12 min samples in the left and right hemispheres, with red areas indicating increased EEG responses. Under the CH‑R condition (pure retronasal response obtained by voxel‑wise subtraction of CH‑C from CH‑NC), the responses were mainly concentrated in the prefrontal, medial frontal, and temporal regions, suggesting that retronasal aroma‑related signals have a relatively focused cortical representation. The middle panel displays correlation‑based connectivity maps integrated across the three grilling times. In the CH‑R condition, connectivity was primarily distributed in the prefrontal, medial frontal, cingulate, and temporal regions, indicating that retronasal aroma‑related EEG signals tend to form a connectivity pattern dominated by prefrontal‑medial networks. The bottom panel summarizes the estimated cortical energy distribution. Under CH‑R, high‑energy regions were mainly located in the prefrontal, medial frontal, and cingulate areas, accompanied by partial temporal energy, suggesting that retronasal aroma‑related signals mainly engage neural activities involved in olfactory identification, attentional modulation, and hedonic processing. Notably, activation in the central sensorimotor and parietal regions was almost eliminated under the CH‑R condition, confirming that the subtraction design effectively removed interference signals from masticatory movements and somatosensory inputs.

Based on the combined analysis of scalp topography and source localization, the frontocentral power concentration observed at the scalp level mainly corresponds to activation of the prefrontal, medial frontal, and cingulate regions in source space. These regions are core cortical nodes for olfactory processing, flavor reward encoding, and attentional‐emotional modulation. Collectively, the electrophysiological evidence from this study demonstrates that retronasal aroma engages an olfactory‐reward‐attention cortical network, with activation intensity showing a dose‐dependent relationship with perceived aroma intensity.

**FIGURE 6:**
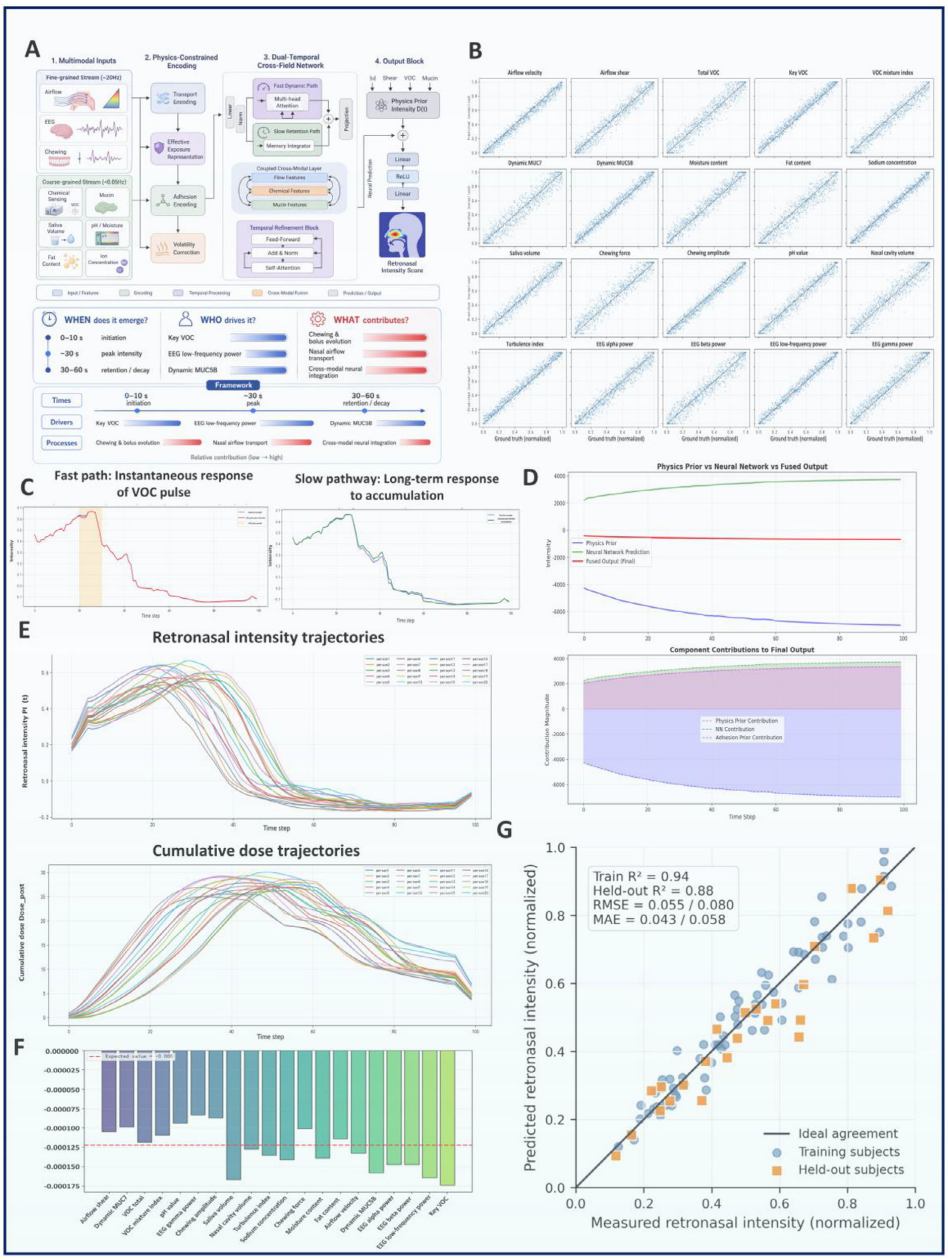
A Physics‐guided dual‐temporal cross‐field network (PG‐DTCFN) for mechanistic decoding and high‐precision prediction of dynamic retronasal aroma perception. (A) Overall architecture of the proposed physics‐guided dual‐temporal cross‐field network (PG‐DTCFN), including the multimodal input streams, physics‐constrained feature encoding modules, core dual‐temporal cross‐field network structure, and the complete calculation process of the final retronasal aroma perception output. (B) Scatter plots of normalized model‐predicted values versus normalized ground truth values for all core input features, to present the fitting performance of the PG‐DTCFN model. (C) Temporal response curves of the model's fast dynamic path and slow retention path, to present the dual‐temporal response characteristics of the model corresponding to instantaneous VOC release and long‐term aroma retention. (D) Temporal trajectories of the physics prior module output, pure neural network prediction, and final fused model output, as well as the contribution decomposition of each physics prior component to the final model output, to present the multi‐source information fusion mechanism of the model. (E) Predicted dynamic trajectories of retronasal aroma perception intensity and cumulative stimulus dose for all samples, to present the dynamic change process of retronasal aroma perception during oral processing reproduced by the model. (F) Global sensitivity analysis results of the PG‐DTCFN model, to present the relative importance and impact degree of each input feature on the final model output of retronasal aroma perception intensity. (G) Training and held‐out prediction agreement of the PG‐DTCFN model. The scatter plot shows measured versus predicted normalized retronasal intensity for the training subjects and held‐out subjects, with the diagonal line indicating ideal agreement.

SHAP interpretable analysis identified the core regulatory channels for isolated retronasal aroma perception and clarified the differences in neural encoding compared to the CH‐NC condition. Figure 5G shows that channels such as FC5, FC1, F7, F8, and O2 had the highest *F*‐values, marking them as core channels for distinguishing retronasal aromas from different grilling times. Among these, the prefrontal channels (F7, F8) showed high overlap with the core regulatory channels under the CH‐NC condition, confirming the prefrontal cortex as a core target for retronasal aroma encoding. Analysis of the temporal distribution of SHAP values showed that the positive contribution of these core channels to retronasal aroma persisted throughout the entire 60‐s sniffing cycle, peaking during the mid‐sniffing period (15–45 s) (Figure 5H). Prefrontal channels (Fp1, AF3, F7) made the largest positive contribution to the model output (Figure 5I). This pattern differs significantly from the high‐contribution pattern of parietal and occipital channels observed under the CH‐NC condition. This indicates that the basic perceptual encoding of isolated retronasal aroma relies primarily on the prefrontal olfactory and reward processing pathways. In contrast, the multisensory integration of retronasal aroma with taste and chewing further activates cross‐modal integration areas in the parietal and occipital lobes, forming a broader and more stable neural encoding network. Subsequently, the SHAP heatmap and beeswarm plot (Figure 6J,K) verified that under isolated aroma input, the feature contribution pattern of the core channels was highly concentrated. High‐contribution channels were all focused on the prefrontal region, and this concentration increased further as perception time progressed. This result is completely consistent with the spatiotemporal stability observed in the brain topographic maps.

#### 2.6.3 Individual‐Level Validation of EEG Findings

To further improve transparency and reproducibility, we added a subject level PSD analysis. This analysis aimed to verify the modulatory effect of retronasal aroma on low frequency EEG oscillations at the individual level. All supplementary results were aggregated from single subject data of 20 participants. This preserved inter individual variation. Therefore, we avoided the risk that group averaging might mask individual differences. Notably, all 20 subjects were trained sensory panelists. Their EEG data showed high consistency and interpretability. Presenting the distribution of individual level data demonstrates our commitment to methodological transparency. It also provides a reusable phenomenological basis for future studies.

Figure S7 first shows the average and individual distributions of PSD area under the curve across frequency bands. These bands were from frontal, temporal, central, and parieto occipital regions under CH‐C and CH‐NC conditions. Figure S7A shows that under the CH‐NC condition, the mean power of delta and theta bands in frontal and central regions was systematically higher than that under the CH‐C condition. This difference occurred at three grilling times (8, 10, and 12 min). Moreover, the difference progressively amplified with longer grilling duration. The group differences in temporal and parieto occipital regions were relatively milder. As shown in Figure S7B, despite inter individual physiological variability in baseline power levels across the 20 subjects, the direction of low frequency power increase induced by retronasal aroma was consistent across most subjects. No systematic opposite results appeared. This indicates that the effect had stable inter subject consistency and was not driven by a few extreme individuals.

A subject by brain region PSD difference heatmap further verified the spatial distribution of the effect (Figure S8). This figure used peak PSD difference as an index. It directly showed the response magnitude in each brain region for each subject. Results revealed a clear fronto central dominance in the spatial distribution of power enhancement induced by retronasal aroma (Figure S8A). At all grilling durations, the difference values in frontal and central regions were the highest. Moreover, the spatial distribution pattern of differences was highly similar across the 20 subjects, without obvious spatial dispersion (Figure S8B). These results echo the group level analysis from the main text at the individual level. They provide finer grained data supporting the stability of the finding that fronto central low frequency oscillations are a core neural marker of retronasal aroma perception.

Supplementary analysis of peak frequency distribution further corroborated the above conclusions from the frequency dimension (Figure S9). Compared with the CH‐C condition, the peak frequency of power spectra in frontal and central regions shifted toward lower frequency bands under the CH‐NC condition. This shift concentrated more in the delta and theta range. Under the CH‐R condition, the peak frequency also showed a trend toward lower frequencies. The magnitude of shift was between that of CH‐C and CH‐NC. Temporal and parieto occipital regions showed no significant peak frequency shift across the three conditions. This suggests that retronasal aroma does not globally change whole brain oscillatory features. Instead, it specifically modulates low frequency oscillations related to reward and attention processing. This mutually reinforces the conclusions from the previous frequency band analysis. As shown in Figure S9B, using single subject PSD curves at 12 min grilling time as an example, the individual level results further verified the robustness of the above group pattern. At the same time, they clearly presented natural physiological heterogeneity among subjects. Regarding effect consistency, all 20 subjects showed a highly uniform direction of change in frontal and central regions. This indicates that the low frequency shift effect in fronto central regions has stable inter subject consistency and is not driven by extreme individuals. Regarding individual differences, clear physiological variability existed across subjects in baseline power amplitude and absolute peak frequency. Some subjects had higher overall PSD baseline levels and stronger low frequency background. Some subjects had lower baseline power, but the relative offset magnitude induced by retronasal aroma remained clearly discernible. Inter individual differences in temporal and parieto occipital regions were more pronounced. The peak frequency positions were more dispersed and did not show a consistent shift trend. This further supports the brain region specificity of the frequency shift effect.

In summary, the subject level spectral and functional connectivity analyses together indicate that the modulatory effect of retronasal aroma on fronto central low frequency oscillations and related brain networks has clear condition specificity and good inter subject consistency. These individual level supplementary data provide finer grained support for the group level conclusions of the main text. All results are exploratory association evidence. They aim to objectively present the EEG response patterns corresponding to retronasal aroma perception. The fully open distribution of individual data further enhances the transparency and reusability of this study.

### 2.7 Analysis of Retronasal Aroma Perception Patterns in Grilled Lamb Skewers Using the PG‐DTCFN Framework

Building on the systematically elucidated regulatory mechanisms of retronasal aroma perception in grilled lamb skewers, the developed PG‐DTCFN framework achieved high‐accuracy, highly interpretable, and strongly generalizable prediction of retronasal aroma intensity. The results demonstrated that the PG‐DTCFN's full‐variable predictions of retronasal intensity showed high consistency with the actual measured values, exhibiting excellent overall fitting accuracy and operational stability. The scatter plot of predicted versus actual values for all input variables in Figure 6A shows that the model's predictions closely followed the ideal diagonal agreement, with no significant systematic bias or outliers. This confirms the model's precise fitting capability for the multi‐dimensional physical, physiological, and neural variables involved in this study. Additionally, to assess the model's stability under different operational conditions, the data were stratified for analysis based on airflow velocity (low/medium/high) and MUC5B mucin level (low/medium/high). Model prediction performance metrics were calculated for each subset. As shown in Tables S9 and S10, the performance metrics of each group tend to be consistent (with individual generalization ≤ 0.019, Δ*RMSE* ≤ 0.020, and Δ*MAE* ≤ 0.016 for both stratification types), indicating that the model exhibits stratified stability within the current experimental scope. This result aligns with the design logic of the physical constraint term in PG‐DTCFN, supporting the role of physical priors in reducing stratification dependence and improving prediction consistency.

Validation of the functional separation of the dual‐timescale paths demonstrated that the model successfully achieved differentiated and precise modeling of the two dynamic processes in retronasal aroma perception. This aligns closely with the aroma release‐perception mechanism revealed in this study. The path response curves in Figure 6B show a clear mechanistic division of labor between the fast dynamic path and the slow retention path. The fast path exhibited a sharp, transient response to VOC pulses, with its rise and fall rates perfectly synchronized with the aroma pulses. This accurately matches the sub‐second dynamics of nasal gas transport and aroma release during chewing, corresponding completely to the TI result where retronasal aroma intensity peaks rapidly during mid‐mastication. The activation intensity change of the slow path was smoother, more lagged, and of longer duration. It completely characterized the long‐term cumulative effect of aroma molecule adsorption and retention mediated by MUC5B mucin, directly echoing the earlier finding that chewing‐induced increases in salivary MUC5B prolong the duration of retronasal aroma perception. This result confirms that the proposed dual‐path architecture successfully achieved differentiated modeling of the rapid transport kinetics and slow adhesion/retention processes of retronasal aroma, without degenerating into a single timescale model. It thus algorithmically reconstructs the core dynamic features of retronasal aroma perception during eating.

Validation of the physics residual correction module indicates that the PG‐DTCFN is a hybrid model with clear physical meaning, not an unconstrained black‐box fit. Figure 6C compares the temporal evolution of the physics prior intensity, the neural network‐predicted intensity, and the final fused output. The physics prior provided a stable baseline for retronasal intensity that conforms to fluid transport theory. The neural network accurately captured the dynamic residual fluctuations caused by chewing, salivary physiology, and individual differences. The final fused output completely reconstructed the true dynamic characteristics of retronasal aroma intensity. The contribution proportion of the components further revealed that the physics prior provided the core baseline support for the model output. The neural network term and the adhesion prior term supplemented the detailed contributions from physiological regulation and individual specificity. The contribution proportion of these three components changed dynamically with the chewing process. This perfectly matches the retronasal aroma perception patterns we revealed. This result proves that the model did not discard the physics prior entirely. Instead, it performed residual correction via the neural network on top of a physically reasonable baseline. This approach ensured both the physical plausibility of predictions and the ability to fit complex biological processes.

Furthermore, the PG‐DTCFN effectively captured the differences in retronasal perception responses among different subjects, demonstrating excellent individual generalization performance. The predicted retronasal intensity curves for different subjects in Figure 6D show significant individual specificity in the model's output regarding peak intensity, rise, and fall time evolution shapes, rather than using a uniform template. This accurately reconstructed the differences in retronasal perception arising from variations in chewing behavior, salivary secretion characteristics, and neural response thresholds among subjects. The corresponding cumulative dose curves further confirmed that, while depicting individual differences, the model always maintained the physical plausibility of a monotonically increasing dose over time. No anomalous outputs violating the basic laws of aroma transport were observed. The model thus achieved a unification of personalized prediction and physical constraints, addressing the engineering challenge of poor generalizability and inability to adapt to individual eating differences in traditional models.

The results of the global sensitivity analysis (GSA) further validated the mechanistic interpretability of the model. The contribution of each input variable was highly consistent with the experimental conclusions and theoretical expectations of this study. The distribution of first‐order Sobol sensitivity indices in Figure 6E shows that core driver variables, such as key VOCs content, EEG low‐frequency power, and dynamic MUC5B content, had the most prominent contributions. Among these, the dominant role of key VOCs completely aligns with the TDS/TI result that retronasal aroma is the core of flavor perception. The high contribution of EEG low‐frequency power directly validates the earlier discovery that *δ/θ* low‐frequency oscillations are the core neural markers for central encoding of retronasal aroma. Physiological/environmental variables like dynamic MUC7, water content, sodium ion concentration, and airflow velocity showed moderate sensitivity. This finding cross‐validates the revealed roles of salivary mucins, taste‐aroma synergy, and the regulatory effect of nasal airflow on aroma transport. Variables such as chewing force, chewing amplitude, and fat content showed stable and reliable contributions. This perfectly matches the logic that the grilling process alters chewing behavior by modulating sample texture and physicochemical properties, thereby affecting aroma release. The contribution of all variables showed no mechanistic deviation. This supports the model's interpretability and physical consistency, demonstrating that it is not merely a data fit but reflects the underlying serial physical, physiological, and neural regulatory mechanisms of retronasal aroma perception.

Figure 6G further demonstrates the predictive performance of PG‐DTCFN on both the training set and the held‐out subject test set. The scatter plot of predicted versus actual retronasal aroma intensity showed that the training set achieved an *R* ^2^ of 0.94, RMSE of 0.055, and MAE of 0.043, indicating excellent fitting accuracy. More importantly, the held‐out subject test set (participants not seen during training) maintained a high *R* ^2^ of 0.88, with RMSE of 0.080 and MAE of 0.067. The slightly lower but still robust performance on the test set demonstrated that the model generalized well to new individuals without retraining. Notably, the scatter distribution revealed non‐uniform intensity coverage typical of real sensory data, and the test‐set errors were modestly larger than training‐set errors—consistent with genuine generalization rather than overfitting. These results confirmed that the PG‐DTCFN's physics‐guided architecture, which incorporated physical priors and dual‐timescale separation, effectively reduced overfitting and enabled reliable prediction of retronasal aroma intensity across individuals. Compared to purely data‐driven deep learning models that would require substantially larger sample sizes to achieve comparable generalization, the PG‐DTCFN achieved strong out‐of‐sample prediction with only 20 trained panelists, highlighting its data efficiency and methodological suitability for sensory neuroscience applications.

### 2.8 Simulation of Retronasal Aroma Perception

**FIGURE 7:**
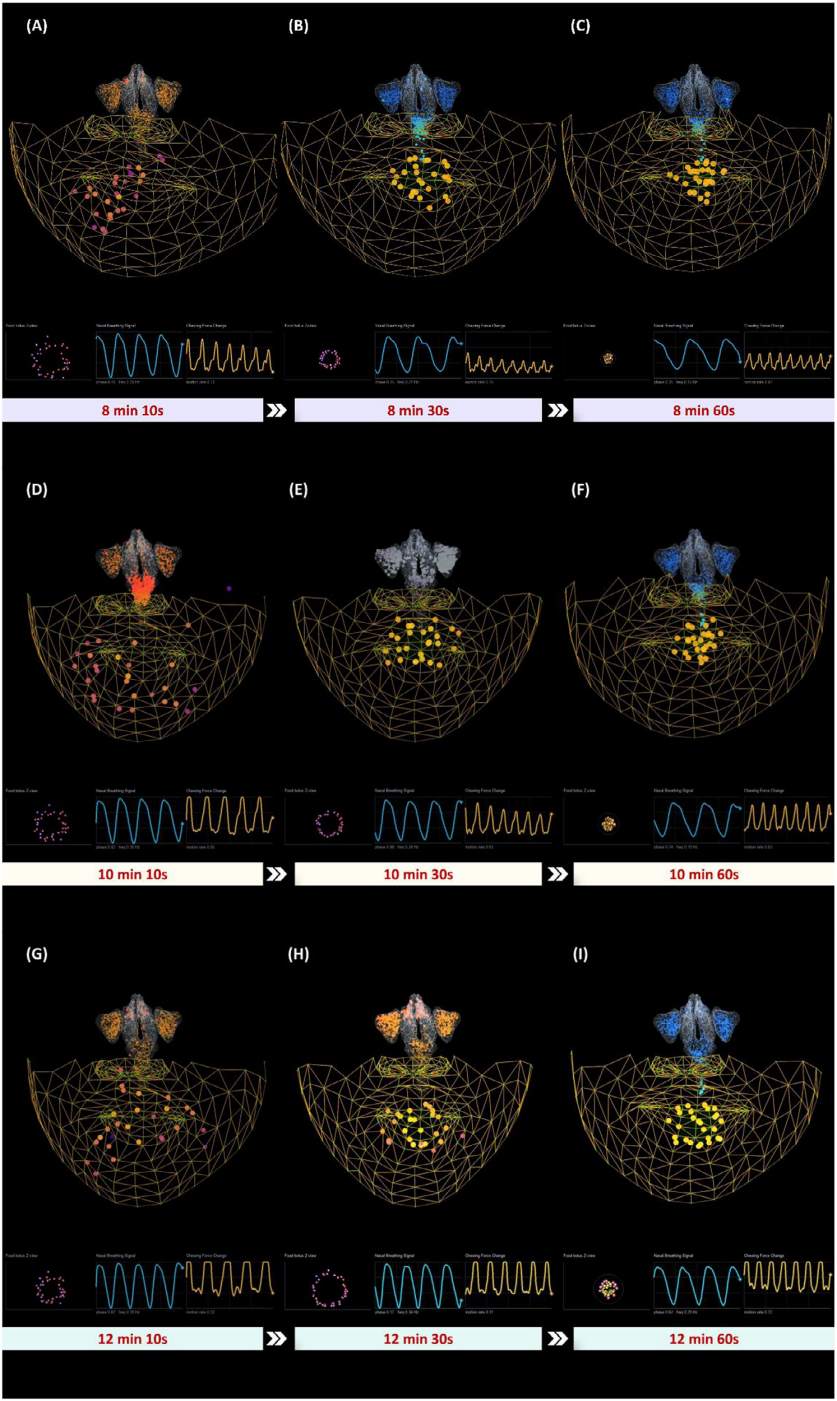
Key time frames of the multi‐physics coupled simulation of mastication, bolus evolution and retronasal aroma transport. (A–C) 8‐min grilled sample at 10, 30, and 60 s of mastication; (D–F) 10‐min grilled sample at 10, 30, and 60 s; (G–I) 12‐min grilled sample at 10, 30, and 60 s. Each panel integrates the mandibular masticatory mesh, oral bolus particle distribution, 3D nasal aroma particle distribution, and synchronized nasal breathing waveform and chewing force curve. The Z‐view insect shows the top–down distribution of bolus particles at each time point.

Based on the experimentally measured data for chewing behavior, bolus texture properties, nasal airflow dynamics, and dynamic retronasal aroma perception, the constructed multi‐physics integrated simulation framework coupling chewing mechanics, bolus evolution, and nasal respiration was fully validated. To visually demonstrate the spatiotemporal coupling between bolus breakdown and retronasal aroma transport, key frames at 10, 30, and 60 s of mastication were extracted from the full dynamic simulations for all three grilling durations, as summarized in Figure 7. Each panel synchronously displays the triangular mesh of the mandible representing masticatory mechanics, the particle‐based bolus structure in the oral cavity, the 3D spatial distribution of aroma particles within the nasal cavity, and the corresponding real‐time nasal breathing waveform and chewing force dynamic curve. Full dynamic animations are provided as Videos S1–S3. The simulated frames showed high consistency with the experimentally obtained texture characteristics, chewing mechanics, and retronasal aroma release patterns, fully confirming the reliability and predictive accuracy of the simulation model.

Regarding the dynamic evolution of bolus fragmentation and homogenization, the time‐series frames clearly reveal a grilling‐time‐dependent breakdown trajectory across the chewing cycle. For the 8‐min sample (Figure 7A–C), bolus particles remained relatively large at 10 s, and the homogenization process proceeded slowly throughout the 60‐s period, with chewing force maintaining a low and stable amplitude. The 10‐min sample (Figure 7D–F) showed a moderate rate of bolus fragmentation and homogenization, with particle size notably reduced by 30 s and chewing force fluctuating at an intermediate level. The 12‐min sample (Figure 7G–I) achieved extensive fragmentation early in the chewing phase: at 10 s, a larger number of fine particles had already been generated; at 30 s, particle size reached its minimum and total exposed surface area peaked; by 60 s, particles had aggregated into a compact, cohesive bolus ready for swallowing. This progressive pattern matched perfectly with the measured texture hardness and dynamic chewing force data for samples with different grilling times. The core parameters of the particle‐spring model within the framework were dynamically calibrated using hardness and particle size curves measured by a texture analyzer, ensuring consistency between simulated bolus evolution and real oral processing.

Regarding the dynamic simulation of retronasal aroma release and nasal transport, the spatial distribution of aroma particles in the nasal cavity showed clear time dependence and grilling‐time dependence, synchronized with the respiratory waveform. At 10 s of mastication, aroma particles had just begun to enter the nasopharynx, with sparse distribution in the lower nasal cavity and minimal deposition in the olfactory cleft region. At 30 s, large quantities of aroma particles reached the olfactory epithelium, and particle density in the olfactory cleft reached its maximum, corresponding exactly to the peak of VOCs release detected by GC‐IMS and the peak of perceived intensity recorded by TI. By 60 s, aroma particle concentration in the nasal cavity gradually decreased as the release rate slowed. Across grilling durations, the 8‐min sample exhibited a gentle aroma particle release rate, and particle distribution in the olfactory epithelium region was sparse and short‐lived. For the 10‐min sample, the peak aroma release was concentrated in the mid‐chewing phase, and particle distribution within the nasal cavity was sustained and stable. The 12‐min sample released a large quantity of aroma particles early in chewing, and throughout the entire cycle, particle distribution in the olfactory epithelium region was the densest and longest‐lasting. This dynamic characteristic completely aligned with the grilling time‐dependent patterns of retronasal aroma intensity timing, peak occurrence, and duration obtained from the TDS and TI experiments. Furthermore, the simulated nasal airflow field and aroma particle transport paths were linearly calibrated using measured flow velocity data from a nasal anemometer, with inlet flow velocity error controlled within 10%, further verifying the accuracy of the nasal flow field modeling.

Notably, the synchronized chewing force and nasal breathing curves reveal a kinetic coupling between masticatory behavior and respiratory patterns. As grilling time increased and sample hardness rose, the amplitude of chewing force increased significantly, which in turn drove a compensatory increase in respiratory frequency and tidal volume. Higher airflow velocity accelerated the transport of aroma molecules from the oral cavity to the olfactory cleft, directly elevating collision intensity between aroma molecules and the olfactory mucosa.

Overall, for every step of the full‐process dynamic results from this simulation framework—from chewing mechanics driving bolus structural evolution to retronasal aroma transport—parameters and boundary conditions were set based on the physiological, physical, and sensory experimental data measured in this study. The simulated frame results for different grilling times and different chewing time points all showed high consistency with the prior experimental conclusions, with no mechanistic deviation. The added time‐series visualization explicitly demonstrates the dynamic coupling law between bolus particle evolution and retronasal aroma particle transport, greatly enhancing the mechanistic interpretability of the model. This fully demonstrates the reliability, accuracy, and mechanistic interpretability of this multi‐physics integrated simulation model and provides a reliable numerical simulation tool for analyzing the dynamic process of retronasal aroma perception in grilled lamb skewers.

## 3 Discussion

### 3.1 Regulation of Bolus Physicochemical Properties by Grilling Time Determines the Kinetic Characteristics of Aroma Release

The primary prerequisite for generating retronasal aroma is the efficient release of aroma molecules from the food bolus into the gaseous phase within the oral cavity. The grilling time of lamb skewers significantly influences this release process. Fundamentally, different grilling times lead to variations in the texture and chemical composition of the meat. These differences, in turn, determine the patterns of chewing behavior and bolus evolution from the outset.

Longer grilling time causes muscle fiber contraction and protein denaturation, significantly increasing the initial hardness of the lamb (Figure 2F). Harder samples require greater bite force to achieve fragmentation during chewing. The oral behavior analysis in this study confirmed this inference. The peak chewing force and the mean force throughout the cycle for the 12‐min sample were significantly higher than those for the 8‐min and 10‐min samples (Figure 3). Chewing amplitude and fluctuation frequency were also higher. This phenomenon aligns with the fundamental principle of oral food processing, where food hardness is positively correlated with chewing force. More importantly, the higher chewing force translated directly into more efficient mechanical breakdown of the bolus. Bolus microstructure analysis showed that the 12‐min sample exhibited finer particle distribution and greater particle size variability in the late chewing stage (Figure 2I–M). This indicates a higher degree of fragmentation and more complete homogenization. The increased level of bolus fragmentation created three key conditions that further favor aroma release. First, mechanical breakdown increases the specific surface area of the bolus, reducing the mass transfer resistance for aroma molecules diffusing from the interior to the surface. Second, more thorough fragmentation promotes mixing between the bolus and saliva. This study observed the highest saliva content in the 12‐min sample during chewing (Figure 2C), consistent with the mechanisms where high chewing force stimulates saliva secretion, and the denser muscle fiber network more readily adsorbs saliva. As a medium for aroma release, the aqueous phase of saliva can dissolve polar aroma precursors. Enzymes present in saliva can catalyze the hydrolysis and oxidation of these precursors, promoting the generation of aroma molecules [5]. Third, more complete fragmentation accelerates the dissolution of Na^+^ from the bolus into the saliva phase (Figure 2E). As an ionic strength regulator, Na^+^ can alter the partition coefficient of aroma molecules at the air–liquid interface, promoting the release of hydrophobic aroma molecules into the gaseous phase [30].

This addresses the question of “where the aroma comes from.” However, aroma release is not instantaneous. The reason why the aroma perception of the 12‐min sample lasts longer requires further investigation. The answer lies in the chewing‐induced secretion of salivary mucins (Table S4). MUC5B and MUC7, core mucins in saliva, increased significantly during the chewing state. The mucin content was highest in the 12‐min sample during the late chewing stage. Mucins possess high adhesiveness and gel‐forming capacity. They can absorb aroma molecules, forming an “aroma reservoir” on the bolus surface [7]. This mechanism explains the phenomenon observed in the TI curves, where the 12‐min sample maintained relatively high retronasal aroma intensity even in the late chewing stage (Figure 1G–J). The mucin‐mediated adsorption–desorption process slows the aroma release rate, transforming an instantaneous pulse into sustained, slow release. This prolongs the duration of perception [19]. Concurrently, the increase in mucin content coincided with the rise in bolus cohesiveness and gumminess (Figure 2G,H). This indicates that while enhancing bolus structural stability, mucins also act as “carriers” for aroma molecules. This achieves a unification of structural function and flavor release function [31].

Therefore, a longer grilling time for lamb skewers promotes both the release intensity and the sustained duration of retronasal aroma through combined physical and chemical mechanisms. However, after their release from the bolus, aroma molecules must be transported through the nasal cavity to be perceived. This leads to the next question: how do these aroma molecules reach the olfactory epithelium?

### 3.2 Nasal Gas Flow Field and the Fluid Mechanics of Aroma‐Molecule Collisions: Constructing the Physical Pathway for Aroma Perception

Following their release in the oral cavity, aroma molecules must be transported through the nasopharynx to the olfactory epithelium to be recognized by olfactory receptors. This process is fundamentally a problem of molecular transport in a gas–liquid two‐phase flow, regulated by the combined effects of nasal anatomy, respiratory patterns, and the physical properties of the molecules themselves. Therefore, investigating how aroma molecules precisely reach the olfactory epithelium and why the transport efficiency is higher for the 12‐min sample warrants further exploration.

Nasal anatomical structure dictates the core spatial region for collisions between aroma molecules and the olfactory mucosa [32]. The results from the synchronously coupled high‐frequency particle flow field model show that, regardless of grilling time, the collision of aroma molecules with the olfactory mucosa was concentrated in the overlapping region of the mid‐to‐upper nasal cavity (*Z *≈ 20–25 mm) and the mid‐posterior segment (*X *≈ 25–40 px). This spatial localization corresponds closely to the anatomical position of the olfactory cleft. The olfactory cleft is a narrow channel at the roof of the nasal cavity, where the olfactory epithelium, the sole peripheral sensory site for olfactory perception, lines the mucosal surface [33]. Aerodynamic analysis indicates that this region exhibits the maximum airflow velocity gradient and moderate turbulence intensity. This ensures effective contact between aroma molecules and the mucosa while avoiding the “washing‐out” effect that excessively high flow velocities could cause. This finding, from a fluid dynamics perspective, explains how the nasal cavity naturally provides a preferential pathway for aroma molecules to reach the olfactory epithelium.

However, anatomy provides only a static foundation. The key factor determining whether aroma molecules effectively reach the olfactory epithelium is the dynamic respiratory pattern driven by chewing [34]. This study coupled nasal airflow simulation with chewing behavior, revealing that the respiratory pattern changes dynamically with chewing force. Due to its higher chewing force demand, the 12‐min sample showed a compensatory increase in respiratory rate and tidal volume early in the chewing phase. This physiological response was reflected in the simulation as an increase in nasal inlet flow velocity and enhanced airflow energy peaks. Furthermore, faster airflow velocity can shorten the transport time of aroma molecules from the mouth to the olfactory epithelium. However, it may also increase the risk of molecules depositing in non‐sensory areas due to inertial impaction [35]. The collision intensity data from this study show that the mean collision intensity for the 12‐min sample (1.128) was significantly higher than that for the 8‐min sample (0.220). This indicates that the probability of effective collisions between aroma molecules and the olfactory mucosa increases with higher airflow velocity. It suggests that, within the physiological range, increased airflow velocity primarily enhances the “targeted delivery” of aroma molecules rather than causing unnecessary loss.

Beyond the macroscopic airflow pattern, the physicochemical properties of the aroma molecules themselves determine their transport trajectories and deposition patterns within the nasal flow field [36]. The 48 volatile compounds detected by BreathSpec GC‐IMS encompassed various chemical classes, including aldehydes, ketones, alcohols, esters, pyrazines, and sulfur‐containing compounds. These have diverse molecular weights, polarities, and volatilities. Combined analysis of simulation and measurement in this study found that low‐molecular‐weight, highly volatile compounds were mainly distributed in the core airflow region, exhibiting high transport efficiency and short arrival times at the olfactory epithelium. In contrast, high‐molecular‐weight, less volatile compounds tended to deposit in the anterior nasal cavity, relying on turbulent diffusion to reach the olfactory epithelium. This distribution characteristic explains the temporal differences among various retronasal aroma attributes observed in the TDS. For instance, the Fatty retronasal aroma (aldehyde compounds) was perceived significantly earlier than the Grilled‐meat retronasal aroma (sulfur‐containing compounds). The latter requires longer transport time to accumulate to a dominant perception threshold.

The grilling time of the lamb skewers influences this transport process. Crucially, grilling time alters the volatile compound profile, thereby reshaping the collision intensity distribution within the nasal cavity. With increasing grilling time, the additional compounds in characteristic region C were mostly characteristic grilled‐lamb aroma components. These are more likely to remain in the gaseous state and penetrate the olfactory epithelium in the nasal flow field. Concurrently, the overall increase in VOCs abundance during the chewing of the 12‐min sample (Figure S3) means a greater total number of aroma molecules reach the olfactory epithelium per unit time. According to the dose‐response relationship, this translates directly into higher perceived intensity [37]. The TI curves, where the 12‐min sample showed the highest peak intensity and longest duration at all time points, are the external manifestation of this mechanism.

This addresses the question of how aroma molecules travel from the mouth to the olfactory epithelium. However, the physical collision of aroma molecules with the olfactory epithelium is merely the starting point of perception. The true flavor experience and emotional response depend on the cross‐modal integration of multisensory signals and the encoding and processing by the central nervous system. This leads to the third‐level question: how does retronasal aroma enhance taste perception and drive positive emotions?

### 3.3 Cross‐Modal Synergy and Central Neural Integration of Retronasal Aroma for Flavor Enhancement and Emotion Driving

The physical collision of aroma molecules with the olfactory epithelium is converted into olfactory signals that are transmitted to the central nervous system. However, processing olfactory signals alone does not produce the “flavor” experience. True flavor results from the obligatory integration of smell and taste at the central level. This necessitates clarifying the neural mechanisms by which retronasal aroma achieves cross‐modal synergy.

First, the cross‐modal enhancement of taste by retronasal aroma originates from the integration of olfactory and gustatory information in the brain. TDS results showed a high degree of overlap between the dominance periods of retronasal aroma attributes and basic tastes like saltiness and umami (Figure 1A,C). Furthermore, the nose‐clip control experiment confirmed that blocking retronasal aroma significantly reduced the perceived intensity of both umami and saltiness (Figure 1K–P). This phenomenon is not simply due to “attention allocation” or a “context effect.” Rather, it reflects the mandatory neural‐level integration of smell and taste. Olfactory signals from the olfactory bulb project to the orbitofrontal cortex via the piriform cortex [38], while taste signals project from the nucleus of the solitary tract to the insula and orbitofrontal cortex [39]. These two pathways converge and interact directly within the orbitofrontal cortex. Functionally, the orbitofrontal cortex is recognized as a core brain region for flavor perception. Its neurons respond more strongly to combined olfactory‐gustatory stimuli than to either modality alone [40]. In this study, the synergistic effect between retronasal aroma and tastes like saltiness and umami is the behavioral manifestation of this cross‐modal integration. The 12‐min sample, with the highest retronasal aroma intensity and longest duration, provided a more sufficient temporal window for cross‐modal integration. Consequently, it exhibited the most significant taste enhancement effect.

However, cross‑modal integration explains “flavor enhancement” but not entirely “emotion driving.” The reason why retronasal aroma elicits positive emotions remained unclear. EEG analysis revealed that this process involves the co‑activation of reward and emotional processing pathways. Low‑frequency bands (*δ* and *θ*) were identified as core neural markers for retronasal aroma input (Figure 4A). More importantly, the significant divergence of FAA between CH‑NC and CH‑C (Figure 4B) pointed to engagement of reward‑related processing by retronasal aroma. Source‑localized EEG further showed that CH‑NC evoked widespread cortical activation across the frontal, parietal, temporal, occipital, and cingulate regions, whereas CH‑C—despite identical chewing and taste input—produced only weak and spatially restricted activity (mainly in the frontal, temporal, and cingulate areas). This contrast provides direct spatial evidence that retronasal aroma is the primary driver of global cortical activation during food intake. At the electrophysiological level, the coupling between FAA and prefrontal midline θ power was significantly stronger in CH‑NC than in CH‑C (Fisher's *z*‑test, *p* < 0.05). Left‑lateralized FAA is a well‑established index of approach motivation and positive affect [25‑‐28]. Its enhanced coupling with *θ* oscillations therefore suggests that retronasal aroma contributes to the neural substrates of positive emotional responses during eating. It should be noted that the current EEG data (including source localization) primarily reflect cortical activity and cannot resolve deep subcortical structures such as the nucleus accumbens or quantify dopamine release. Nevertheless, the observed cortical activation patterns align with established flavor‑reward circuits [41, 42], providing direct electrophysiological evidence for the cortical mechanisms underlying retronasal aroma‑driven positive affect.

To further clarify the difference between the neural encoding of retronasal aroma itself and the neural encoding after multisensory integration, this study compared the EEG signals from the CH‐R condition with those from the CH‐NC condition. This comparison revealed the hierarchical structure of flavor experience formation. Furthermore, source localization of the CH‐R revealed that retronasal aromas specifically activated the prefrontal, medial frontal, cingulate, and temporal regions, with activation intensity increasing alongside grilling time. The prefrontal and medial frontal regions are key hubs for flavor integration and reward value encoding, receiving convergent projections from olfactory and gustatory pathways. The cingulate cortex is involved in attention allocation and emotional salience processing, and the temporal cortex corresponds to primary olfactory areas. SHAP analysis showed that under the isolated aroma condition, the core regulatory channels were concentrated in the prefrontal cortex (F7, F8) (Figure 5G). In contrast, under the CH‐NC condition, channels in the parietal and occipital lobes (O2, PO8) were additionally activated (Figure 4G). The prefrontal cortex is the core brain region for olfactory processing and reward encoding, responsible for basic aroma perception and pleasantness evaluation [14]. The parietal lobe is involved in multisensory integration and spatial attention orientation, while the occipital lobe is primarily associated with visual and tactile multimodal information integration. Isolated retronasal aroma requires mainly the prefrontal cortex for its perception and encoding. In the CH‐NC condition, the addition of taste, chewing, and tactile sensations requires the cross‐modal integration functions of the parietal and occipital lobes, forming a richer neural representation. Because the 12‐min sample had the most typical retronasal aroma characteristics and the highest perceived intensity, its isolated aroma input alone could drive significant prefrontal responses (Figure 5C). Under the multisensory condition, it further activated a broader network of brain regions (Figure 4D). This explains why the 12‐min sample not only had the highest perceived intensity but also elicited the strongest positive emotional response.

Based on the combined evidence from scalp EEG, source localization, and behavioral data, this study summarizes the process by which retronasal aroma enhances flavor and drives emotions into a three‐stage framework: the peripheral stage, where aroma molecules interact with the olfactory epithelium to generate olfactory signals; the central stage, where olfactory and gustatory signals undergo cross‐modal integration in brain regions such as the orbitofrontal cortex, forming a unified flavor representation; and the reward stage, where integrated signals engage the prefrontal‐anterior cingulate cortical reward network, promoting positive emotional experiences. This framework reveals that retronasal aroma is not only a core component of flavor perception but also a critical neural hub for cross‐modally enhancing taste perception and driving positive emotional responses.

### 3.4 Integration of Multiscale Findings Through Multiphysics Simulation and Deep Learning Models for Mechanistic Validation

The preceding mechanistic analyses at three levels—physicochemical, fluid dynamic, and neuroscientific—have revealed different facets of retronasal aroma perception. However, integrating these insights into a unified explanatory framework is necessary. The PG‐DTCFN deep learning model and the integrated mastication‐bolus‐nasal respiration multiphysics simulation framework constructed in this study provide complementary approaches. They offer reverse‐engineering and forward‐validation of the proposed mechanistic hypothesis from “data‐driven” and “first‐principles” perspectives, respectively. Together, they form a complete chain of evidence for the mechanistic investigation.

The PG‐DTCFN deep learning model, from a data‐driven angle, validates the core elements of the proposed multiscale mechanistic hypothesis. Through dual‐timescale path separation, the model successfully achieved differentiated modeling of the dual kinetic characteristics of retronasal aroma. The instantaneous response of the fast dynamic path to VOC pulses corresponds to the “mechanically driven rapid release” mechanism. The smooth modeling of the slow retention path for mucin‐mediated adsorption corresponds to the “mucin‐mediated sustained slow release” mechanism revealed in this study (Figure 6B). The model's successful differentiation of these two mechanisms, without degenerating into a single timescale, confirms that both play indispensable roles in retronasal aroma perception. Validation of the physics residual correction module further indicates that the model performs residual correction via a neural network on top of a physically plausible baseline (Figure 6C). This achieves a unification of physical laws and biological complexity. GSA further validated the importance ranking of various factors from the perspective of variable contribution. Key VOCs content, EEG low‐frequency power, and dynamic MUC5B content ranked as the top three contributors (Figure 6E). This finding cross‐validates the revealed principles, namely that aroma compounds are the material basis of perception, low‐frequency oscillations as core neural markers for encoding, and mucins as key regulators of sustained release. Notably, Na^+^ concentration and water content showed moderate sensitivity. This aligns with Na^+^ dual role in aroma release and taste perception and confirms the key role of saliva hydration in aroma release. This analysis quantifies the importance of ranking of factors from a data‐driven perspective. The absence of mechanistic deviation in the contribution of all variables demonstrates that the model is not merely a data fit. Instead, it accurately depicts the serial physical, physiological, and neural regulatory mechanisms of retronasal aroma perception.

The multiphysics integrated simulation framework, conversely, starts from first principles. It reproduces how these factors, through physical‐level interactions, ultimately affect perceptual output. As shown in Videos S1–S3 and Figure 7, the framework synchronously couples masticatory mechanics (Newton–Euler equations), bolus evolution (discrete element method), and nasal airflow (Navier–Stokes equations) with a 200 Hz timestep. The simulated frames for key time points (10, 30, and 60 s) for the 8‐, 10‐, and 12‐min grilling times showed high consistency with experimental observations. The 12‐min sample achieved complete fragmentation and homogenization early in chewing, with aroma particle distribution in the nasal olfactory epithelium region being the densest and longest‐lasting. The 8‐min sample showed larger bolus particles and sparser aroma particle distribution throughout the cycle. The significance of this simulation result lies not in simply replicating experimental data, but in performing forward deduction based on physical laws. It validates, at the first‐principles level, the physical self‐consistency of the chain: “grilling time affects chewing behavior by altering texture, which in turn regulates aroma release.”

Therefore, combining the findings from the deep learning model and the multiphysics simulation framework enables end‐to‐end simulation from processing conditions to perceptual output. The two approaches are complementary and mutually verifying, together constituting a complete evidence chain for the study of retronasal aroma perception mechanisms.

### 3.5 Research Contributions, Methodological Framework, and Limitations

#### 3.5.1 Theoretical Contribution: A Pioneering Multiscale Causal Framework

The research paradigm established in this study unifies mechanistic exploration and predictive modeling, opening a new path for understanding and a methodological framework for the study of retronasal aroma. Theoretically, this work is the first to construct a complete causal chain from oral processing to central perception, pioneering the investigation of the multiscale cascade mechanisms of retronasal aroma perception. Traditional research in this field has long been confined to analyzing individual steps, unable to answer the fundamental scientific question: “How is a food's matrix transformed, via oral processing behavior, into central nervous system signals?” This study breaks through this theoretical bottleneck. For the first time, it integrates macro‐level processing, meso‐level bolus evolution, micro‐level molecular transport, and central neural responses into a unified theoretical framework. It systematically elucidates the causal connections between physicochemical properties, fluid dynamic behavior, and neural encoding. The dual‐kinetics aroma release model proposed provides a universal theoretical tool for understanding release patterns of retronasal aroma across different food systems. At the neural mechanism level, the study is the first to reveal the hierarchical neural coding patterns between isolated retronasal perception and perception under multisensory eating conditions. It clarifies the three‐stage framework from peripheral input to reward‐associated cortical responses. This theoretical framework is not only applicable to grilled lamb skewers but can also be extended to other solid and semi‐solid foods. It offers a novel theoretical perspective for understanding the formation mechanisms of human flavor experience.

#### 3.5.2 Methodological Innovation: Establishing a Multiscale Integrated Research Paradigm

Methodologically, this study has pioneered a multiscale integrated research paradigm, providing a widely applicable methodological framework for the field of food science. This paradigm introduces computational fluid dynamics and 3D anatomical models into sensory research. It quantifies the nasal transport efficiency of aroma molecules from first principles, bridging the physical mechanism gap between aroma release and central perception. Building on this, the constructed PG‐DTCFN breaks new ground in deeply integrating physical priors with data‐driven modeling. It offers a new approach to solving the common technical challenge of multimodal data fusion. More importantly, the paradigm itself marks a shift from describing phenomena to modeling mechanisms, from a single dimension to multiscale integration, and from black‐box prediction to explainable intelligence. It provides a methodological model for future food sensory research. Subsequent researchers can adopt this paradigm to conduct similar multiscale integrated studies on different food systems and sensory modalities. This has the potential to propel the entire field of food sensory science from empirical research toward mechanistic research.

#### 3.5.3 Practical Implications: Informing Precision Regulation and Personalized Design

In terms of practical applications, this study provides a scientific basis and computational tools for the precise regulation and personalized design of food products, opening new paths for the healthy food industry. The quantitative relationships revealed between texture, chewing, and aroma release provide theoretical support for standardizing the processing of thermally processed meat products. The synergistic enhancement mechanism between retronasal aroma and tastes like saltiness and umami identifies new intervention targets for developing flavor compensation strategies for low‐sodium foods. This has the potential to overcome the long‐standing flavor‐loss bottleneck hindering the development of healthy foods. The developed deep learning model effectively captures individual differences, unifying personalized prediction with physical constraints. This lays the foundation for developing future personalized flavor modulation technologies and achieving precise nutritional interventions.

#### 3.5.4 Limitations

This study has several limitations. The research scope was constrained by the specificity and limited size of the sample pool. The investigation focused on a single food matrix (grilled lamb skewers), and the participants were a limited cohort of healthy young adults (*N* = 20). Although 20 professionally trained sensory panelists are sufficient for within ‐ subject, repeated ‐ measures EEG studies in food sensory science, the universality of the theoretical framework and the generalization capability of the PGDTCFN model to broader populations or other food matrices require further verification. Future studies with larger and more diverse cohorts and a wider range of food products are needed to test the generalizability of the proposed framework.

Moreover, the use of a nose clip to block retronasal pathways, while standard in the field, may introduce minor secondary effects on oral pressure and breathing patterns. However, our quantitative chewing force analysis confirmed that the nose clip did not significantly alter chewing kinematics in trained panelists, and the TI behavioral data independently supported that the observed taste enhancement was primarily due to retronasal blockade. Future studies employing alternative methods (e.g., reversed nasal airflow) could further exclude such mechanical confounds. Additionally, the GCIMS analysis of exhaled breath provided semiquantitative relative abundances rather than absolute concentrations. Future advances in realtime breath sampling and calibration may enable absolute quantification.

Furthermore, the neural mechanism interpretations—particularly the three ‐ stage framework and the involvement of reward ‐ related networks—are based on EEG and source localization. Scalp EEG has inherent spatial resolution limitations and cannot directly resolve deep subcortical structures or neurotransmitter release. Therefore, the proposed framework should be considered a hypothesis ‐ generating model rather than definitive mechanistic proof. Future studies employing higher ‐ resolution neuroimaging techniques (e.g., fMRI, simultaneous EEGfMRI) or interventional approaches (e.g., transcranial magnetic stimulation) will help validate and refine the causal pathways suggested here.

Despite these limitations, as a pioneering exploration into the multiscale mechanisms of retronasal olfaction, the core value of this work lies in establishing a complete theoretical framework from oral processing to central perception and demonstrating the feasibility of integrating food science, fluid dynamics, neuroscience, and deep learning. The significance of this work is not in pursuing statistically universal conclusions, but in providing a theoretical foundation and an experimental paradigm for subsequent research. Future studies can build upon this work to continuously refine and expand the framework.

## 4 Materials and Methods

### 4.1 Sample Preparation

Two hundred Sunit sheep with similar genetic backgrounds and standardized diets were selected from Xilingol League Meat Sheep Husbandry Co., Ltd. (Xilingol League, Inner Mongolia, China). The live weight of the sheep was 32.8 ± 2.5 kg. All animals were raised under identical conditions on a commercial farm, fed the same diet, and treated in accordance with animal welfare standards. From this group, 60 sheep were randomly selected for humane slaughter at a commercial abattoir. The slaughter and dressing procedures complied with Chinese national standards for meat production and food safety (GB/T 43562‐2023 “*Code of Practice for the Slaughtering of Livestock and Poultry—Sheep and Goats*”). *Longissimus dorsi* muscle samples were collected from each carcass immediately post‐slaughter. The meat was stored at 4°C for aging. Subsequently, the lean meat and sheep tail fat were frozen at −20°C, transported via a standardized cold chain to Hefei, Anhui Province, China, and stored at −20°C until use. Upon arrival, samples were thawed in a 4°C refrigerator for approximately 8 h until the core temperature reached −3°C to 5°C. After removing surface fat and connective tissue, the lean meat and fat were cut into uniform cubes of approximately 3 cm^3^ (2 × 1.5 × 1 cm). A total of 14 400 cubes were prepared, comprising 12 000 lean cubes and 2400 fat cubes. For each skewer, four lean cubes and one fat cube were threaded onto a 20‐cm‐long stainless‐steel skewer, resulting in a total weight of 25 g per skewer. The skewers were grilled over a charcoal fire at 220°C–230°C, positioned approximately 10 cm above the heat source. All samples were grilled in four batches, with each skewer rotated 180° every 30 s. Based on preliminary research [14], three grilling durations of 8, 10, and 12 min were selected for this study, corresponding to final core temperatures of 78°C, 80°C, and 83°C, respectively, as verified by a precision thermometer (YET610L, YOWEXA, Shenzhen, China). Two hundred skewers were produced for each grilling time per batch. Samples designated for instrumental analysis were vacuum‐packaged in nylon/polyethylene bags (Oxygen transmission rate: 9.3 mL O_2_/m^2^/24 h at 0°C; film thickness: 0.19 mm; MagicSeal, Guangdong, China) and stored at −80°C until analysis. This study involved only the physicochemical analysis and sensory evaluation of processed meat samples, with no scientific procedures performed on live animals. All grilled samples used for sensory evaluation and EEG experiments were equilibrated in a 50°C constant‐temperature water bath to reach a uniform core temperature before being presented to the evaluators. This procedure eliminated temperature variations caused by different baking durations, preventing interference from thermal effects and trigeminal nerve temperature stimulation on volatile release and sensory assessment.

A series of C4–C9 2‐ketones, including 2‐butanone (≥99.5%), 2‐pentanone (≥99.5%), 2‐hexanone (≥99.5%), 2‐heptanone (≥99.5%), 2‐octanone (≥99.5%), and 2‐nonanone (≥99.5%), were purchased from Aladdin Scientific Corp. (Shanghai, China). The following food‐grade aroma compounds were obtained from Anhui Jinlong Spice Co., Ltd.: 2,3,5‐trimethylpyrazine (98%), 3‐(methylthio)propionaldehyde (98%), furfural (95%), (E, E)‐2,4‐decadienal (99%), heptanal (98%), octanal (95%), nonanal (98%), (E)‐2‐heptenal (99%), 3‐hydroxy‐2‐butanone (97%), and guaiacol (99%).

### 4.2 Recruitment of the Sensory Panel

A total of 20 participants (10 male, 10 female) between the ages of 18 and 35 were recruited. All panelists were in good health, with a body mass index between 18 and 28 kg/m^2^. Additional selection criteria included: no reported oral health problems, chewing, or swallowing difficulties; normal gustatory and olfactory function; non‐smoker status; no history of relevant allergies; and not pregnant. Prior to the experiment, all participants received a detailed briefing on the study procedures and provided written informed consent. Monetary compensation was provided upon completion of the study. The protocol was approved by the Biomedical Ethics Committee of Hefei University of Technology [HFUT20260105002H]. All procedures comply with the Declaration of Helsinki.

### 4.3 TDS and TDE Method

#### 4.3.1 Panel Discussion and Training

To systematically evaluate bolus formation and retronasal olfactory perception during the oral processing of lamb skewers with different grilling durations, a comprehensive panel selection and training program was conducted. The training spanned one week and comprised ten sessions. During the initial session, panelists engaged in group discussions to generate and refine sensory terminology related to the oral processing of grilled lamb skewers. Through frequency analysis and collective deliberation, a final lexicon of 14 well‐defined sensory attributes, categorized into four dimensions, was established (Table S11). Furthermore, to assess the dynamic emotional changes of participants during mastication using the temporal dominance of emotions (TDE) method, the panel collaboratively defined 13 emotional attributes (Table S12).

In subsequent training sessions, panelists practiced with lamb skewer samples representing different grilling times. The principles of the TDS method were explained in detail. Based on the established lexicon, panelists were trained to identify and select the dominant sensation perceived over time, that is, the attribute capturing the most attention at a given moment, which was not necessarily the most intense [43]. The TDE method operated on a similar principle but required panelists to focus on the emotions elicited by the sample, specifically distinguishing between emotions directly evoked by the food (feelings/emotions as a rapid response to a stimulus) and general emotional states (moods as more enduring states in the absence of a specific stimulus).

Panelists then received practical training for the TDS and TDE test procedures using the SensoMaker software [44]. Upon completion of this training, the panel's performance was deemed satisfactory. A standardized chewing frequency of 1.4 chews per second for a duration of 60 s was established and practiced. The formal evaluations were conducted in four sessions over the course of one week.

#### 4.3.2 Formal Evaluation

During the formal evaluation, grilled lamb skewer samples were presented in random order. Each sample was served in a white plastic cup labeled with a three‐digit code. The TDS method was employed to dynamically characterize the sensory changes during a 60‐s mastication period. Upon placing a sample in their mouth, the panelist immediately clicked the “Start” button in the SensoMaker software. Throughout the evaluation, the panelist selected the dominant attribute perceived at that moment from the on‐screen list. A selected attribute was considered dominant until the panelist chose a different one. To prevent bias from fixed ordering, the sequence of attributes (and later, emotion terms for the TDE evaluation) presented on the screen was randomized for each panelist [45]. After 60 s of continuous evaluation, the panelist clicked the “Stop” button to end the data collection for that sample. Between evaluations, panelists rinsed their mouths with purified water, sniffed fresh air, and rested. The next evaluation began only after the panelist confirmed no residual aromas remained in the nasal cavity. All samples were evaluated in triplicate.

The TDE evaluation followed an identical experimental protocol and procedure, with the sole difference being the selection of emotional attributes instead of sensory attributes.

To identify the attributes or emotions that were truly dominant, the “chance level” and “significance level” for each sample were calculated. An attribute or emotion was considered dominant during a given period if the proportion of panelists selecting it exceeded the significance level. This calculation was performed using the formula proposed by Pineau et al. [43]:

(1) $$P_{S}=P_{0}+1.645\sqrt{\frac{P_{0}(1\;-\;P_{0}}{N}}$$

where *‘P_s_*’ is the minimum significant proportion value (*p* = 0.05) of the TDS/TDE curve at any time point; ‘*P_0_*’ is the chance level, calculated as ‘*P_0_ = 1/P*’ (where ‘*P’* is the number of attributes); *“N”* is the product of the number of group members and the number of repetitions.

### 4.4 Time‐Intensity (TI) Assessment

An interesting observation from preliminary training sessions indicated that the perception of saltiness and umami appeared to increase as chewing progressed, coinciding with greater aroma release—a phenomenon commonly described as the food becoming “more flavorful with chewing.” To investigate the dynamic changes in the intensity of retronasal aroma during oral processing and to verify its potential role in enhancing saltiness and umami perception, the TI method was subsequently employed. This evaluation was conducted with the same trained panel. Building upon their prior training, the panelists completed a separate, dedicated training and testing program focused on the TI methodology. This program consisted of eight sessions conducted over two weeks.

#### 4.4.1 Panel Training for TI Evaluation

A systematic training program was conducted to enable the panel to accurately assess the dynamic intensity of retronasal aroma and taste attributes. Training initially focused on enhancing the panelists' retronasal aroma perception. Over several weeks, panelists participated in four weekly sessions of oral rinsing exercises. These exercises aimed to familiarize them with the characteristics of ten different aroma compounds in aqueous solution. Based on their ability to correctly identify randomly presented compounds and judge their intensity, four key retronasal aroma attributes, along with their corresponding chemical references, were selected and defined: retronasal fatty aroma ((E,E)‐2,4‐decadienal), retronasal grilled lamb aroma (3‐(methylthio)propionaldehyde), retronasal smoky aroma (furfural), and retronasal grilled aroma(2,3,5‐trimethylpyrazine). For each attribute, a series of six solutions with increasing concentrations was prepared. Panelists practiced ranking these solutions correctly based on perceived intensity. A quantitative intensity scale from 1 to 10 was subsequently established. This scale adhered to the Weber–Fechner law, ensuring a logarithmic‐linear relationship between concentrations at adjacent scale points, which allowed for stable discrimination by the panelists. The finalized reference scales were: retronasal fatty aroma (0.01, 0.05, 0.5 mg/L (E,E)‐2,4‐decadienal corresponding to scores of 1, 5, and 10, respectively); retronasal grilled lamb aroma (0.02, 0.12, and 1 mg/L 3‐(methylthio)propionaldehyde); retronasal smoky aroma (5, 15, and 60 mg/L furfural); and retronasal *grilled aroma* (0.2, 1.2, and 6 mg/L 2,3,5‐trimethylpyrazine).

Taste training followed a similar approach. Panelists' taste sensitivity was assessed by their ability to distinguish between different concentrations of sodium chloride (NaCl) and monosodium glutamate (MSG) solutions. They then practiced ranking a series of six solutions, prepared via twofold serial dilutions from stock solutions (NaCl: 15 g/L; MSG: 20 g/L). After establishing a consistent concentration–intensity perception, reference points for the TI evaluation were defined. The scale anchors were set at 3, 5, and 9.20 g/L NaCl (corresponding to scores of 1, 5, and 10) for saltiness, and 0.5, 2.8, and 15 g/L MSG for umami.

Subsequently, panelists participated in simulated TI evaluation sessions, practicing continuous intensity scoring for the aroma and taste attributes. Their performance was evaluated based on the consistency of their repeated evaluation curves. To ensure the effectiveness of olfactory blockade in subsequent experiments, all panelists received standardized training on wearing a nose clip. They were instructed to fit the clip tightly over the bridge of the nose to achieve complete nasal occlusion, with the seal verified via an aroma recognition test. To minimize potential interference of the nose clip with mastication, panelists practiced chewing a flavorless matrix while wearing the clip until fully accustomed.

#### 4.4.2 Formal TI Evaluation

The formal evaluation was conducted in individual sensory booths. Lamb skewer samples (25 g each) representing the three grilling times were randomly coded and presented in white plastic cups labeled with three‐digit codes. At the start of each evaluation, the panelist rinsed their mouth and put on a nose clip. Immediately upon placing the sample in their mouth, the panelist removed the nose clip and simultaneously started the chewing action and the SensoMaker software. Over the following 60 s, the panelist performed a continuous TI evaluation for the four retronasal aroma attributes. A 10‐centimeter unstructured linear scale, anchored with “0 = none” and “10 = extremely strong,” was used on the screen for real‐time intensity recording. To verify the enhancing effect of retronasal aroma on taste perception, the evaluation of saltiness and umami was conducted under two control conditions. In the first condition, the nose clip was removed upon sample insertion, allowing retronasal aroma perception. In the second condition, the nose clip was worn throughout the entire evaluation, blocking retronasal olfaction. Between evaluations, panelists rinsed their mouths thoroughly and rested until they confirmed the absence of any residual aromas in the nasal cavity. All samples were evaluated in triplicate. Key parameters, including the time to maximum intensity (*T*_max_), the maximum intensity (Imax), and the AUC, were extracted from each TI curve for subsequent analysis.

### 4.5 Composition of Bolus

#### 4.5.1 Bolus Collection

The panelists described in Section 2.2 participated in five 120‐min bolus collection sessions. In each session, they masticated one type of lamb skewer (grilled for 8, 10, or 12 min). The order of sessions and skewer assignment was randomized. During each collection phase, panelists followed a predefined, standardized chewing protocol. They chewed a 25‐g lamb skewer at a fixed frequency of 1.4 chews per second. The bolus was expected to be a sealed plastic cup at precisely 10, 30, and 60 s of chewing. Panelists were not informed of the specific expectoration time points in advance; the timing for chewing and expectoration was cued by audio signals. After chewing three samples, panelists rested for 5 min and rinsed their mouths with warm water. A total of 9 boluses (3 replicates per sample) were collected for each lamb skewer type at each mastication time point (10, 30, and 60 s). Consequently, 180 boluses were collected per panelist per session. The collected boluses were subsequently used for texture profile analysis, determination of water and saliva content, and microstructural analysis.

#### 4.5.2 Moisture Content and Saliva Intake of Food Bolus

Bolus water content, which reflects the combined residual moisture from the grilled meat and the saliva incorporated during chewing, was determined gravimetrically. Expelled boluses collected at different chewing times (10, 30, and 60 s) were weighed (*B*_0_). They were then dried in an oven at 105°C for 16–18 h until a constant weight was achieved. After cooling, the dried boluses were weighed again (*B*_1_). The moisture content was calculated using the following Equation (2):

(2) $$B_{p}=\left(B_{0}-B_{1}\right)/B_{0}\times 100\%$$

In addition, the moisture content of the three types of grilled lamb skewers was measured as described above.

Subsequently, the saliva uptake of the food bolus was obtained and calculated using Equation (3):

(3) $$\begin{array}{c} Saliva\;uptake\;\left(\%\right)=\left[(b_{0}\;-\;b_{1}/b_{0}\right)\;-\;\left(w_{0}\;-\;w_{1}/w_{0}\right)]\;\times \;100\% \end{array}$$

where *b*_0_ is the weight of the food bolus after chewing, *b*_1_ is the weight of the food bolus after drying, *w*_0_ is the initial weight of the grilled lamb skewer, and w_1_ is the weight of the grilled lamb skewer after drying.

#### 4.5.3 Determination of Fat Content in Bolus

The fat content of three types of grilled lamb skewers was measured using the Soxhlet extraction method at chewing durations of 10, 30, and 60 s. The dried food bolus obtained after moisture determination (see Section 2.5.2) was ground into a homogeneous powder using a grinder (1500C, JINGESU, China). A portion of the ground dry bolus (approximately 1 g, denoted as *F_0_*) was extracted with ether using a Hanon Sox406 fat determination system (Hanon Future Technology Group Co., Ltd., China). After extraction, the ether was evaporated overnight to obtain the fat residue, which was then weighed (denoted as *F_1_*). The fat content of the bolus was calculated on a dry weight basis as follows:

(4) $$F_{c}=\left(F_{0}\;-\;F_{1}\right)/F_{0}\;\times \;100\%$$

#### 4.5.4 Bolus Texture Characteristics

The texture properties of food boluses from different samples and chewing durations were measured using a texture analyzer. The bolus was transferred from a sealed cup to a test cylinder (diameter 40 mm, height 30 mm) to a stacking height of 25 mm, and the surface was gently flattened using the back of a spoon. A P/6 probe was employed to compress the sample to 80% strain of its initial height at a constant speed of 5 mm/s, obtaining parameters such as hardness, cohesiveness, gumminess, and chewiness.

#### 4.5.5 Bolus Microstructure

The fragmented structure of the bolus was determined via image analysis. Approximately 25 g of each bolus was placed in a tray (20.3 × 30.5 × 5.1 cm). Subsequently, 250 mL of water was added to gently disperse the particles, followed by manual separation to ensure individual fragments. The sample was then placed in a black imaging chamber, and a background image was captured at a resolution of 600 DPI. Image analysis was performed using ImageJ software (National Institutes of Health, USA). The captured images were first converted to grayscale, and contrast was enhanced. The grayscale images were then binarized using either the Otsu method or an adaptive thresholding method. Morphological operations (opening and closing) were applied to remove small noise points and fill holes, thereby ensuring independent segmentation of particles. Individual particles were identified using connected component analysis or contour detection. From this, parameters including the number of particles (NumParticles), mean particle area (MeanArea), standard deviation of area (AreaStd), area ratio (AreaRatio), and mean circularity (MeanCircularity) were calculated.

#### 4.5.6 Determination of Sodium Ions in Bolus

To determine the sodium ion content, a 1 g sample of bolus (from each grilling time point) was digested. The sample was placed in a digestive tube with 3 mL of nitric acid and 1 mL of 30% hydrogen peroxide. The mixture was heated at 120°C for 1.5 h until it became a clear, colorless solution. Elemental analysis for sodium (Na) was performed using inductively coupled plasma optical emission spectrometry (ICP‐OES) (iCAP PRO X Duo, Thermo Fisher Scientific, USA). The instrument was operated under the following conditions: plasma gas flow rate of 12.5 L/min, auxiliary gas flow rate of 0.5 L/min, nebulizer gas flow rate of 0.5 L/min, and a plasma power of 1150 W. A calibration curve for sodium ions was established using standard solutions with concentrations of 0.1, 0.5, 2, 5, and 10 mg/L.

### 4.6 Physiological Parameters of Oral Processing

All panel members participated in the collection of physiological parameters related to oral processing. Informed consent was obtained from all participants, who were compensated for their involvement.

#### 4.6.1 Saliva Collection and Analysis

Saliva sampling was conducted between 8:30 AM and 11:00 AM. To standardize baseline physiological conditions, participants were instructed to maintain their normal sleep pattern and obtain sufficient sleep (≥7 h) the night before. Upon arrival, they verbally confirmed compliance. Participants were also required to abstain from alcohol and caffeinated beverages for at least 12 h prior, as these can influence saliva flow and composition. Upon arrival, participants brushed their teeth and confirmed they had fasted (consuming no food or beverages except water) for at least 2 h. After brushing and fasting, panelists rested for 30 min in the sensory laboratory (23°C–25°C, 50%–55% relative humidity) while drinking water. Unstimulated (resting) saliva and stimulated saliva (generated by chewing a flavorless chewing gum base) were then collected via a straw, yielding 3–5 mL each. The samples were centrifuged at 4°C and 10 000 rpm for 10 min, and the supernatant was collected. All experiments were performed in triplicate. The concentrations of mucins MUC5B and MUC7 were quantified using commercial ELISA kits (Cusabio Biotech Co., Ltd., China). Saliva pH was measured with a pH meter (BPH‐7100A/B/C, Bell Analytical Instruments Co., Ltd., Dalian, China). Sodium ion concentration in saliva was determined using the method described in Section 2.5.6.

#### 4.6.2 Facial Kinematics During Oral Processing

Facial movements of panelists chewing the three types of lamb skewers were recorded using an iPhone 15 Pro Max. Participants were instructed to face the camera directly and avoid obscuring their mouths with their hands. The recorded videos were subsequently analyzed using Open‐Source Computer Vision Library (OpenCV) to extract parameters such as chewing force and chewing amplitude.

First, extract the mandibular key point positions *p*(*t*) from the video and calculate the instantaneous mandibular velocity *v*(*t*):

(5) $$v\left(t\right)=\frac{dp\left(t\right)}{dt}\;$$

Velocity information was used to calculate the acceleration of jaw movement, which was then mapped to the force exerted by the masticatory muscles.

Subsequently, video‐driven chewing force estimation was performed. The jaw movement was mapped to the instantaneous bite force *F_c_* (*t*), and combined with the food bolus feedback force, simplified into a linear spring‐damper model:

(6) $$F_{c}\left(t\right)=k_{s}\Delta L\left(t\right)+c_{s}v\left(t\right)$$

where Δ*L*(*t*) represents the elongation of the bolus spring, *k_s_* and *c_s_* are the spring stiffness and damping coefficient, respectively, with values referenced from the particle‐spring bolus model. The estimated force was calibrated to the actual bite force range (300–700 N) through linear scaling, with the error controlled within 5%.

### 4.7 Nasal Airflow Measurement and the Synchronously Coupled High‐Frequency Particle Flow Field Model

To establish a geometric foundation for gas transport simulation, a three‐dimensional nasal cavity model representing a typical anatomical structure was first reconstructed. This study utilized the NasalSeg dataset, a repository of 130 clinical head CT scans with pixel‐level manual segmentation of the nasal cavity and paranasal sinus regions [46]. The annotated structures included the left/right nasal cavities, nasopharynx, and bilateral maxillary sinuses, covering the entire airway from the nasal vestibule to the posterior choanae and nasopharynx. Based on these annotations, the Marching Cubes algorithm was initially applied to extract a 3D surface mesh of the nasal region from each dataset. Subsequently, the individual models were spatially registered, scale‐normalized, and statistically fused to compute an average morphological model representing a typical nasal structure. Finally, mesh smoothing and topological repair were performed on the generated 3D model to enhance its geometric quality and structural integrity. The resulting model provided the anatomical basis for subsequent gas transport simulations.

To define dynamic boundary conditions for the simulation, real‐time respiratory data during mastication were acquired. An airflow sensor (HKH‐11Q, Hefei Huake Electronic Technology Research Institute, China) was used to monitor breathing intensity and frequency in real‐time as participants chewed lamb skewers grilled for 8, 10, and 12 min. The sensor, placed anterior to the nares, recorded respiratory waveforms via its proprietary software, quantifying the dynamic changes in nasal airflow during chewing.

Integrating the 3D nasal model and the measured respiratory data, a synchronously coupled high‐frequency particle flow field model was developed to simulate the global stimulation process of gases within the nasal cavity. This model balanced fluid dynamic fidelity with real‐time coupling efficiency and was structured around three physical mechanisms: gas transport (concentration–velocity flux), local dynamic stimulation (flow‐induced stimulation), and temporal exposure integration, as expressed in Equation (7):

(7) $$\begin{array}{ccc} D\left(t\right) & = & \int_{0}^{t}\int_{\Omega }C\;\left(x,\tau \right)V\left(x,\tau \right)(\alpha \left|\left|\nabla V\right|\right|\\ & & +\;\beta e^{-d/\lambda }+\gamma \left|V\right|V_{\max})d\Omega d\tau \end{array}$$

here, *C* (*x*,  τ) represents the gas concentration field, and *V* (*x*,  τ) denotes the airflow velocity field within the nasal cavity. The product *C* ×  *V* indicates the gas flux, reflecting the amount of gas transported into the sensory region per unit time. “*Ω*” represents the spatial region within the nasal cavity involved in gas perception or stimulation, such as the olfactory epithelium area or airflow stagnation zone. The parameters “*α, β, γ*” are dimensionless weighting coefficients used to control the relative contributions of shear effects, wall enhancement effects, and velocity amplitude effects in the overall stimulation model. This study refers to the method of Thomas et al. [47]. Based on the dominant role of airflow shear and wall interaction in nasal CFD research, the parameters were selected as *α* = 0.6, *β *= 0.3, *γ* = 0.1. Nasal simulations indicate that local airflow velocities can reach approximately 4 m/s, while wall shear stress typically ranges from 0.3 to 0.5 Pa. These magnitudes support the dominant role of the shear term in the stimulation mechanism. τ is the time variable in the integral, representing any intermediate time point from the initial moment to the current time *t*. In addition, the terms in parentheses in the formula describe the modulation of stimulus intensity by aerodynamics, which mainly consists of three main physical mechanisms. Among them, the velocity gradient term ||∇*V*|| represents the local airflow shear and particle inertial collision effects. The wall attenuation term *e* ^‐*d*/λ^ describes the stimulus enhancement effect generated when gas approaches the nasal mucosa; The velocity amplitude term |*V*|*V*_max_ characterizes the contribution of overall airflow drive to stimulus intensity. The outer time integral and spatial integral represent the cumulative processes over the respiratory cycle and the nasal sensory region, respectively, ultimately yielding the total stimulus dose “*D*(*t*)”. This metric can simultaneously reflect both instantaneous airflow disturbances and long‐term stimulation effects.

The synchronous coupled high‐frequency particle flow field model constructed in this section aimed to characterize the steady‐state features of nasal aroma transport during typical mastication, with a focus on quantifying the spatial distribution and intensity patterns of aroma molecule collisions with the olfactory mucosa (see Section 2.5 for details). This model complemented the dynamic simulation model (Model 3) in Section 4.11.3 in terms of research objectives: the former emphasized the analysis and visualization of spatial mechanisms, while the latter focused on the dynamic reproduction of the entire process along the time dimension. Both models employed the same nasal geometric model and respiratory boundary conditions but differed in their mathematical frameworks—the former was based on the macroscopic stimulus dose derived from concentration–velocity flux integration, whereas the latter solved transient flow fields using Navier–Stokes equations and tracked discrete particle trajectories. The Doppler velocity and collision‐intensity maps were displayed as normalized 0–1 indices to compare spatial patterns across samples. To avoid interpreting the normalized plots as absolute physical units, corresponding absolute inlet flow calibration values and representative velocity/wall shear stress magnitudes were provided subsequently.

To ensure reproducibility, the specific settings of the computational fluid dynamics‐particulate transport model were as follows. The nasal cavity geometry *Ω* was constructed based on an average model reconstructed from NasalSeg CT scans. The inlet boundary condition was driven by the measured masticatory breathing waveform u_in(*t*), while the downstream boundary was set as a pressure outlet. The nasal cavity walls were defined as no‐slip boundaries, with the olfactory mucosa/olfactory cleft designated as the target surface for aroma particle collision analysis. The airflow field was solved using the incompressible flow assumption, and aroma particles were tracked as discrete passive particles with superimposed random perturbation terms from the local flow field. Collision intensity was calculated based on the spatial density of particle‐wall/near‐wall contacts, weighted by local transport intensity, and finally normalized to a 0–1 range for visualization. Key parameters and data sources are summarized in Table S13.

### 4.8 Monitoring of Retronasal Aroma During the Oral Processing of Grilled Lamb Skewers

The aroma profile released into the nasopharynx during the oral processing of lamb skewers with different grilling times was investigated using a high‐resolution BreathSpec GC‐IMS system (GAS, Dortmund, Germany). The method was adapted from Pu et al. [17] and Wang et al. [19]. Eight panelists followed the bolus collection procedure described previously. They chewed 25 g portions of the three types of lamb skewers with their lips closed. At 10, 30, and 60 s of chewing, they were instructed to exhale steadily for 3 s through one nostril into the side port of a 500 mL Tedlar PVF sampling bag. The gas collected in the bag was then transferred to the inlet of the BreathSpec GC‐IMS.

Prior to switching a six‐port valve, a 1 mL gas sample from a built‐in inert sample loop was injected into an MXT‐WAX capillary column (15 m × 0.53 mm, 1 µm film thickness, Restek, Pennsylvania, USA). The column temperature was maintained at 70°C. High‐purity nitrogen (>99.99%) served as both the carrier and drift gas. The carrier gas flow rate was programmed as follows: initial flow of 5 mL/min held for 2 min, increased to 50 mL/min over 10 min, then to 100 mL/min over 8 min, and finally to 140 mL/min over 10 min. The IMS ionization source was a ^3^H radioactive source (300 MBq). The drift tube temperature was set at 45°C, with a field strength of 500 V/cm and a drift voltage of 6.5 kV.

### 4.9 Electroencephalography (EEG) Signals for Retronasal Olfaction

#### 4.9.1 EEG Data Acquisition

To further elucidate the role of retronasal olfaction in flavor integration and enhancement during the oral processing of lamb skewers, EEG was employed to track changes in brain activity associated with aroma perception. All panel members participated in this experiment. Participants were instructed to maintain their regular sleep pattern the day before the test and obtain sufficient sleep (≥7 h) the night prior. Compliance was verbally confirmed upon arrival. They were also required to abstain from alcohol and caffeinated beverages for at least 12 h before the session. Prior to the experiment, all participants received a detailed briefing on the procedures and provided written informed consent. Monitoring equipment was worn throughout the session.

A 32‐channel saline‐electrode EEG system integrated with physiological signal sensors for respiratory frequency (RESP), electrocardiogram (ECG), and electrodermal activity (ErgoLAB, Kingfar Technology Inc., Beijing, China) was used to record brain activity during chewing and bolus formation. As shown in Figure S10, electrodes were arranged according to the International 10–20 system, covering the frontal, left temporal, right temporal, central, and parieto‐occipital regions [14]. Electrode sites were applied with saline solutions to reduce impedance. The sampling frequency was set at 256 Hz. All equipment was calibrated and tested before the formal experiment to ensure proper operation. The experiment was conducted in a well‐ventilated laboratory. The ventilation system remained active throughout to maintain air circulation, which was crucial for preventing the accumulation of odorants and cross‐contamination between consecutive samples.

The formal test consisted of two phases. In the first phase, participants placed a 25 g sample of lamb skewer (with varying grilling times, presented in random order) into their mouth following an on‐screen prompt. They then chewed the sample at a fixed frequency of 1.4 chews per second for a 60‐s signal acquisition period, as indicated on the screen. During acquisition, participants kept their eyes closed and remained as still as possible. After collecting, a “Rest” prompt appeared. Participants then expected the sample, rinsed their mouth to clear any taste residue, and entered a 2‐min rest interval before the next “Prepare Sample” instruction. In the second phase, participants wore a nose clip. They then placed and chewed the three types of lamb skewers to acquire signals, following the same procedure as the first phase. Upon completion, participants were free to remove the sensors and leave.

#### 4.9.2 EEG Data Processing for Retronasal Olfactory Perception

The raw 32‐channel EEG signals were processed using the MNE toolkit in Python. To investigate the olfactory perception patterns during the mastication of lamb skewers with different grilling times, the continuous 60‐s EEG signals were segmented into three non‐overlapping time windows. This segmentation ensured temporal independence for subsequent time‐series modeling. During the preprocessing stage, a band‐pass filter was applied. A high‐pass filter with a cut‐off frequency of 0.5 Hz was used to eliminate low‐frequency drifts, while a low‐pass filter with a cut‐off frequency of 45 Hz was applied to suppress high‐frequency noise. Independent component analysis was subsequently employed to remove ocular artifacts while preserving the primary information of the original EEG signals. Finally, the time‐domain signals were transformed into the frequency domain using the fast Fourier transform, as expressed in the following formula:

(8) $$X\left(f\right)=\int_{-\infty }^{\infty }x\left(t\right)\;e^{-j2\pi ft}dt$$

here, *x*(*t*) is the time‐domain signal, *X*(*f*) is the frequency‐domain signal, where *f* represents frequency, and *j* denotes the imaginary unit.

Artifacts from cardiac, muscular, and respiratory activity were corrected in steps using synchronously recorded ECG, EMG, and respiratory signals. EEG artifacts induced by cardiac pulses were removed by constructing an average template aligned with R‐peaks and applying weighted subtraction, with the correction formula as follows:

(9) $$EEG^{\left(1\right)}\left(t\right)=EEG_{\mathrm{raw}}\left(t\right)\;-\;\sum_{i}\alpha_{i}\;\times \;\mathrm{Temp}l_{\mathrm{ECG}}\left(t\;-\;\tau_{i}\right)$$

where Templ_ECG_ represents the ECG‐triggered EEG average template, α_*i*_ is the adaptive weight, and τ_*i*_ is the time delay.

This step primarily suppressed low‐frequency ECG interference, making the EEG signal more stable on the respiratory cycle scale. Further, it utilized the masticatory muscle EMG signals to clean up EMG artifacts in the EEG, with the correction formula as follows:

(10) $$EEG^{\left(2\right)}\left(t\right)=EEG^{\left(1\right)}\left(t\right)\;-\;\mathrm{CC}A_{\mathrm{proj}}\left(\mathrm{EM}G_{\mathrm{jaw}/\mathrm{bite}}\left(t\right)\right)$$

where CCA_proj_ represents the estimation of electromyographic artifacts projected into EEG space through canonical correlation analysis.

This step targeted high‐frequency myogenic disturbances (>20 Hz). Respiratory signals were then used to correct for low‐frequency baseline drift and motion artifacts:

(11) $$EEG_{\mathrm{final}}\left(t\right)=EEG^{\left(2\right)}\left(t\right)\;-\;\gamma \;\mu \;\left(\mathrm{Resp}\left(t\right)\;\times \;h_{\mathrm{filter}}\left(t\right)\right)$$

where Resp(*t*) is the standardized respiratory waveform, *h*_filter_(*t*) is the filter response, and γ is the dynamic gain.

This step ensured the purity of EEG signals on a slow‐varying scale while compensating for the cumulative effects of chemical background.

To address the potential local artifacts caused by wearing a nasal clip, this study constructed an artifact template using the EEG difference between conditions with and without the nasal clip. The nasal clip artifact template was obtained by calculating the time‐averaged difference between the EEG signals under the nose‐clip condition, *EEG*_clip_(*t*), and those under the no‐nose‐clip condition, *EEG*_no‐clip_(*t*), across multiple experimental trials, as shown in the following formula:

(12) $$\mathrm{Temp}l_{\mathrm{NoseClip}}\left(t\right)=\left\langle EEG_{\mathrm{clip}}\left(t\right)-EEG_{\mathrm{no}-\mathrm{clip}}\left(t\right)\right\rangle$$

where ⟨·⟩ denotes the average over multiple repeated experiments.

Subsequently, the template was subtracted from the EEG signal *EEG*_final_(*t*) after the elimination of ECG, EMG, and respiratory artifacts using an adaptive gain coefficient *β*, to obtain the EEG signal after nasal‐clip artifact correction:

(13) $$EEG_{\mathrm{corrected}}\left(t\right)=EEG_{\mathrm{final}}\left(t\right)\;-\;\beta \;\times \;Templ\;\left(t\right)$$

The gain *β* is optimized within baseline and low‐artifact windows by minimizing residual variance after correction, rather than by treating the no‐nose‐clip signal as an absolute physiological ground truth.

This method utilized the subject's own control data to effectively extract and eliminate specific artifacts caused by the nasal clip while preserving neural signals.

After the sequential removal of electrocardiographic, electromyographic, respiratory, and nasal‐clip artifacts, the resulting EEG signals may still contain residual systematic noise unrelated to the retronasal olfactory stimulus itself. To further refine the neural responses specifically associated with retronasal olfaction, this study introduced an artifact removal adaptive subtraction template. This method used EEG from a pre‐stimulus baseline period without aroma as a control to adaptively estimate and subtract non‐neurogenic background components. Specifically, a segment of resting baseline EEG was recorded immediately before each retronasal olfactory stimulation trial, denoted as *EEG*_baseline_(*t*). The adaptive subtraction template for retronasal olfactory artifacts was then constructed by averaging these baseline segments across multiple experimental trials:

(14) $$\mathrm{Temp}l_{\mathrm{AEAST}}\left(t\right)=\left\langle EEG_{\mathrm{baseline}}\left(t\right)\right\rangle$$

where ⟨·⟩ denotes the average over multiple repeated experiments.

Subsequently, the template was subtracted from the EEG signal *EEG*_corrected_(*t*) that had undergone all preceding artifact removal steps using the adaptive gain coefficient *λ* to obtain the final purified nasal olfactory EEG signal:

(15) $$EEG_{\mathrm{retro}}\left(t\right)=EEG_{\mathrm{corrected}}\left(t\right)\;-\;\lambda \;\times \;\mathrm{Temp}l_{\mathrm{AEAST}}\left(t\right)$$

The gain coefficient λ is adaptively estimated by minimizing the power of *EEG*_retro_(*t*) within the baseline window, that is, $\lambda \;=\frac{\mathrm{Cov}(EEG_{\mathrm{corrected}},\mathrm{Temp}l_{\mathrm{AEAST}})}{\mathrm{Var}(\mathrm{Temp}l_{\mathrm{AEAST}})}$. This ensures that template subtraction only eliminates background components that were consistent with the baseline, without damaging stimulus ‐ induced neural responses. To avoid removing slow stimulus‐related neural activity, lambda was estimated only from the pre‐stimulus baseline window and applied as a conservative correction for components that were temporally consistent with baseline activity.

This method used the subject's own odorless stimulation baseline as an internal control, eliminating the need for additional reference electrodes or external devices. While preserving the time‐domain and frequency‐domain characteristics of retronasal olfactory evoked potentials, it effectively removed residual non‐neurogenic interferences such as respiratory airflow and mucosal impedance fluctuations, ultimately outputting pure retronasal olfaction‐related EEG signals (operationally defined as the CH‐NC minus CH‐C difference after artifact correction).

To evaluate the energy distribution of EEG signals across different frequency bands (*δ*, *θ*, *α*, *β* waves), power spectral density analysis was employed, with the calculation formula as follows:

(16) $$P\left(f\right)=|X\left(f\right){|}^{2}$$

where *P*(*f*) represents the power spectral density at frequency *f*, and ∣*X*(*f*)∣ is the amplitude of the frequency domain signal.

By extracting and quantifying conventional EEG frequency bands—delta (0.5–4 Hz), theta (4–8 Hz), alpha (8–14 Hz), beta (14–30 Hz), and gamma (30–45 Hz)—from the power spectral density, spectral features were generated. The extraction of key features such as EEG band energy and peak frequency provides rich input features for subsequent deep learning modeling.

Additionally, to isolate the specific contribution of retronasal olfaction, we acquired two sets of control data based on previously established EEG paradigms. Following the protocol validated in our prior study on orthonasal aroma perception [14], an orthonasal control (sniffing without chewing) experiment was conducted. Simultaneously, a tasteless texture‐matched chewing control experiment was implemented based on our earlier research on oral processing [48]. All samples were maintained at 50°C to eliminate temperature effects.

#### 4.9.3 EEG Source Localization Analysis

The distribution of electrical sources within the brain during food perception was estimated using standardized low‐resolution brain electromagnetic tomography (sLORETA). The source localization of EEG signals was performed using the MNE‐Python software, an open‐source Python package that integrates dipole fitting and distributed source localization techniques. The acquired EEG signals were averaged. Within 0–60 s EEG data, signals representing the most significant brain responses were selected as key indicators of activation and used for source localization. The forward problem was solved using a realistic head model based on the Montreal Neurological Institute template, while the inverse problem was addressed using sLORETA.

#### 4.9.4 Interpretable Analysis of EEG Using SHAP

Shapley additive explanation (SHAP) is a key method for interpreting machine learning and deep learning models, particularly the predictions of complex or black‐box models [23]. The SHAP framework was grounded in cooperative game theory and utilizes Shapley values. A Shapley value quantifies the contribution of a feature by considering all possible combinations (or “coalitions”) of features. It represents the average marginal contribution of that feature across all potential coalitions. Consequently, the overall contribution of each feature to a model's output can be quantified by aggregating these individual values. In this study, SHAP was applied to interpret the key EEG‐based features underlying the perception of retronasal aroma in grilled lamb skewers.

### 4.10 Construction of the Physics‐Guided Dual‐Timescale Cross‐Field Network (PG‐DTCFN)

To address the significant disparity in sampling frequencies among multimodal data (ranging from sub‐second to minute scales) and to explicitly model the complex coupling between aerodynamics and the nasopharyngeal mucus‐chemical field during respiration, a novel deep learning framework named the physics‐guided dual‐timescale cross‐field network (PG‐DTCFN) was proposed. The core innovation of this architecture is a dual‐path heterogeneous feature alignment mechanism. It integrated sparse and dense temporal signals of different physical meanings through physics‐constrained encoding, temporal‐scale alignment modules, and cross‐modal residual coupling. The framework ultimately achieved accurate prediction of retronasal sensory intensity under the guidance of a unified aroma stimulation model. The overall architecture of PG‐DTCFN is illustrated in Figure 6.

To make the mathematical basis of PG‐DTCFN explicit, the model was formulated as a physics‐guided state‐space residual learning framework rather than an empirical stacking of modules. The fast stream was defined on a high‐resolution model axis  *t_i_*  =   *i*/20 s as ***x**_f_* (*t_i_*)  =  [***v*** (*t_i_*), ∇ ***v*** (*t_i_*), ***ω*** (*t_i_*), EEG(*t_i_*), *F*_bite_(*t_i_*)], where ***v*** is airflow velocity,  ∇ ***v*** is the local velocity gradient, ***ω *** =  ∥∇  ×  ***v*** ∥ is vorticity, EEG(*t_i_*) denotes band‐power features, and *F*_bite_(*t_i_*) is bite force. The slow stream was defined at sparse observation times τ_*k*_ as **x**_*s*_(τ_*k*_)  =  [**C**(τ_*k*_), MUC5B(τ_*k*_), MUC7(τ_*k*_), pH(τ_*k*_), **B**(τ_*k*_)], where **C** is the GC‐IMS volatile profile, and **B** denotes bolus physicochemical descriptors.

The physics‐constrained encoding layer followed the transport, adhesion, and effective‐exposure concepts used in nasal CFD and exposure‐dose modeling. The transport representation was written as $h_{\mathrm{trans}}\left(t_{i}\right)=F_{\mathrm{conv}}\;\left(v\left(t_{i}\right),\nabla v\left(t_{i}\right)\right)\;\times \;\omega \left(t_{i}\right)$, describing convective transport and rotational mixing. The adhesion representation was written as h_adh_(τ_*k*_)  =  σ(*W*_adh_ *M*(τ_*k*_)  +  *b*_adh_), where *M*(τ_*k*_)  =  [MUC5B(τ_*k*_), MUC7(τ_*k*_)] and σ is a nonlinear activation function describing mucin‐mediated retention. The volatile accessibility correction is expressed as *h*_acc_(*t_i_*)  =  Softmax(*Q*(**C**)*K*(**v**)^*T*^)*V*(**C**), which maps VOCs abundance to an airflow‐modulated effective exposure spectrum. These terms were grounded in concentration‐velocity flux integration, wall interaction/shear stimulation in nasal CFD, and mucin‐mediated aroma adsorption/desorption theory.

The slow‐path memory was updated only when a sparse observation arrives: $h_{k}=\mathrm{LSTM}\left(\phi_{s}\left(x_{s}\left(\tau_{k}\right)\right),h_{k-1}\right)$, and was propagated to the fast time axis by a neural ODE: $\frac{dh(t)}{dt}=f_{\theta }\;\left(h(t),\Delta t,u(t)\right)$, yielding *z_s_* (*t_i_*)  =  *W_s_* h(*t_i_*)  +  b_*s*_. The fast feature $z_{f}\left(t_{i}\right)=\phi_{f}\;\left(x_{f}\left(t_{i}\right)\right)$ was then fused with the slow feature by gated residual coupling: *z*(*t_i_*)  =   (*t_i_*)  +  *g*_θ_(z_*f*_(*t_i_*), z_*s*_(*t_i_*))⊙z_*s*_(*t_i_*). The final intensity was modeled as *I_R_* (*t_i_*)  =  *I*_phys_ (*t_i_*)  +  Δ*I*_NN_(*t_i_*)  +  λ*I*_adh_(*t_i_*), where *I*_phys_ is the physical exposure baseline, Δ*I*_NN_ is the residual learned from multimodal observations, and λ*I*_adh_ represents the mucin‐retention contribution. Thus, PG‐DTCFN combines physically interpretable priors with data‐driven residual correction.

#### 4.10.1 Heterogeneous Granularity Multimodal Input Layer

The input layer of the model was divided into two parallel data streams based on the physical meaning and sampling frequency of the signals. This design preserved the native granularity and informational integrity of raw data. The high‐resolution dynamic stream *x_f_* (*t_i_*) received variables synchronized on a 20 Hz model time axis, *t_i_*  =  *i*/20*s*, capturing physical and physiological fluctuations at second or sub‐second scales. Here, 20 Hz denotes the temporal resolution used for input synchronization and multimodal fusion, not the EEG spectral high‐frequency band. EEG was first decomposed into conventional spectral features and then aligned with bite force and aerodynamic variables on the same model axis. This stream includes aerodynamic features (velocity field, shear gradient, vorticity), EEG spectral‐response features, and bite force. The low‐frequency sparse stream *x_s_* (*tau_k_*) received chemical and biological background information at their actual observation times *tau_k_* (<0.05 Hz), including GC‐IMS volatile compound profiles, MUC5B/MUC7 mucin concentration, and physicochemical properties of the lamb skewer bolus.

#### 4.10.2 Physics‐Constrained Feature Encoding Layer

To eliminate unit discrepancies among raw signals and convert raw physical quantities into mechanistic expressions with clear physical meaning, the model first processed the two data streams through a physics‐constrained encoding module. This module, inspired by governing equations from fluid mechanics and interface science, comprised three core sub‐modules.

The transport code (*E_trans_*) was designed for the airflow characteristics in high‐frequency dynamic flows, utilizing partial differential operators to simulate its convective‐diffusion process. It encoded this into a latent vector representing material transport capability, as shown in Equation (17):

(17) $$h_{trans}\left(t\right)=F_{conv}\;\left(v,\nabla v\right)\;\times \;\omega$$

where ω represents the magnitude of the vorticity field, defined as ω = ||∇ × *v*||, reflecting the modulation effect of airflow rotation intensity on the turbulent diffusion of aroma molecules. High vorticity regions correspond to enhanced airflow turbulence, facilitating the lateral transport of aroma molecules to the nasal mucosa surface.

The adhesion modulation coding (*E_adh_*) targeted the mucin concentration in the low‐frequency flow, denoted as *M*(*t_k_*), and simulates the dynamic adsorption and desorption of gaseous molecules by the mucus layer through a learnable adhesion function. It output the mucosal interface activity coefficient, as shown in Equation (18):

(18) $$h_{adh}\left(t_{k}\right)=\sigma \left(W_{adh}\;\times \;M\left(t_{k}\right)+b_{adh}\right)$$

where *σ* is the nonlinear activation function.

The volatile accessibility correction (*E_acc_*) correlated the VOCs spectrum *C*(*t_k_*) with airflow characteristics to calculate the nasopharyngeal deposition probability of different compounds under specific airflow conditions, generating a corrected effective exposure spectrum for volatile compounds, as shown in Equation (19):

(19) $$h_{acc}\left(t_{k}\right)=\text{Softmax}\left(Q\left(C\right)\times K{\left(v\right)}^{T}\right)\times V\left(C\right)$$

Through the above encoding, the original multimodal signals were mapped into a dimensionally unified latent space, forming physically enhanced feature representations *H_fast_* (*t*) *and H_slow_* (*t_k_*), laying the foundation for subsequent temporal scale alignment.

#### 4.10.3 Dual‐Timescale Modeling Based on Data Granularity

To address the timescale mismatch between fast and slow data streams, the network employed dual‐path architecture that processed them separately before unifying them onto a high‐resolution temporal axis. The fast dynamic path utilized stacked multi‐head self‐attention mechanisms to directly handle high‐frequency physically enhanced features *H_fast_* (*t*). The attention mechanism captured long‐range dependencies, effectively modeling the sub‐second dynamic coupling between airflow fluctuations and neural electrical signals, thereby extracting dynamic features *F_fast_* (*t*)​ that characterized transient respiratory disturbances. This process is expressed by Equation (20):

(20) $$\begin{array}{ccc} F_{fast}\left(t\right) & = & \mathrm{Attention}\left(Q_{fast},K_{fast},V_{fast}\right)\\ & = & \mathrm{Softmax}\left(\frac{Q_{fast}K_{fast}^{T}}{\sqrt{d_{k}}}\right)V_{fast} \end{array}$$

The other is the slow retention path, which targets minute‐level sparse data *x_s_* (*tau_k_*). To prevent sparse chemical points from being treated as repeated high‐frequency measurements, the model performed state‐space propagation rather than direct linear interpolation. For each low‐frequency observation, the sparse input was first encoded as *e_k_*  =  *phi_s_* (*x_s_* (*tau_k_*)). When a new observation arrives at *tau_k_*, the LSTM updates the slow‐memory state according to *h_k_*  =  *LSTM*(*e_k_*, *h*_*k* − 1_). Between two consecutive sparse observations, the hidden state was propagated to each high‐resolution time point by a differentiable ODE solver: *dh*(*t*)/*dt*   =  *f_t_* *heta*(*h*(*t*), *Deltat*, *u*(*t*)), and *h*(*t_i_*)  =  *ODESolve*(*h_k_*, *tau_k_*, *t_i_*). The continuous slow feature field was then obtained as *z_s_* (*t_i_*)  =  *W_s_* *h*(*t_i_*)  +  *b_s_*. In parallel, the fast path produced *z_f_* (*t_i_*)  =  *phi_f_* (*x_f_* (*t_i_*)). The two streams were finally aligned on the same *t_i_* grid through gated residual coupling, *z*(*t_i_*)  =  *z_f_* (*t_i_*)  +  *g_t_heta*(*z_f_* (*t_i_*), *z_s_* (*t_i_*))  ×  *z_s_* (*t_i_*). This procedure placed slow chemical, mucin, and bolus‐background information onto the 20 Hz model axis while preserving adsorption/desorption, mucus‐retention, and cumulative‐exposure dynamics, rather than creating artificial high‐frequency chemical measurements.

#### 4.10.4 Cross‐Domain Residual Coupling Learning

To explicitly model the complex interactions between fast airflow fields and slow chemical fields, the model introduced a cross‐modal residual coupling structure. This structure simulated the bidirectional modulation mechanism in physical processes. Specifically, dynamic modulation allowed high‐frequency aerodynamic features *F_fast_* (*t*)to perform point‐wise modulation on the low‐frequency chemical background field *F_slow_* (*t*), simulating the enhancement effect of airflow disturbances on the mass exchange rate at the bolus surface, as shown in Equation (21):

(21) $$F_{modulated}\left(t\right)=F_{slow}\;\left(t\right)⊙\left(1+\tanh\;\left(W_{gate}\;\times \;F_{fast}\left(t\right)\right)\right)$$

Simultaneously, through residual constraints, the low‐frequency feature *F_slow_* (*t*) serves as the global background state, restricting the gain of high‐frequency features via residual connections. This ensured that fluctuations in high‐frequency details do not deviate from the fundamental chemical background of the system, as shown in Equation (22):

(22) $$F_{coupled}\left(t\right)\;=\mathrm{Concat}\left(F_{fast}\left(t\right),F_{modulated}\left(t\right)\right)+\mathrm{Proj}\left(F_{slow}\left(t\right)\right)$$

This bidirectional interaction mechanism ensured that the fused feature *F_coupled_* (*t*) retained transient high‐frequency details while maintaining global accuracy reflecting long‐term system trends, all under a unified temporal scale.

#### 4.10.5 Physical Residual Correction and Intensity Prediction

At the end of the model, we incorporated physical prior knowledge for final correction and decision fusion. Based on classical exposure science theory, the cumulative physical exposure quantity *D*(*t*) was calculated. *D*(*t*) as an independent physical prior branch provided the most directly relevant physical baseline for perceived intensity. This quantity can be obtained by integrating the compound concentration carried by the airflow and the exposure time, as shown in Equation (23):

(23) $$D\left(t\right)=\int_{0}^{t}\gamma \;\times \;C\left(\tau \right)\;\times \;|v\left(\tau \right)|d\tau \;$$

where γ is the absorption coefficient.

Subsequently, the cross‐field coupling features *F_coupled_* (*t*) learned by the neural network were concatenated with the physical prior *D*(*t*), followed by nonlinear mapping through a residual fusion module. This module consisted of a three‐layer perceptron (MLP). The core idea was to enable the neural network to predict the “residual” not captured by the physical model—namely, perceptual deviations caused by complex factors such as neurophysiological responses and turbulent fluctuations. The final retronasal sensory intensity score *S*(*t*) was derived by adding the physical baseline to the residual correction from the neural network, as shown in Equation (24):

(24) $$S\left(t\right)=\mathrm{MLP}\left(\left[F_{coupled}\left(t\right),D\left(t\right)\right]\right)+\mathrm{Linear}\left(D\left(t\right)\right)$$

This design not only improved the model's prediction accuracy but also endowed it with a degree of interpretability, clarifying the respective contributions of physical laws and complex physiological responses to the final perception.

### 4.11 Construction of the Integrated Mastication–Bolus Dynamics–Nasal Respiration Simulation Framework

The formation of retronasal olfactory perception originated from the bolus created by chewing and the subsequent transport of released volatile compounds to the olfactory epithelium via the respiratory airflow through the nasopharynx. This process involved complex coupling among masticatory mechanics, bolus rheology, and nasal aerodynamics. It was challenging to obtain synchronized, high temporal‐resolution data on dynamic bite force, internal bolus structural evolution, and transient nasal airflow fields through experimental means alone. To address this limitation, a multi‐physics integrated simulation framework was developed. This framework aims to numerically reconstruct the entire process from oral processing to nasal delivery, thereby revealing the underlying coupling mechanisms between bolus formation dynamics, nasal airflow, and the release/perception of retronasal aroma. The framework operates on a unified temporal resolution with a time step of 200 Hz, enabling the synchronous coupling of bite force, bolus dynamics, and nasal airflow. The three sub‐models share the same timestamp. Specifically, Model 1 (the masticatory force–displacement coupling model) provides the driving boundary conditions for Model 2 (the particle‐spring bolus evolution model). Model 3 (the nasal respiration particle flow field model) runs its calculations independently but updates synchronously with the others. This integrated design allows for a comprehensive reconstruction of the complete pathway from oral processing to the subsequent delivery of retronasal aroma.

#### 4.11.1 Masticatory Dynamics—Point‐Coupling Model (Model 1)

This model was based on multibody dynamics and employs the Newton–Euler equations to describe the mechanical drive of major masticatory muscles [49]. The maximum muscle contraction force *F*_max_ is determined by the physiological cross‐sectional area (PCSA) of the muscle and the specific tension σ:

(25) $$F_{\max}=\mathrm{PCSA}\;\times \;\sigma$$

Subsequently, the *a* of the force‐bearing point acceleration and the rotational acceleration α are calculated according to Newton's second law, as shown in Equation (26):

(26) ∑F=ma,∑τ=Iα

where *m* is the mass, *I* is the moment of inertia, F is the resultant force, and τ is the resultant torque. The default specific muscle tension is set to σ   =  25 N/cm^2^, and the PCSA range for major human masticatory muscles is from 5 to 10 cm^2^, corresponding to a single muscle's maximum force of approximately 125–250 N. The total peak bite force ranges from 300 to 700 N. By using the ratio of the measured peak bite force from real force sensors to the model‐predicted peak as a linear scaling factor to adjust external force inputs, simulation errors can be kept below 5%.

#### 4.11.2 Particle‐Spring Bolus Evolution Model (Model 2)

The bolus was modeled as a collection of particles using the discrete element method [50]. The particle position *x*​ was updated via explicit time integration:

(27) $$x_{new}=\;x+v\Delta t+\frac{1}{2}a{\left(\Delta t\right)}^{2}$$

Particles were connected by springs, with spring forces calculated based on stiffness *k​* and elongation. Fracture occurred when the force exceeds threshold *F*_threshold_:

(28) $$F=k\left(l-l_{0}\right)n,\left|F\right|>F_{\text{threshold}}\Rightarrow \text{“Fracture”}$$

where *l*_0_​ is the initial length, and *n*​ is the unit direction vector. Default parameters: spring stiffness *k*  =  5  ×  10^4^  N/m, fracture threshold *F*_threshold_  =  0.8 N, and particle radius 0.5–1 mm. Particle aggregation probability was set to 0.25 (if spacing <1.5× initial length), with a repulsion threshold at 0.5 times the initial length. Saliva effects activated after frame 10, tripling the damping coefficient. Random perturbation amplitude was set to 0.35, and the crushing force amplitude equals half the real‐time bite force. Texture analyzer‐measured hardness and granularity curves (per minute) dynamically calibrated *k*​ and *F*_threshold_ to align simulated particle distributions with experimental data.

#### 4.11.3 Nasal Respiratory Particle Flow Field Model (Model3)

This nasal airflow model (Model 3) was based on the same three‐dimensional nasal geometry and measured respiratory data as in Section 4.7. However, it employed the Navier–Stokes equations for time‐domain solution of the flow field and reproduced the dynamic transport trajectories of aroma particles through discrete particle tracking, achieving temporal dynamic characterization within a multiphysics integrated simulation framework. With reference to the study by Li et al. [51], nasal airflow was described by the incompressible Navier–Stokes equations:

(29) $$\nabla \;\times \;u=0,\rho \;\left(\frac{\partial u}{\partial t}+u\;\times \;\nabla u\right)=-\nabla p+\mu \nabla^{2}u$$

where *u​* is the flow velocity, *ρ* is the air density (1.225 kg/m^3^), and *μ* is the dynamic viscosity (1.8 × 10^−^^5^ Pa s). Particles moved with the fluid and were superimposed with random disturbances, as shown in Equation (30):

(30) $$v_{\mathrm{particle}}=u_{\mathrm{flow}}+\mathrm{jitter}\;\times \;\mathrm{envelope}$$

The default resting flow rate was 180 mL/s, with peak respiratory flow ranging from 560 to 1100 mL/s and a respiratory frequency of 14 bpm. The velocity envelope varied with inhalation, exhalation, and pause phases, incorporating lateral disturbance components: *v_x_*  =  0.45  ×  jitter, *v_z_*  =  0.35  ×  jitter, where the jitter scale ranges from 0.9 to 1.2. The simulation set the number of particles to 1200, the trailing steps to 5, and the time step factor to 0.95. By linearly scaling the envelope amplitude and waveform based on the peak flow rate measured by a nasal anemometer, the inlet flow velocity error could be controlled within 10%.

#### 4.11.4 Submodel Coupling Method

Model 1 provided time‐varying bite force boundary conditions for Model 2, driving the bolus fragmentation evolution through the particle position update in Equation (27), with their coupling described in Sections 4.11.1,4.11.2.

The key variable transmitted from Model 2 to Model 3 is the aroma particle release flux *Q_aroma_* (*t*), calculated based on the assumption that the gas‐phase release rate of aroma molecules is proportional to the exposed surface area of the bolus. The latter was estimated in real‐time from the particle distribution parameters (NumParticles, MeanArea) output by Model 2:

(31) $$Q_{aroma}\left(t\right)=k_{release}\;\times \;S_{bolus}\left(t\right)\;\times \;C_{VOC}$$

Among them, *S_bolus_* (*t*)  =  *NumParticles*(*t*)  ×  *MeanArea*(*t*) is the total exposed area of the bolus, *C_VOC_* is the VOCs concentration measured by GC‐IMS at the corresponding time node, and *k_release_* is the rate constant of aroma release, which was calibrated by inverting the TI peak intensity of samples at each grilling time.

*Q_aroma_* (*t*) was used as the particle source intensity boundary condition at the entrance of the nasopharynx in Model 3 and was synchronously injected into the nasal flow field at a time step of 200 Hz to achieve complete coupling from food mass fragmentation to nasal transport.

### 4.12 Statistical Analysis

All data were performed using SPSS version 26.0 software (SPSS Inc., Chicago, IL) and were expressed as mean ± standard deviation (SD). For all characteristics of lamb skewers, one‐way analysis of variance (ANOVA) and Duncan's multiple range test were used to evaluate significant differences (*p* < 0.05) between samples. For the characteristics of bolus, a linear mixed model was applied, followed by Tukey's post hoc analysis. Chewing time and grilling time were treated as fixed factors, with participants included as a random factor. The interaction between chewing time and grilling time was not examined since they were independent factors. The CNSknowall platform (https://cnsknowall.com), a comprehensive web service for data analysis and visualization, was utilized. The models were developed on an Ubuntu 22.04 system using Python 3.10.2, PyTorch 2.9.1, and the CUDA 12.6 parallel computing platform, with support from the MMCV 1.6.0 computer vision library. The experimental hardware environment consisted of an Intel i7 14700K processor, an NVIDIA RTX 4090 24GB GPU, and 48GB DDR5 6000MHz RAM. For each model, the data were partitioned by randomly sampling participants, with participants allocated at the subject level to training and validation sets in an 8:2 ratio, ensuring that the validation participants were not included in model training. This subject‐level split was used to evaluate whether parameters learned from training participants could predict responses from unseen participants; individual differences were modeled through physics‐constrained input calibration [42.1] and residual correction rather than by estimating a population‐level distribution from the 20 participants. Each distinct training dataset underwent the same processing approach.

## Author Contributions

**Che Shen**: conceptualization, investigation, writing – original draft, writing – review and editing, visualization, methodology, formal analysis, software, data curation. **Xiongfeng He**: formal analysis, data curation, investigation. **Zihao Li**: data curation, formal analysis. **Lizhang Wu**: data curation. **Zidong Chen**: formal analysis, software, visualization, methodology, validation. **Jiajin Sun**: investigation, data curation. **Yunhui Zhang**: investigation, data curation. **Bo Wang**: conceptualization, methodology, visualization, formal analysis, software, supervision, writing – review and editing. **Kezhou Cai**: funding acquisition, writing – review and editing, validation, supervision, project administration, resources. **Xinyu Jiang**: data curation, investigation. **Ran Wang**: data curation. **Jingnan Lu**: data curation. **Baocai Xu**: supervision, project administration.

## Ethics Statement

The study was approved by the Biomedical Ethics Committee of Hefei University of Technology [HFUT20260105002H].

## Conflicts of Interest

The authors declare no conflicts of interest.

## Supporting information




**Supporting File 1**: advs76523‐sup‐0001‐SuppMat.pdf.

**Supporting File 2**: advs76523‐sup‐0002‐VideoS1‐S3.zip.

## Data Availability

Data will be made available on request.
