# Supplementary material for: Multiscale Coupling From Mastication to Retronasal Aroma Perception: The PG‐DTCFN Model and Multiphysics Simulation
Source: Adv Sci (Weinh). 2026 Jul 17;13(55):e76523. doi: 10.1002/advs.76523 (PMC13379262; doi:10.1002/advs.76523)
Supplement: Supplementary file 1 [file ADVS-13-e76523-s001.pdf]

Supplementary information

Multiscale coupling from mastication to retronasal aroma perception: The PG-DTCFN model and Multiphysics simulation

Che Shen[a], Xiongfeng He[a], Zihao Li[a], Zidong Chen[c], Jiajin Sun[a], Xinyu Jiang[a], Yunhui Zhang[a], Lizhang Wu[a], Ran Wang[a], Jingnan Lu[a], Bo Wang[b], Kezhou Cai[a*], Baocai Xu[a].*

[a] Engineering Research Center of Bio-process, Ministry of Education, Hefei University of Technology, Hefei 230009, China.

[b] College of Food Science and Technology, Bohai University, Jinzhou 121013, China.

[c] School of Computer Sciences, Universiti Sains Malaysia, Gelugor, Penang 11800, Malaysia.

* Corresponding author

Kezhou Cai, Ph.D./Professor, E-mail: kzcai@hfut.edu.cn

Bo Wang, E-mail: daqingwb@163.com

Supplementary Text

1. Design and experimental verification of the core module for multi-physics field coupling simulation in retronasal aroma perception system

This chapter systematically presents the model architecture and design logic of the three core modules in the postnasal aroma perception system discussed in the main text: the algorithm module (olfactory perception prediction), the respiration module (nasal airflow transport), and the mastication module (food breakdown and bite force). These three modules are not merely independent technical components but rather form an integrated, cross-scale coupling framework centered around the complete chain from "bolus release to central perception." While ensuring the interpretability of physical mechanisms, this framework achieves real-time synchronization across multiple physics fields—from masticatory mechanics to molecular transport and perceptual prediction. The following sections provide detailed discussions on the model architecture, design principles, and methodological advantages of each module.

1.1 Algorithm module: Physically Guided Dual-Time-Scale Heterogeneous Neural Network (PG-DTCFN)

1.1.1 Module positioning and core challenges

The algorithm module holds a central position at the perception end within the overall simulation framework. Its fundamental task is to transform multimodal sensory data from the physical layer—including airflow velocity fields, volatile compound concentrations, electroencephalogram signals, bite force, and bolus physicochemical properties—into quantifiable predictions of retronasal perception intensity. This task is challenging due to three

fundamental conflicts.

First, there is a conflict between physical consistency and data-driven approaches. Purely data-driven deep learning methods can capture complex nonlinear relationships, but their predictions often lack physical constraints. This can lead to anomalous outputs that violate basic laws of fluid transport and may cause overfitting across different subjects. Conversely, purely physical models have clear physical meaning but struggle to incorporate individual differences in neural responses and complex physiological regulatory mechanisms.

Second, fusing signals across multiple time scales is difficult. Different sensors have vastly different sampling frequencies. High-frequency dynamic data, such as airflow velocity, EEG signals, and bite force, can reach 20 Hz, capturing transient fluctuations at sub-second scales. In contrast, low-frequency chemical data, like volatile compound concentration and mucin content, update at less than 0.05 Hz (only 1-2 times per minute), reflecting the slowly varying global state of the system. Effectively aligning and fusing signals with such disparate temporal scales is a core technical challenge for the algorithm module.

Third, there is a lack of modeling for physiological adaptability and individual differences. Olfactory perception is not a static process. It is dynamically regulated by various physiological factors, including olfactory adaptation (sensitivity reduction due to prolonged exposure), receptor desensitization, differences in individual chewing behavior, and variations in salivary secretion characteristics. Traditional models often simplify or completely ignore these factors as fixed parameters, limiting prediction accuracy.

1.1.2 Model architecture design

To address the challenges, this paper proposes the PG-DTCFN. The design logic of this architecture follows the mechanistic principle that "physics determines the baseline, physiology determines the fluctuations." It consists of three core components.

The most fundamental is the construction of a unified physics baseline. At the model's lowest level, a physical baseline is first built based on fluid transport theory. Specifically, the velocity field of airflow, the concentration field of volatile compounds, and nasal anatomical features are integrated over space and time within a respiratory cycle. Local airflow shear effects, wall attenuation effects, and velocity magnitude effects are considered to form a dose prediction baseline that conforms to the physical laws described by the Navier-Stokes equations. The primary role of this baseline is to provide a physically reasonable initial condition for subsequent neural network learning, preventing the model output from deviating from basic physical laws.

The alignment and fusion of dual-stream multimodal inputs are of paramount importance. As shown in Figure A1, to address the fusion challenge of multi-timescale signals, the model employs a dual-stream input structure. The high-frequency stream receives instantaneous data—such as airflow velocity, shear gradient, EEG band energy, bite force, and chewing amplitude—at a 20 Hz sampling rate. A multi-head self-attention mechanism captures transient dynamic features at the sub-second scale. The low-frequency stream receives data—including volatile compound concentration, mucin content, and bolus physicochemical properties—at a sampling rate below 0.05 Hz. A memory integration module is used to "smoothly" diffuse the sparse chemical and physiological information onto the complete high-frequency timeline. After independent encoding, the two streams are aligned and fused via a cross-field attention

mechanism, forming a unified feature representation.

The physical residual correction mechanism ensures reliable model output. Based on the physical baseline, the model uses a neural network to learn a residual correction term. The core idea of this design is to let the neural network focus on learning the "residual"—the dynamic fluctuations caused by physiological factors like chewing behavior, salivary secretion, and neural response thresholds that the physical model cannot capture. The final output is composed of the physical baseline added to the neural network residual. This approach ensures both the physical plausibility of predictions and the ability to model complex physiological processes.

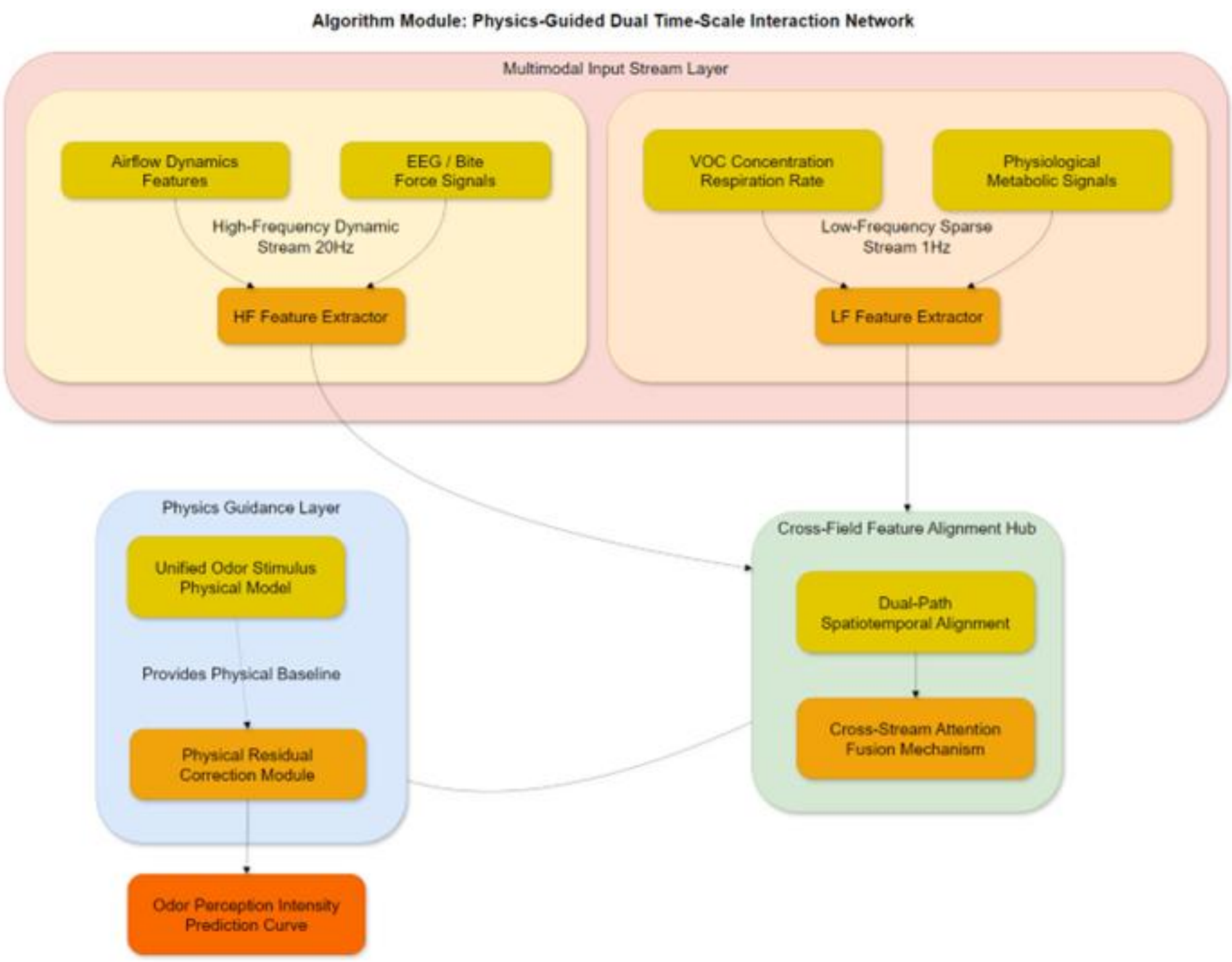

Figure A1 Schematic diagram of the PG-DTCFN dual-stream heterogeneous network architecture

1.1.3 Analysis of methodological advantages

Table A1 provides a multi-dimensional comparison between the PG-DTCFN and

traditional prediction methods. Purely physical models offer the highest interpretability, but their prediction accuracy is limited due to an inability to incorporate neural responses and individual differences. Traditional deep learning methods are flexible and can teach complex nonlinear relationships, but their lack of physical constraints results in poor interpretability and a tendency to overfit. The PG-DTCFN proposed in this paper, by deeply integrating physical priors with data-driven learning, maintains medium-to-high interpretability while significantly improving the model's generalization capability and prediction accuracy.

From a design logic perspective, the core value of the PG-DTCFN lies in its approach. It does not merely "concatenate" a physical model and a neural network. Instead, guided by mechanistic understanding, it allows the two to form an organic complement. The physical baseline provides a skeleton conforming to fundamental laws, upon which the neural network fills in details of physiological fluctuations and individual differences. This design enables the model to maintain good stability across different subjects and operational conditions, addressing the weakness of poor generalizability in traditional methods.

Table A1 Multi-dimensional comparison between PG-DTCFN and traditional prediction methods

Method	Advantages	Disadvantages	Interpretability
Pure physical model	Complies with physical laws and is highly interpretable	No neural response, limited accuracy	High
Traditional deep learning	Flexible, capable of learning nonlinear relationships	Prone to overfitting, poor interpretability	Low

PG-DTCFN in this study	Balancing physical priorities with data-driven approaches for better generalization	The network structure is relatively complex, making parameter tuning more challenging.	Moderate to high

1.2 Breathing module: The synchronously coupled high-frequency particle flow field model

1.2.1 Module positioning and core challenges

The breathing module plays a critical role as the transport endpoint within the overall simulation framework. Its task is to simulate the complete physical process of aroma molecule transport from the oral cavity, retrograde through the nasopharynx, to the olfactory epithelium. It provides dynamic airflow boundary conditions and molecular collision intensity information for the algorithm module. This module is indispensable because the physical collision of aroma molecules with the olfactory epithelium is the prerequisite for olfactory perception. Without accurate simulation of nasal transport, any perception prediction would lack a physical foundation.

The core challenge for this module lies in the fundamental trade-off between computational efficiency and physical fidelity. Traditional high-fidelity Computational Fluid Dynamics methods, which solve the full Navier-Stokes equations, can accurately capture the airflow velocity field and pressure distribution within the nasal cavity. They offer the highest physical fidelity and interpretability. However, their computational cost is extremely high. Dense mesh generation and tiny timesteps result in prohibitively long simulation times for a single run. This makes it difficult to meet the real-time requirements for synchronous coupling with the chewing and algorithm modules. More importantly, the static mesh assumption cannot adapt to the dynamic disturbances of the nasal cavity boundaries caused by maxilla

displacement during chewing. This leads to discrepancies between the simulation and the actual physiological process.

1.2.2 Model architecture design

To balance fluid dynamic fidelity with real-time coupling efficiency, this paper developed a synchronously coupled high-frequency particle flow field model. The design logic of this architecture is to avoid the reliance on fine-grained, full-mesh solutions typical of traditional CFD by employing a particle tracking strategy, thereby achieving a balance between computational efficiency and physical accuracy. Its core design is described in three aspects.

First, there is high-frequency synchronization and timeline alignment. The entire simulation framework operates on a unified timestep of 200 Hz. The breathing module shares a strictly aligned timeline with the chewing and algorithm modules. This design ensures real-time interaction of multi-physics information. Changes in bite force and bolus fragmentation events output by the chewing module can be immediately reflected in the respiratory waveform at the nasal inlet. The collision intensity information output by the breathing module can be fed in real-time to the algorithm module for perception prediction.

Second, the model uses 3D geometric reconstruction based on anatomical data. The geometric foundation for the flow field simulation is a 3D nasal cavity model representing a typical anatomical structure. This study utilized the NasalSeg dataset, a 3D CT segmentation repository of the nasal cavity and paranasal sinus regions containing 130 clinical head CT scans. The nasal cavity and related structures were manually annotated and segmented at the pixel level. Based on these annotations, the Marching Cubes algorithm was used to extract 3D surface

meshes. After spatial registration and scale normalization, a representative average morphological nasal cavity model was computed. This model completely covers the airway structure from the nasal vestibule to the posterior choanae and nasopharynx, providing the anatomical basis for subsequent gas transport simulations.

Finally, a hybrid solution and particle tracking strategy is employed. At the flow field solution level, a hybrid strategy is used. The background Navier-Stokes velocity field is first solved based on the 3D geometric model to obtain the macroscopic distribution of airflow within the nasal cavity. Subsequently, a random walk model combined with an air resistance model is introduced to track the diffusion and advective transport of odorant molecules. The core advantage of this design is that it replaces the fine-grained solution of the full spatial grid required by traditional CFD with particle tracking. This significantly reduces computational complexity while preserving the key physical characteristics of molecular transport. The inlet boundary conditions are linearly calibrated based on measured respiratory waveforms. An airflow sensor monitors breathing intensity and frequency during mastication in real-time. The measured peak flow velocity is used to scale the envelope amplitude and waveform, ensuring

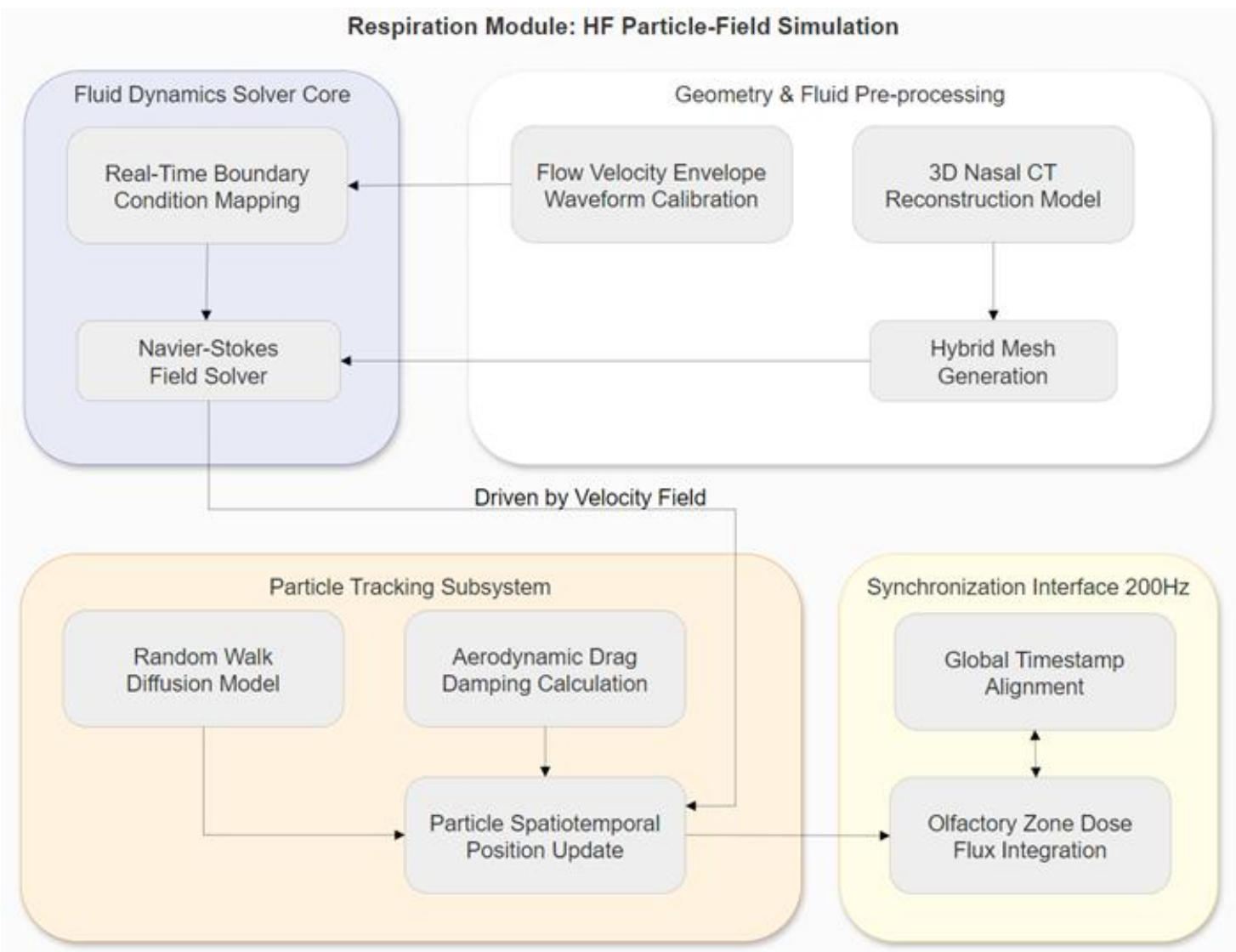

Figure A2 Particle flow field model and nasal airflow simulation schematic diagram

the simulation matches the real respiratory pattern. A schematic of this section is shown in Figure A2.

1.2.3 Analysis of methodological advantages

Table A2 compares the particle flow field model proposed in this study with traditional respiratory simulation methods. Traditional Computational Fluid Dynamics methods offer the highest physical fidelity and interpretability. However, their computational demand is immense, and they struggle to adapt to dynamic boundary changes. They are suitable for static, high-precision analysis rather than real-time coupling requirements. The Lattice-Boltzmann method operates faster but imposes high requirements on grid regularity, limiting its scalability. The particle flow field model proposed in this paper employs a hybrid solution and particle tracking strategy. It achieves real-time coupling capability with other modules while maintaining moderate accuracy, providing a feasible solution for synchronous multiphysics simulation.

Method	Advantages	Disadvantages	Interpretability	Expected performance
Traditional CFD	High precision, rich in detail	The computational load is heavy, and the grid reconstruction is complex.	High	Very high (static)
Lattice-Boltzmann	Faster computing speed	Requires a regular grid, limited scalability	Middle	Relatively high
Particle flow field model in this study	Real-time coupling with other modules is easy	Spatial resolution depends on the number of particles	Middle	Good (moderate accuracy)

Table A2 Comparative Analysis of Nasal Breathing Simulation Methods

From the perspective of methodological innovation, the key breakthrough of this module

lies in shifting the fluid dynamics solution strategy. The shift is from full-field, fine-grained calculation towards a hybrid strategy focused on capturing key features and particle tracking. This transformation enables the breathing module to interact in real-time with the chewing and algorithm modules at 200 Hz. It is no longer an isolated module that exists as an offline preprocessing step, as is typical in traditional CFD simulations.

1.3 Chewing module: Bidirectionally coupled multibody dynamics and discrete element simulation

1.3.1 Module positioning and core challenges

The chewing module occupies a central, driving position within the simulation framework. Its task is to simulate the complete process from bite force application to bolus breakdown, providing the mechanical drive for aroma release. This module is the starting point of the entire causal chain. Without accurate simulation of chewing mechanics, the physical basis for bolus evolution, aroma release, nasal transport, and ultimately perception prediction would be lost.

The core challenge faced by this module is the bidirectional coupling between mechanical driving forces and material evolution during oral processing. Real oral processing follows this physiological principle. The physical properties of the bolus, such as hardness, cohesiveness, and particle distribution, determine the chewing strategy, including bite force magnitude, chewing frequency, and mandibular movement patterns. The chewing behavior, in turn, acts upon the bolus, altering its degree of fragmentation, wetting state, and structural evolution. This closed-loop feedback, where bolus properties determine chewing strategy and chewing behavior conversely affects bolus evolution, is key to reproducing realistic oral processing.

However, existing research often treats these two aspects separately. Simplified multibody dynamics models can describe muscle-jaw mechanics well but typically assume the food is a rigid body, completely ignoring food deformation and fracture. Discrete Element Method simulations can directly obtain bolus particle distribution and structural evolution but usually lack tooth/bone structures, missing the realistic mechanical driving boundary. In vitro chewing simulators can directly test physical breakdown but are limited by device type and food category, making it difficult to achieve universal simulation modeling. A complete system that physically couples dynamic bite force with continuous phase change of the food remains a research gap in the field.

1.3.2 Model architecture design

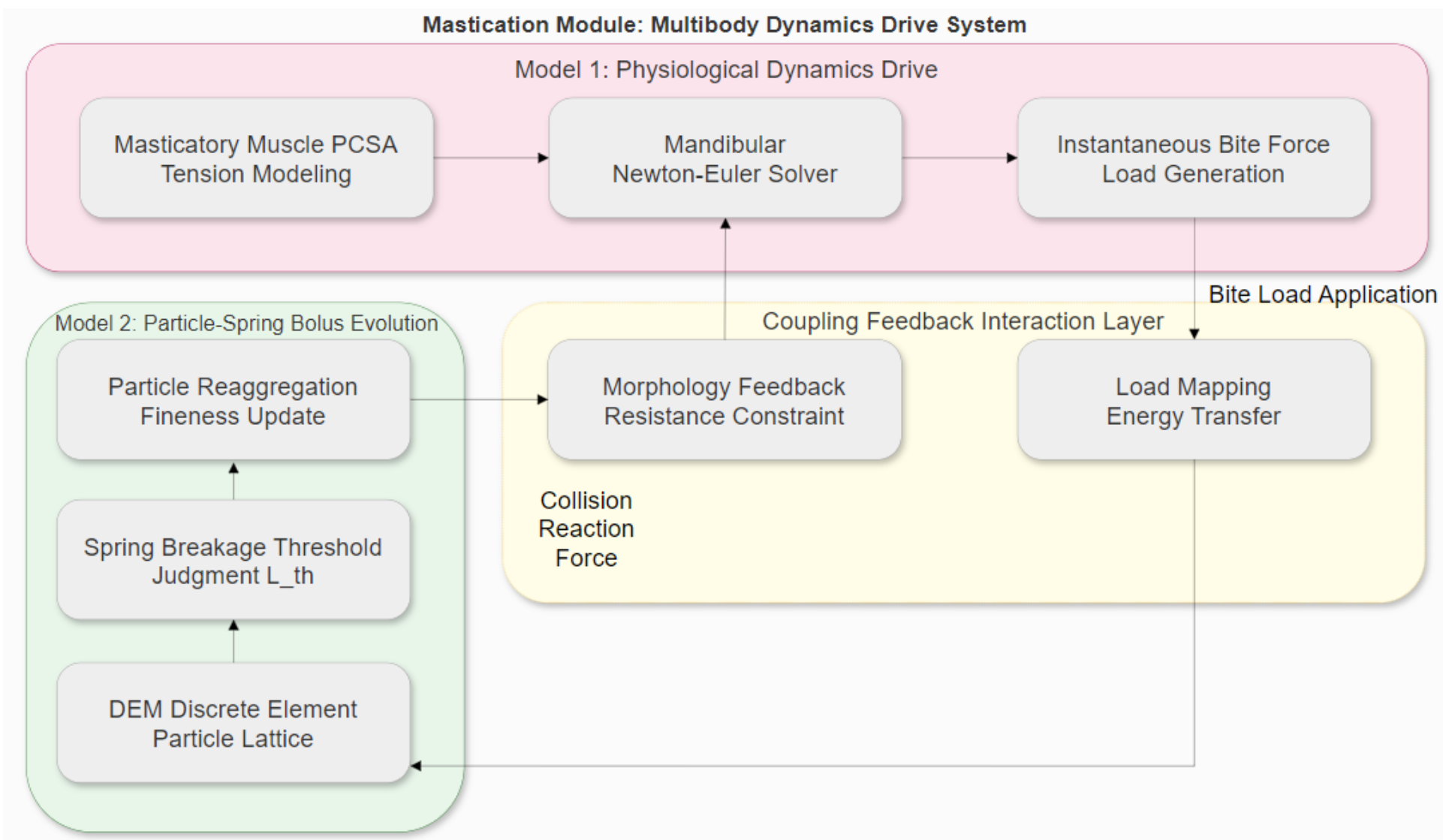

Figure A3 Schematic diagram of the bidirectional coupling simulation framework for multibody dynamics DEM

To address this gap, this paper developed a simulation architecture featuring bidirectional coupling between multibody dynamics and the Discrete Element Method. This architecture,

illustrated in Figure A3, is designed to create a closed-loop of physiological drive and physical feedback, allowing the two to interact and co-evolve in real-time during execution. Its core consists of three layers. A musculoskeletal dynamics model, a particle-spring bolus evolution model, and the bidirectional feedback loop connecting them.

The musculoskeletal dynamics model is based on multibody dynamics theory, using Newton-Euler equations to describe the mechanical drive of the major masticatory muscles. The maximum muscle contraction force is determined by the muscle's physiological cross-sectional area and specific tension. Based on human physiological data, a specific tension of 25 N/cm^2 is used. The PCSA of major human masticatory muscles ranges from 5 to 10 cm^2, corresponding to a maximum force per muscle of approximately 125 to 250 N, and a total peak bite force of 300 to 700 N. The model solves force and moment equilibrium equations in real-time to calculate the acceleration and angular acceleration of force application points, outputting dynamic bite force. The ratio between the peak force measured by a real bite force sensor and the model-predicted peak force is used as a linear scaling factor to adjust the external force input, keeping the mechanical error within 5%.

The particle-spring bolus evolution model represents the bolus as a collection of particles connected by breakable springs, modeled using the Discrete Element Method. Particle positions are updated via explicit time integration. Spring force is calculated from stiffness and elongation. Fracture is triggered when the acting force exceeds a breakage threshold, simulating the mechanical breakdown of food tissue. Default parameters are a spring stiffness of 5×10^4 N/m, a breakage threshold of 0.8 N, and a particle radius of 0.5 to 1 mm. Aggregation and repulsion rules are introduced between particles. Particles aggregate with a 0.25 probability

when their distance is less than 1.5 times the initial length, and repulsion is triggered when the distance is less than 0.5 times the initial length, simulating bonding and reshaping under saliva. The saliva effect activates after the 10th frame, increasing the damping coefficient by a factor of 3 to simulate the enhancement of bolus viscoelasticity and cohesiveness by saliva. A random disturbance amplitude of 0.35 is set, and the crushing force amplitude is set to half the real-time bite force to simulate the randomness of chewing. Hardness and particle size curves measured by a texture analyzer are used to dynamically calibrate the spring stiffness and breakage threshold, ensuring the simulated particle distribution matches real measurements.

Finally, the two models are connected to form a bidirectional feedback loop. A complete feedback circuit is established between them. The bite load output from the musculoskeletal model acts on the bolus model in real-time, driving the stretching and breaking of springs between particles. The reaction forces generated by changes in bolus topology, such as particle fracture, aggregation, and rearrangement, simultaneously update the jawbone nodes. This allows the chewing behavior to dynamically adjust based on the evolving state of the bolus. This bidirectional coupling mechanism is key to reproducing the physiological principle that bolus properties determine chewing strategy, and chewing behavior in turn acts upon bolus evolution.

1.3.3 Analysis of methodological advantages

Table A3 compares the bidirectionally coupled model proposed here with traditional chewing simulation methods. Simplified multibody dynamics models can stably describe muscle-jaw mechanics but completely ignore food deformation and fracture. While highly

interpretable, they cannot simulate realistic bolus evolution. In vitro chewing simulators can directly test physical breakdown but are limited in application scope by device and food type. DEM simulations can directly obtain particle distribution and structural evolution but typically lack tooth/bone structures, missing the realistic mechanical driving boundary. The bidirectionally coupled model proposed in this paper considers both muscle dynamics and bolus evolution. By establishing closed-loop feedback between them, it achieves, for the first time, the simulation of the complete physical process from bite force application to bolus breakdown and then to reaction force updates.

From a methodological innovation perspective, the core breakthrough of this module lies in breaking down the barrier between mandibular dynamics and food breakdown. It integrates two long-separated research areas into a unified simulation framework. This integration allows the simulation to simultaneously capture the macroscopic mechanical features driven by muscles and the mesoscopic structural changes of bolus evolution, providing accurate boundary conditions for the mechanical driving of aroma release.

Table A3 Comparison of chewing evolution simulation methods

Method	Advantages	Disadvantages	Interpretability
Simplify Multibody Dynamics	Describe the dynamics of muscle-jaw mechanics and stability	Ignore food deformation and breakage	High
In vitro chewing simulator	Direct testing of physical fragmentation is possible	Limited by equipment and food types	Limited
Discrete Element Method (DEM) Simulation	Directly obtain particle distribution	Usually does not contain dental bone structure	Middle

Coupled model (M1+M2) in this study	Considering both muscle dynamics and bolus evolution simultaneously	The computational load is heavy, with numerous parameters.	High

1.4 Module coupling and overall framework

The collaborative operation of the three modules forms a comprehensive cross-scale simulation framework, with coupling logic following a chain from mechanical driving, material evolution, to fluid transport, and finally perceptual prediction.

The chewing module sits at the forefront of the chain, using a musculoskeletal dynamics model to output dynamic bite forces, driving particle fragmentation and aggregation in the food bolus model to simulate the complete oral processing from mechanical breakdown to saliva wetting. Outputs from this stage include bite force time-series curves, food bolus particle distribution, moisture and saliva content, mucin concentration, etc. These parameters directly determine the initial release intensity and release kinetics of aroma molecules.

The respiration module receives outputs from the chewing module, particularly information on food bolus fragmentation and aroma release potential, converting them into boundary conditions for nasal airflow simulation. Based on a 3D anatomical model and measured respiratory waveforms, this module simulates the transport of aroma molecules from the oral cavity through the nasopharynx to the olfactory epithelium, outputting airflow velocity field distribution, aroma molecule collision intensity, spatial deposition patterns, and other physical quantities. This stage bridges the gap in physical mechanisms between aroma release and central perception.

The algorithm module, as the final link in the chain, integrates airflow dynamics data from

the respiration module, experimental volatile compound concentrations, and EEG signals. It achieves high-precision prediction of retronasal perception intensity through a physics-guided dual-time-scale network. The module's dual-stream structure and physical residual correction mechanism ensure that the mapping from physical-layer inputs to perceptual-layer outputs adheres to fluid transport laws while capturing dynamic fluctuations caused by individual physiological differences.

The three modules operate synchronously at a unified time step of 200 Hz, establishing real-time physical correlations between chewing mechanics, fluid transport, and neural encoding. The core contribution of this framework lies in breaking the limitations of traditional single-aspect studies, for the first time integrating macro-processing parameters, mesoscale food bolus evolution, microscopic molecular transport, and central neural responses into a unified simulation system. By injecting physical priors at the algorithmic level, adopting particle coupling strategies at the physical level, and enabling bidirectional feedback at the mechanical level, the framework maintains physical consistency while addressing the dual challenges of computational efficiency and interpretability in multi-physics coupling.

The collaborative operation of the three modules forms a complete cross-scale simulation framework, whose coupling logic follows the chain from mechanical driving, material evolution to fluid transport and finally to perception prediction.

1.5 Future prospects

Future research can deepen the framework's physiological realism and predictive capability along three main directions. At the level of physiological mechanisms, higher-order

temporal attention mechanisms could be integrated into the algorithm module. This would model effects like olfactory adaptation, the sensitivity decay from prolonged exposure, and receptor desensitization. Such an enhancement would align the perception predictions more closely with real physiological processes. At the anatomical structure level, the flow field simulation could be advanced by incorporating the periodic contraction of nasal turbinates. It could also include the dynamic perturbation of nasal cavity effective volume caused by maxillary displacement during chewing. This evolution would transition the breathing module from a static geometric model to a dynamic anatomical model. Regarding multiphase flow, the chewing module could be extended by introducing saliva rheology, to model liquid-phase lubrication, and phase-change thermodynamics, such as the endothermic melting of lipids at oral temperatures. This would refine the simulation to cover the full digestive tract pathway from the mouth to swallowing. Consequently, the bolus evolution model would expand from a dry particulate system to a wet particulate system. Progress in these directions will further enhance the application value and scientific impact of this framework within the field of food sensory science and related disciplines.

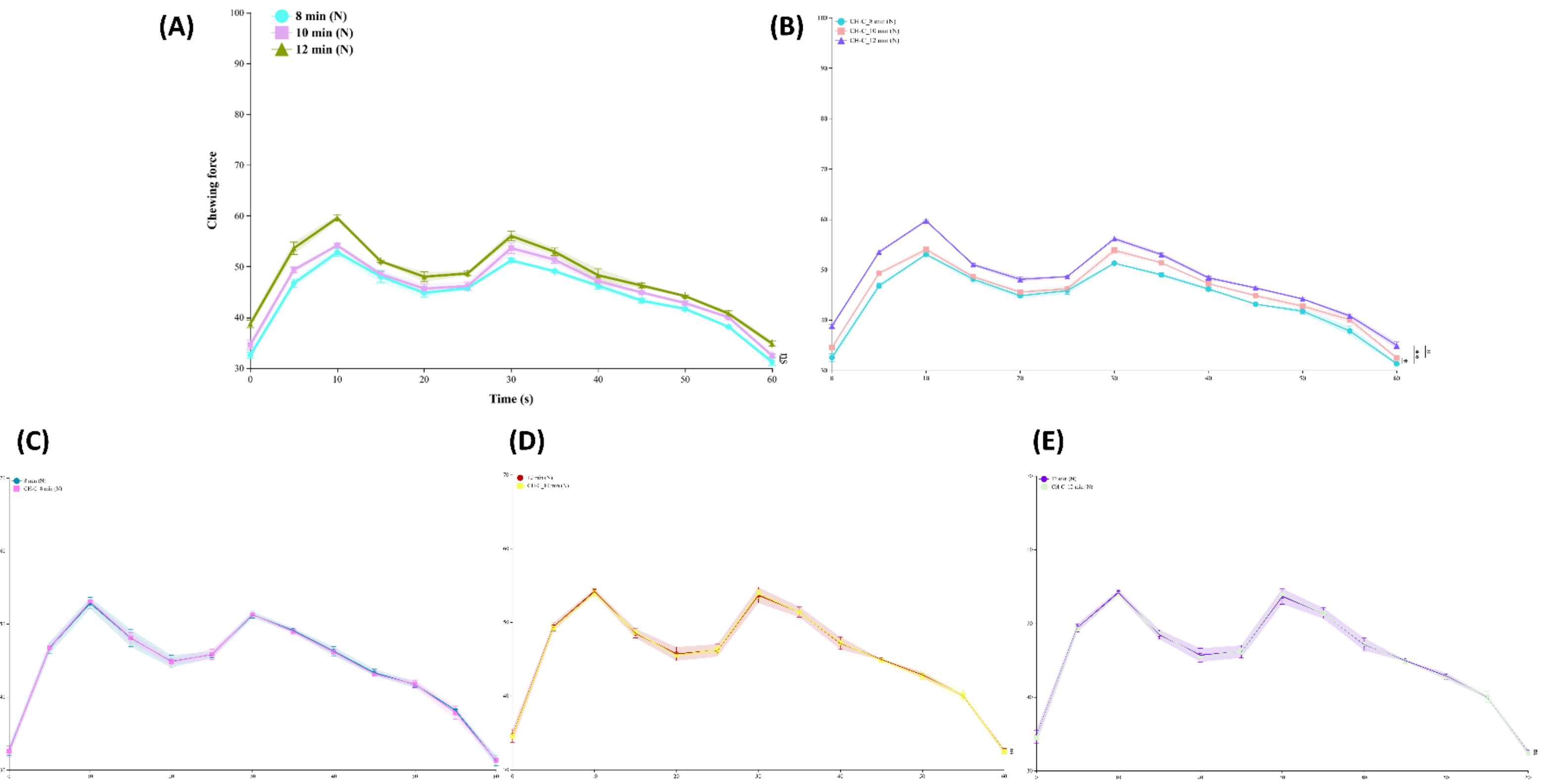

Figure S1. Temporal dynamics of chewing force during oral processing of grilled lamb skewers. (A) Chewing force profiles for samples grilled for 8, 10, and 12 min under normal chewing condition (CH-NC, without nose clip), recorded at 5-s intervals over 60 s using OpenCV-based video tracking. Data are mean ± SD (N = 20). *P < 0.05, ns: not significant (t-test). (B) Chewing force profiles for the same samples under nose-clip condition (CH-C). (C–E) Direct comparison between CH-NC and CH-C for the 8-min (C), 10-min (D), and 12-min (E) samples, respectively. No significant differences were observed between CH-NC and CH-C at any time point for any grilling time (all ns), confirming that the nose clip did not alter chewing kinematics.

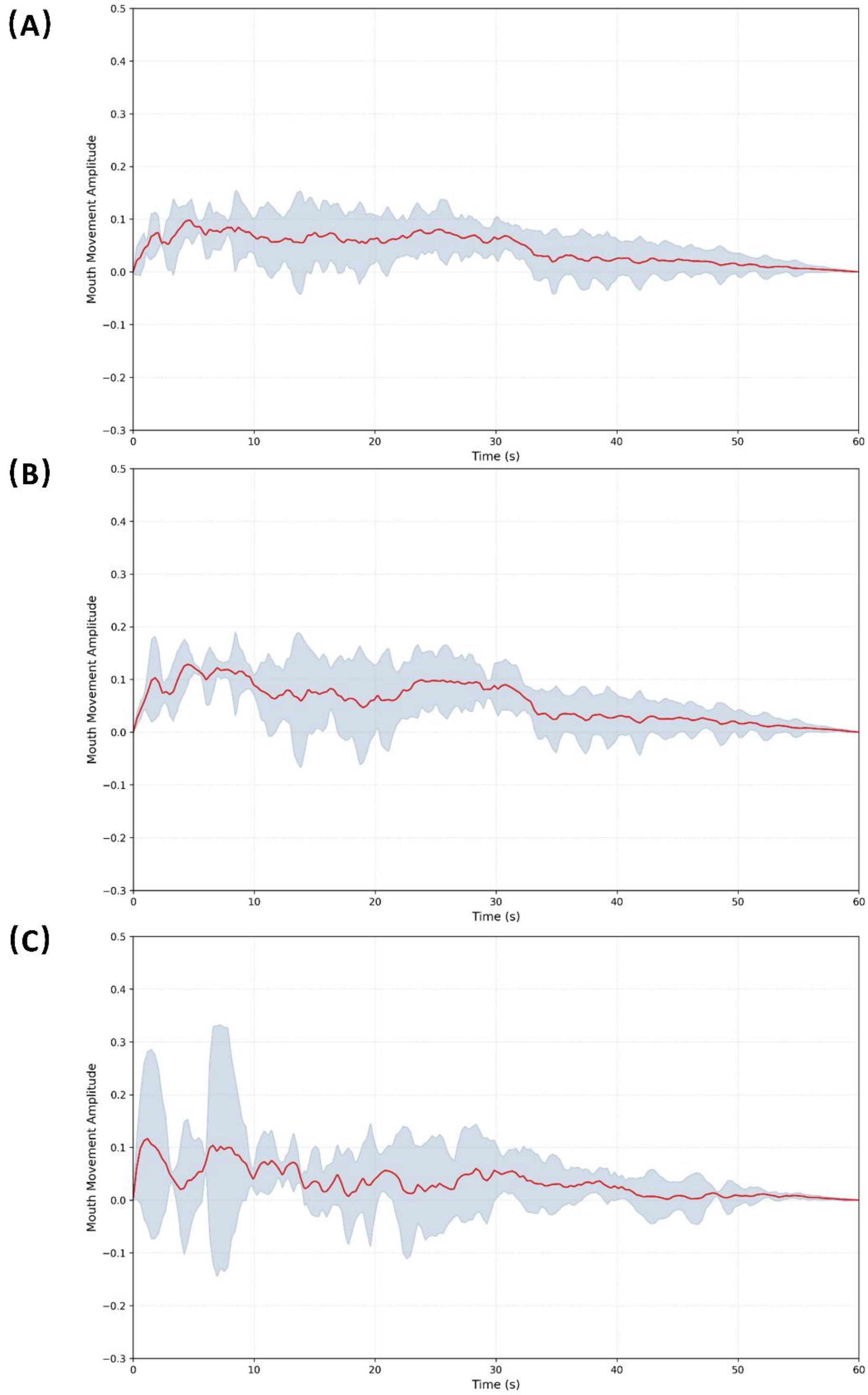

FIGURE S2. The chewing amplitude of lamb skewers with different grilling times (8, 10, and 12 minutes) during oral processing was analyzed using OpenCV-based video tracking.

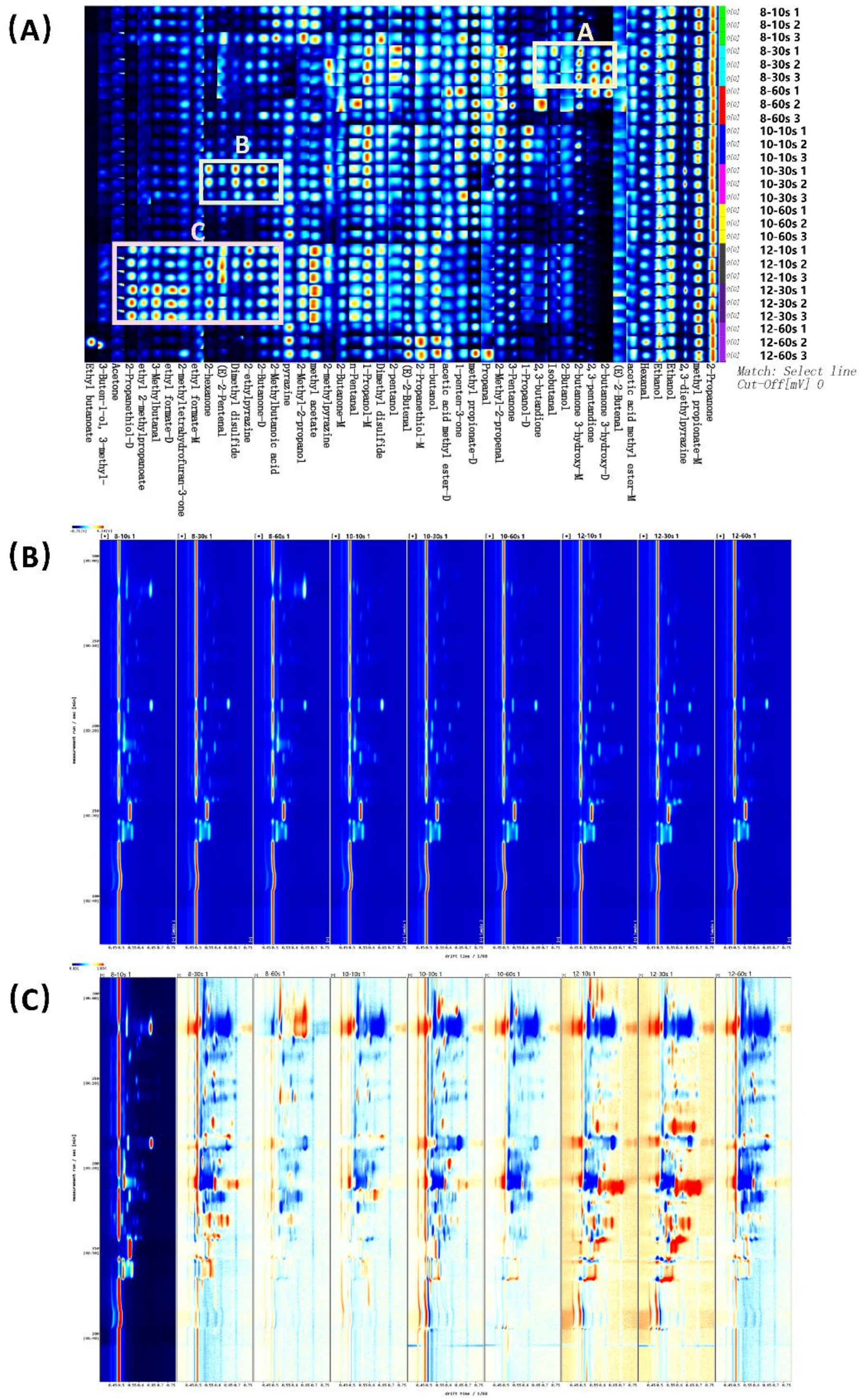

FIGURE S3. The aroma released in the retronasal cavity during oral processing of grilled lamb skewers was identified using BreathSpec GC-IMS. (A) Fingerprint of retronasal volatile compounds at different chewing times (10 s, 30 s, 60 s) for grilled lamb skewers with varying grilling times (8, 10, 12 min), and demonstrated the typical pattern of nasal volatile compound

release exhibited by three subjects (n=3) (each row represents all selected signal peaks from the grilled lamb skewer samples, and each column represents the signal peaks of the same compound at different chewing times); (B) Topographic map of grilled lamb skewers roasted for 8 to 12 minutes at different chewing times, with the vertical axis representing gas chromatography retention time and the horizontal axis representing ion mobility time; (C) Comparative difference map of grilled lamb skewers at different chewing times.

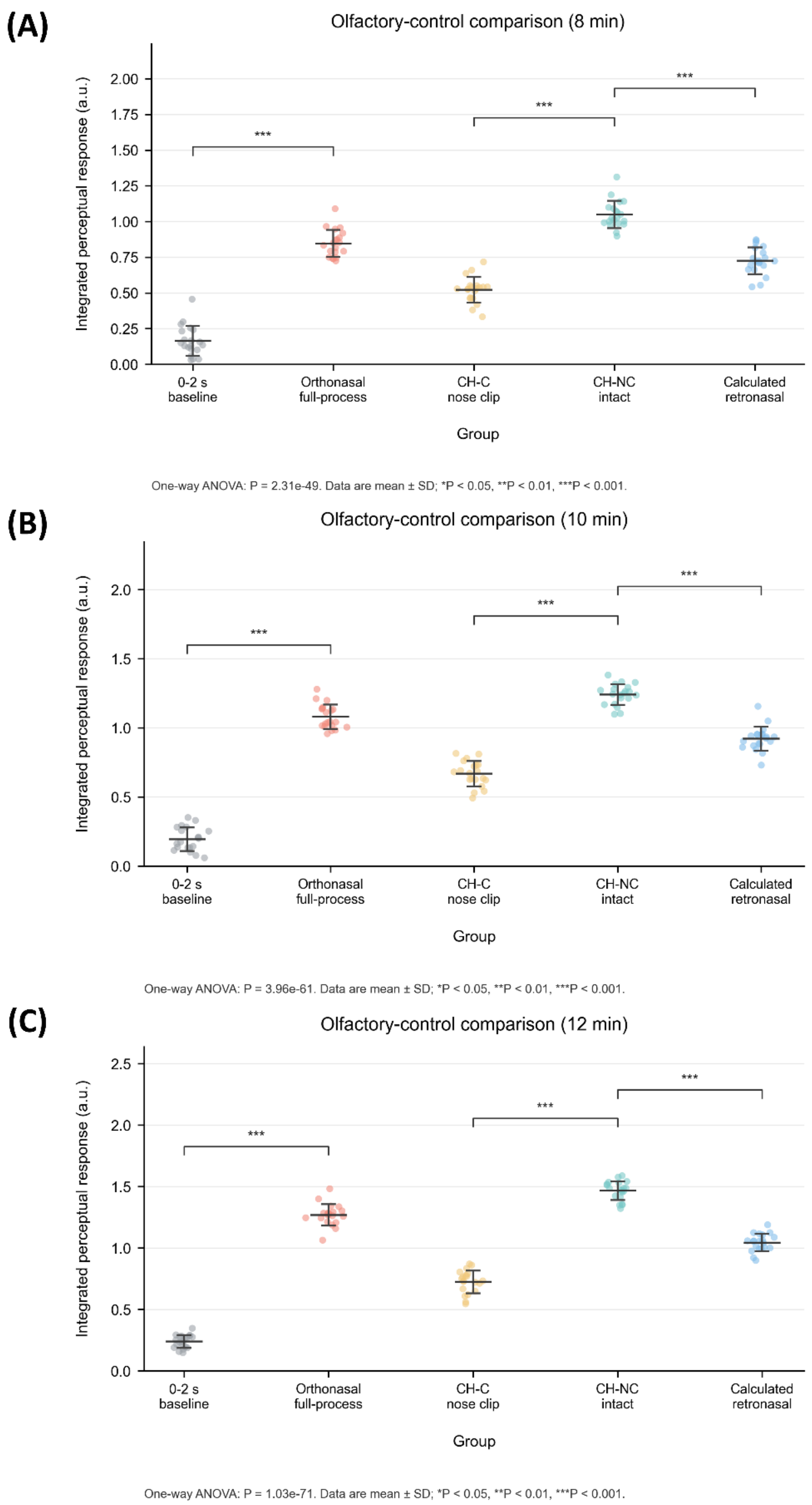

FIGURE S4. Integrated perceptual EEG responses under different olfactory control conditions for grilled lamb skewers with varying grilling durations. (A) 8 min-grilled lamb skewers; (B)

10 min-grilled lamb skewers; (C) 12 min-grilled lamb skewers. Five conditions are compared: 0–2 s resting baseline, full-process orthonasal olfaction, chewing with nasal occlusion (CH-C), intact chewing (CH-NC), and the calculated pure retronasal aroma response.

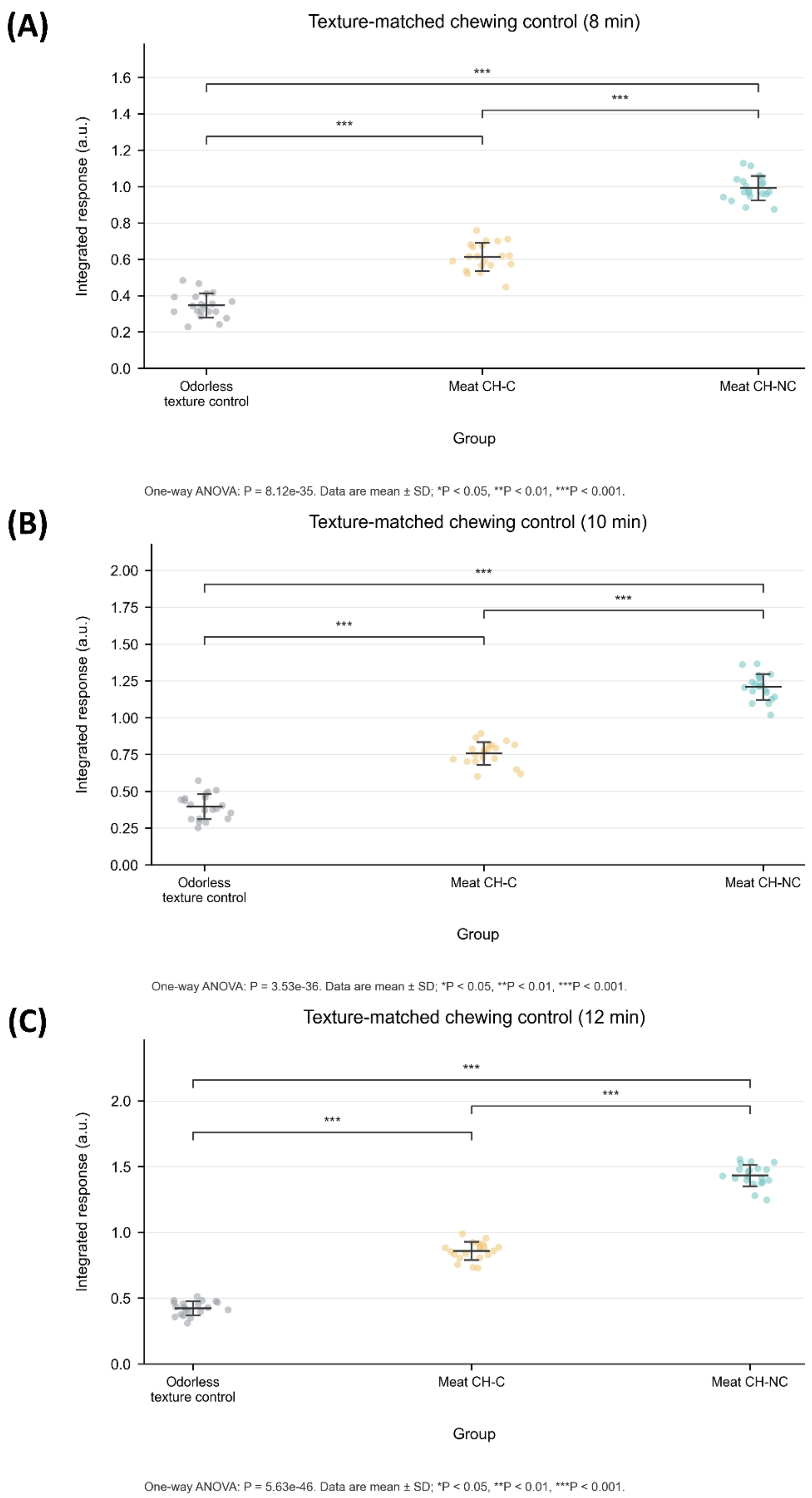

FIGURE S5. Integrated perceptual EEG responses from texture-matched control experiments to isolate chewing-induced oral somatosensory effects. (A) 8 min-grilled lamb skewers; (B) 10

min-grilled lamb skewers; (C) 12 min-grilled lamb skewers. Three groups are compared: odorless texture-matched matrix, lamb skewers chewing with nasal occlusion (Meat CH-C), and intact lamb skewers chewing (Meat CH-NC).

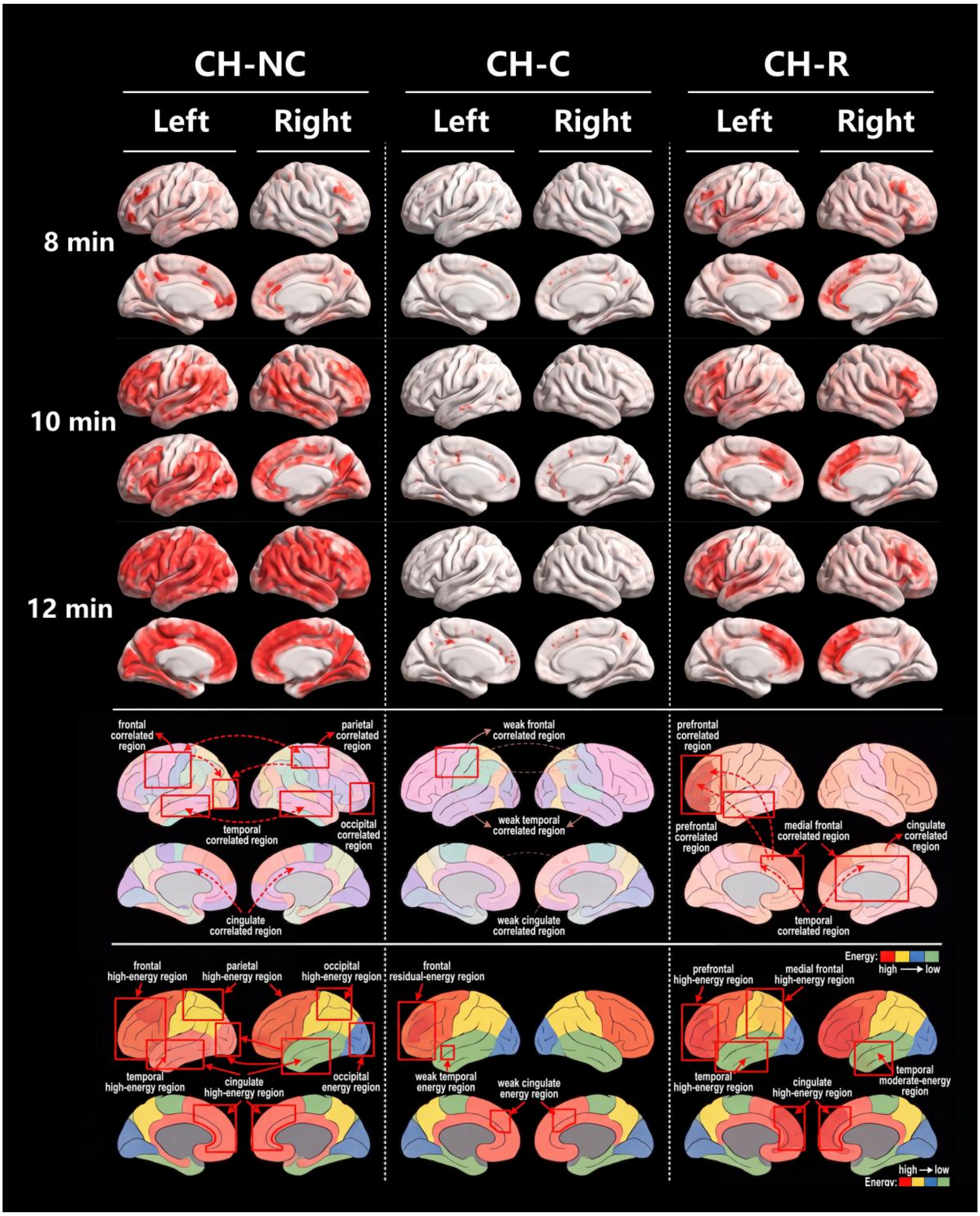

FIGURE S6. Cortical activation patterns of retronasal aroma perception revealed by sLORETA-based EEG source localization. The cortical activation maps display three grilling durations (8, 10, and 12 minutes) of lamb skewers under three experimental conditions:

complete chewing (CH-NC), chewing with nasal closure (CH-C), and isolated pure retronasal aroma response (CH-R).

(A)

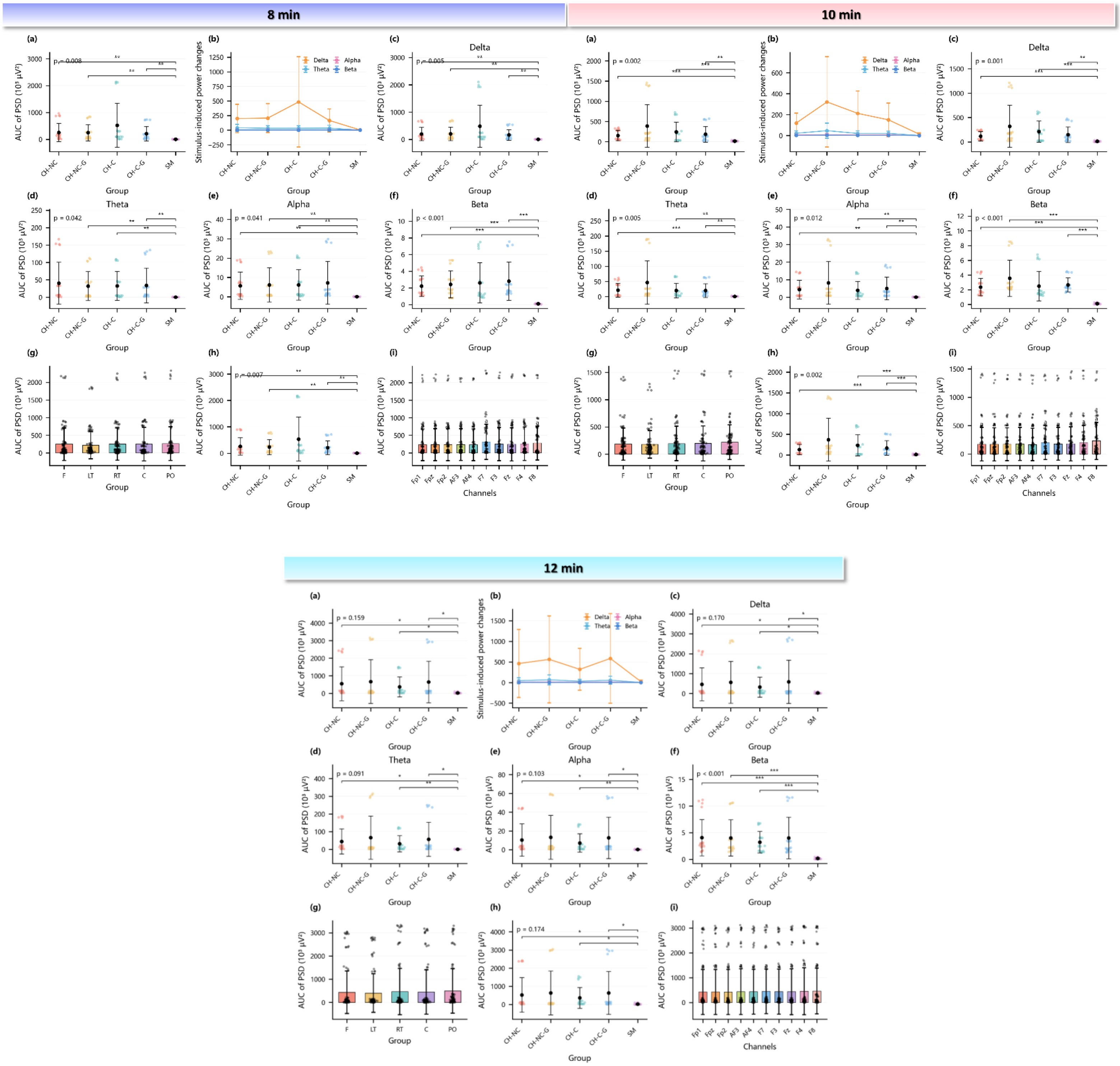

8 min
10 min
12 min
(a)
(b)
(c)
(d)
(e)
(f)
(g)
(h)
(i)
Delta
Theta
Alpha
Beta
AUC of PSD (10³ µV²)
Stimulus-induced power changes
Group
Channels
CH-NC
CH-NC-G
CH-C
CH-C-G
SM
F
LT
RT
C
PO
Fp1
Fpz
Fp2
AF3
AF4
F7
F3
Fz
F4
F8

(B)

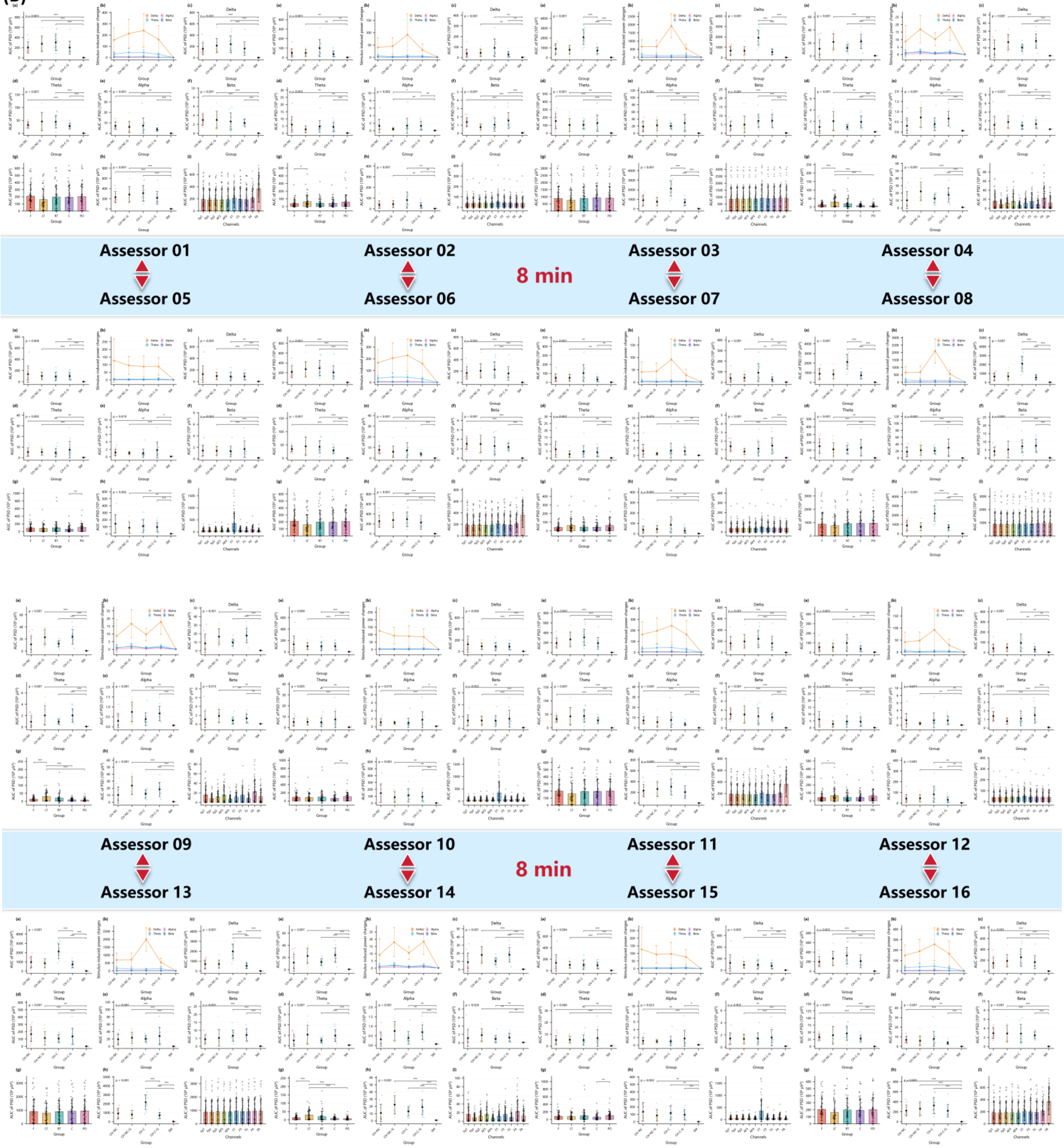

Assessor 01
Assessor 05
Assessor 02
Assessor 06
8 min
Assessor 03
Assessor 07
Assessor 04
Assessor 08
Assessor 09
Assessor 13
Assessor 10
Assessor 14
8 min
Assessor 11
Assessor 15
Assessor 12
Assessor 16

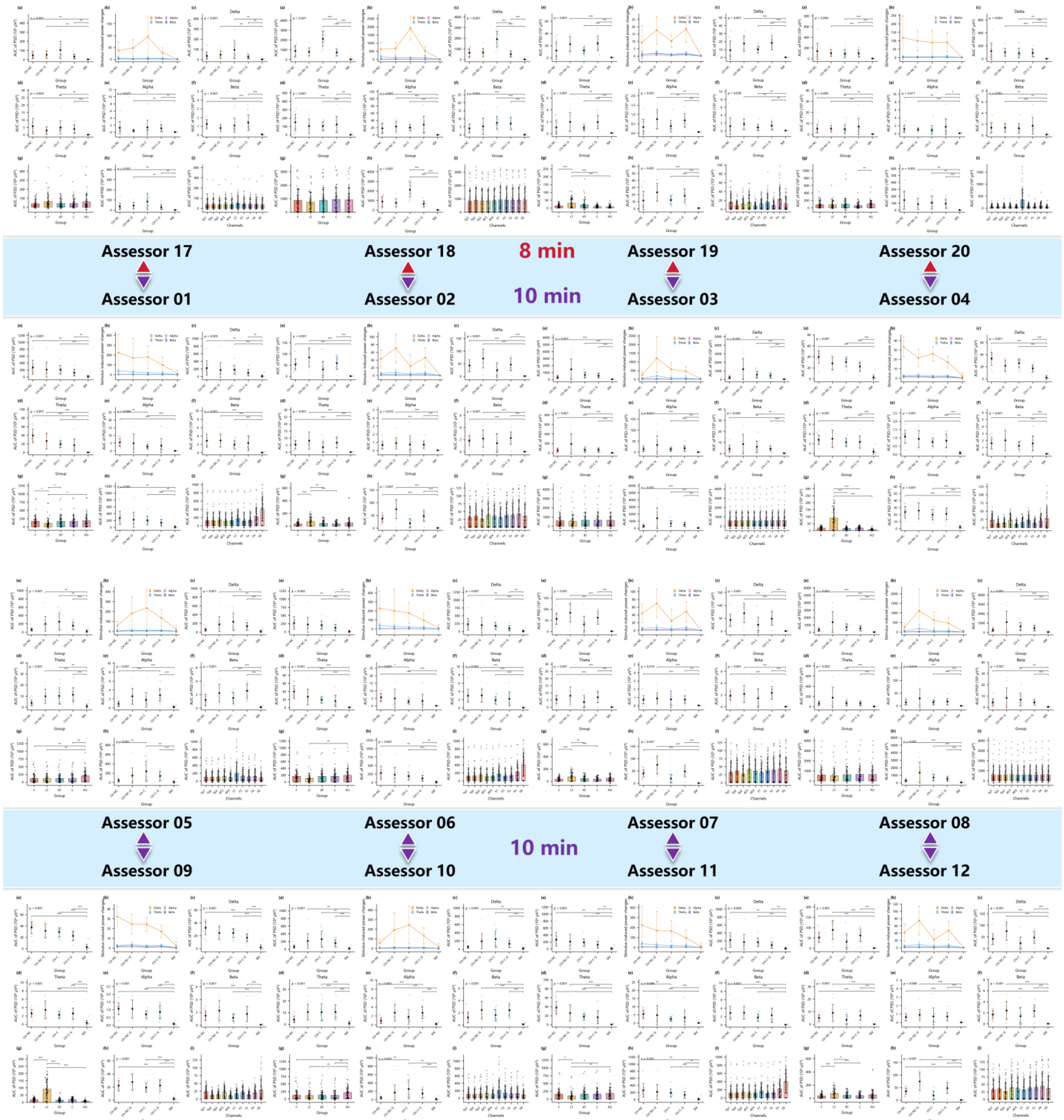

Assessor 17
Assessor 01
Assessor 18
Assessor 02
8 min
10 min
Assessor 19
Assessor 03
Assessor 20
Assessor 04
Assessor 05
Assessor 09
Assessor 06
Assessor 10
10 min
Assessor 07
Assessor 11
Assessor 08
Assessor 12

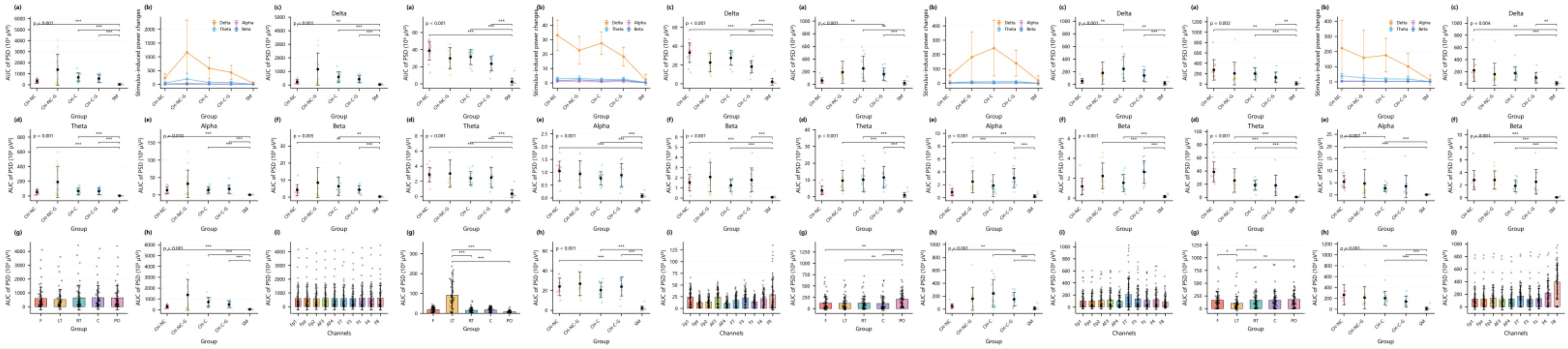

Assessor 13 Assessor 14 Assessor 15 Assessor 16

10 min

Assessor 17 Assessor 18 Assessor 19 Assessor 20

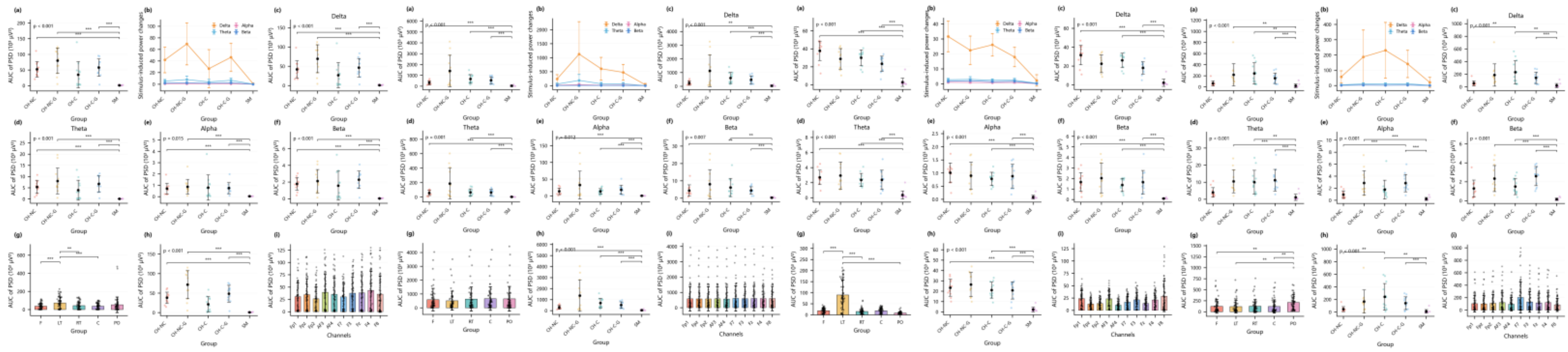

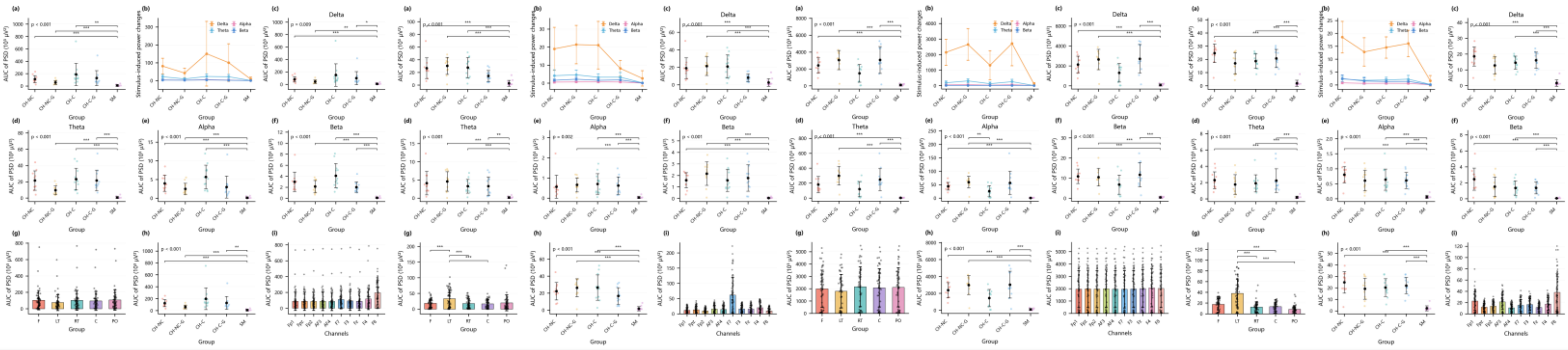

Assessor 01 Assessor 02 Assessor 03 Assessor 04

12 min

Assessor 05 Assessor 06 Assessor 07 Assessor 08

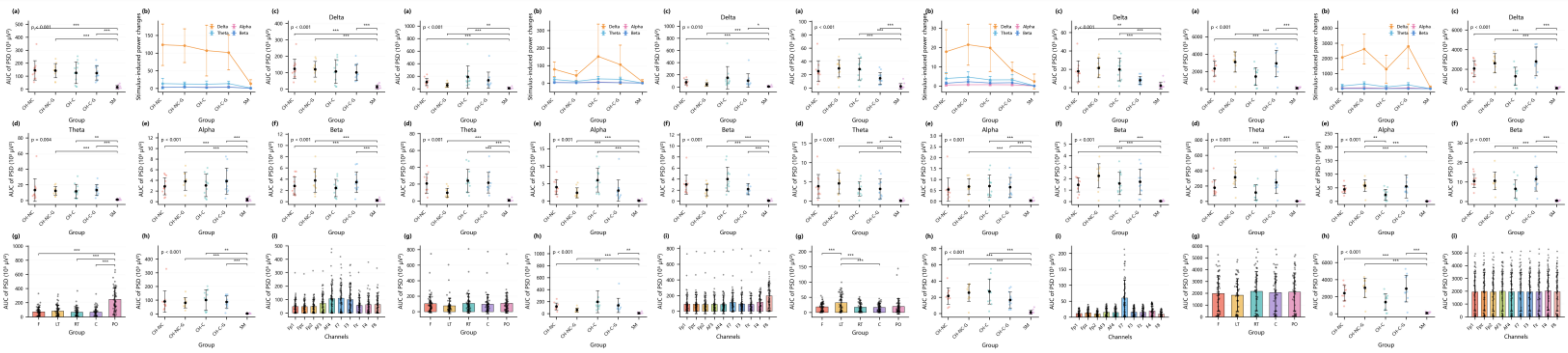

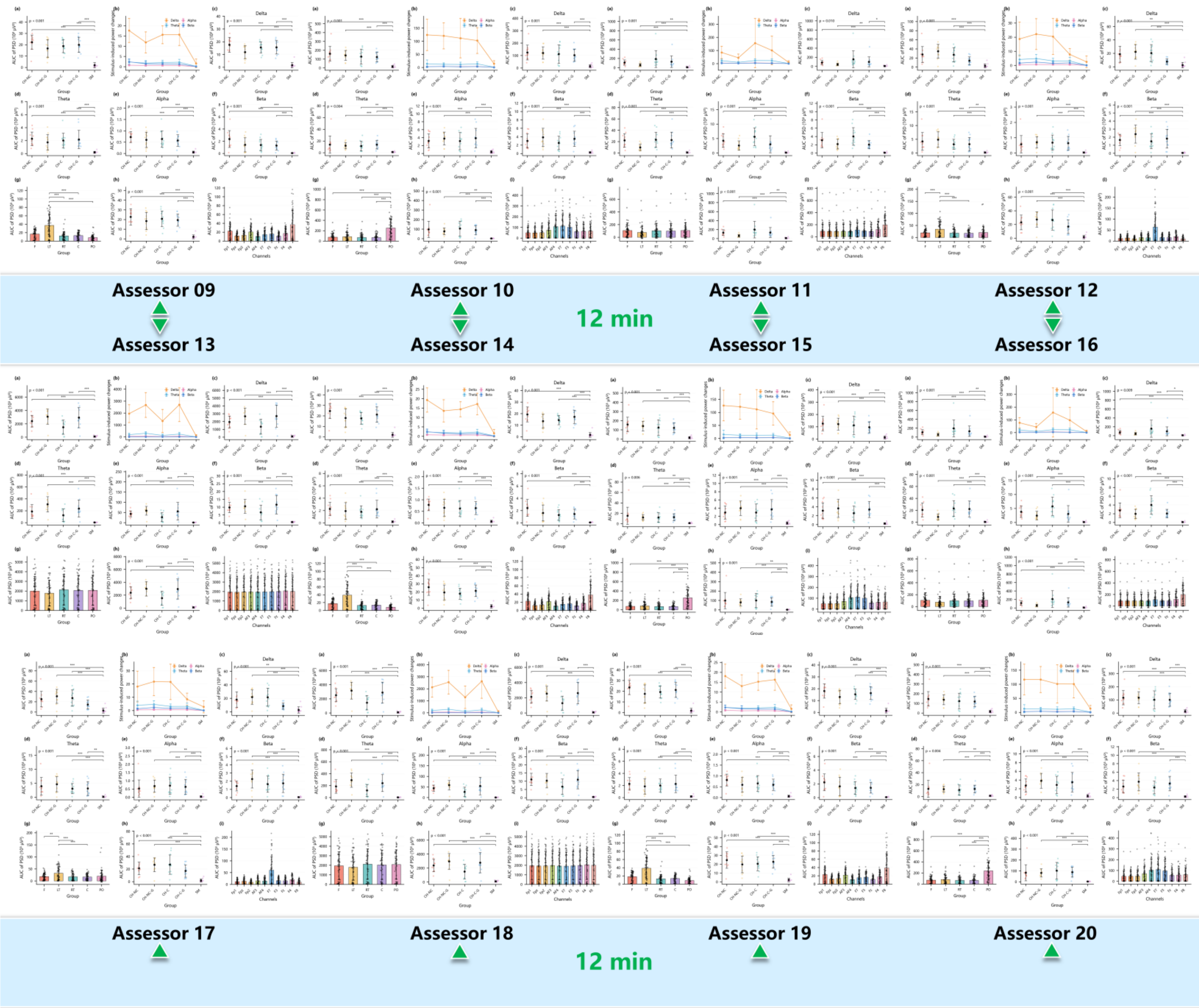

FIGURE S7. Subject-level and group-averaged PSD area under the curve across frequency bands, brain regions and channels for different grilling durations. (A) Group-averaged PSD-AUC values with standard deviation for delta, theta, alpha and beta bands under different experimental conditions. Results are presented at both regional (frontal, temporal, central, parieto-occipital) and single-channel levels for 8, 10 and 12 min grilled lamb skewers. (B) Individual data points from 20 participants, demonstrating inter-individual variability and the consistent direction of difference between the CH-NC and CH-C conditions.

(A)

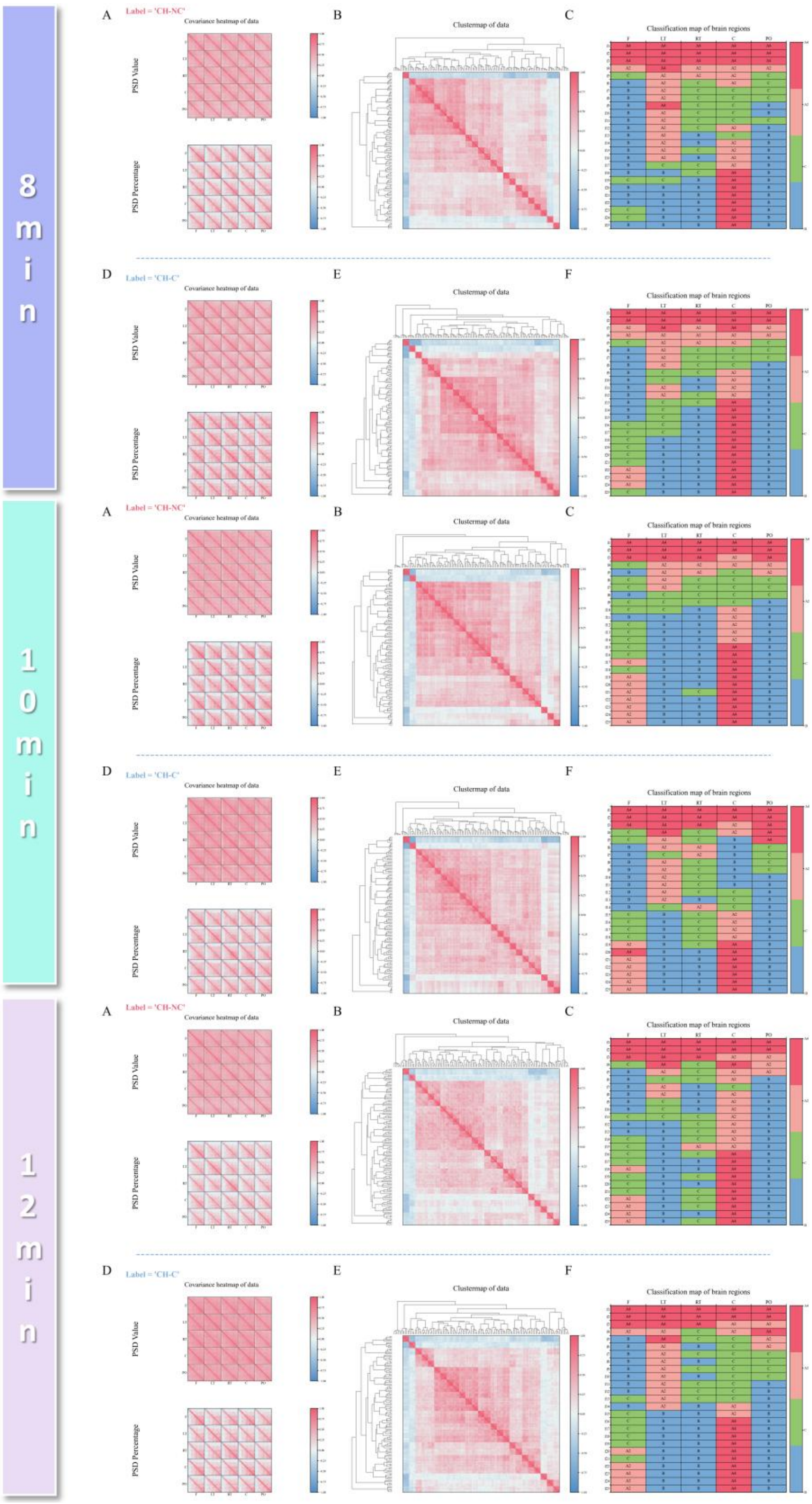
8 min
10 min
12 min
A
Label = 'CH-NC'
Covariance heatmap of data
PSD Value
PSD Percentage
B
Clustermap of data
C
Classification map of brain regions
D
Label = 'CH-C'
E
F

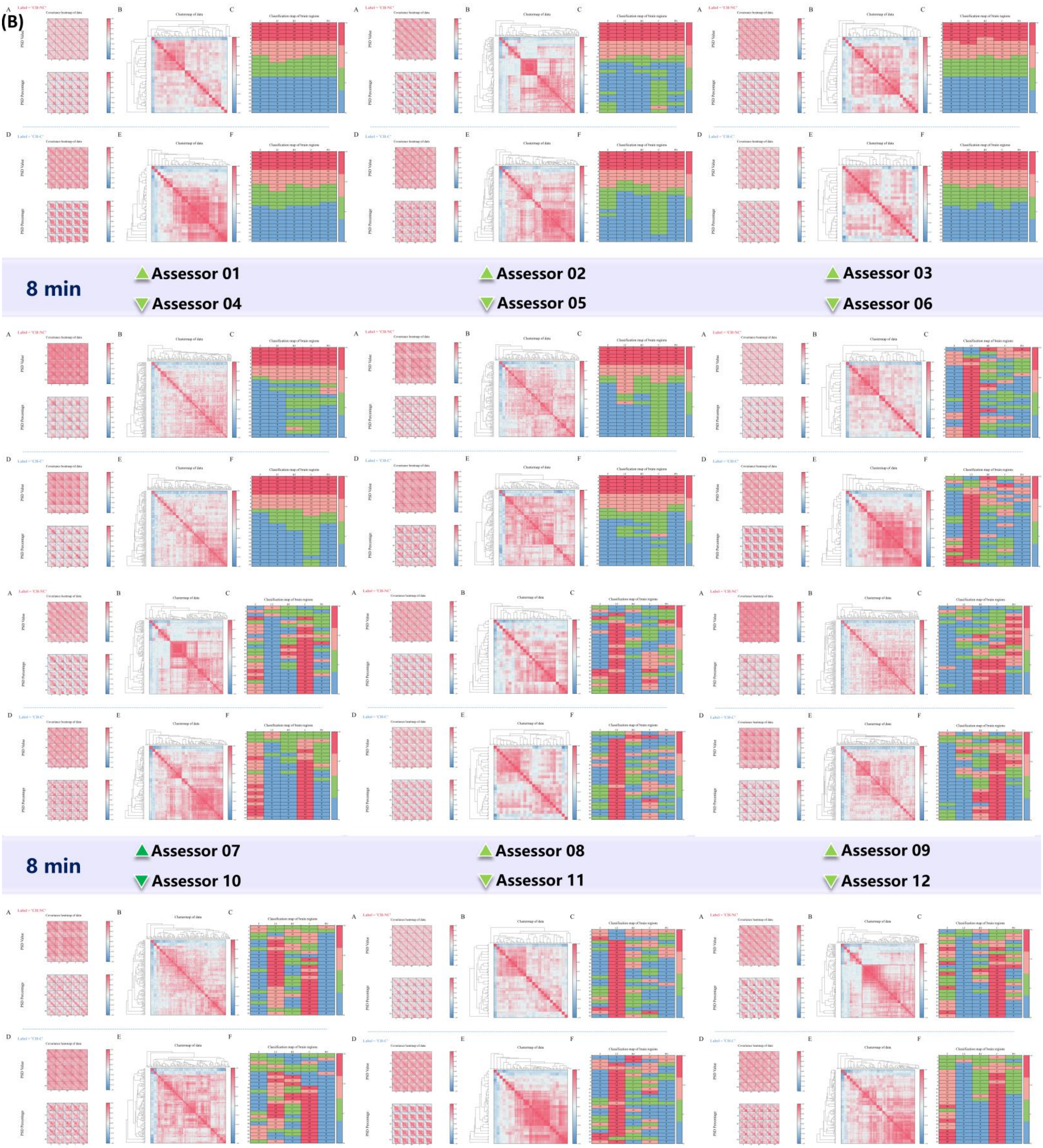
(B)
8 min
Assessor 01
Assessor 02
Assessor 03
Assessor 04
Assessor 05
Assessor 06
8 min
Assessor 07
Assessor 08
Assessor 09
Assessor 10
Assessor 11
Assessor 12

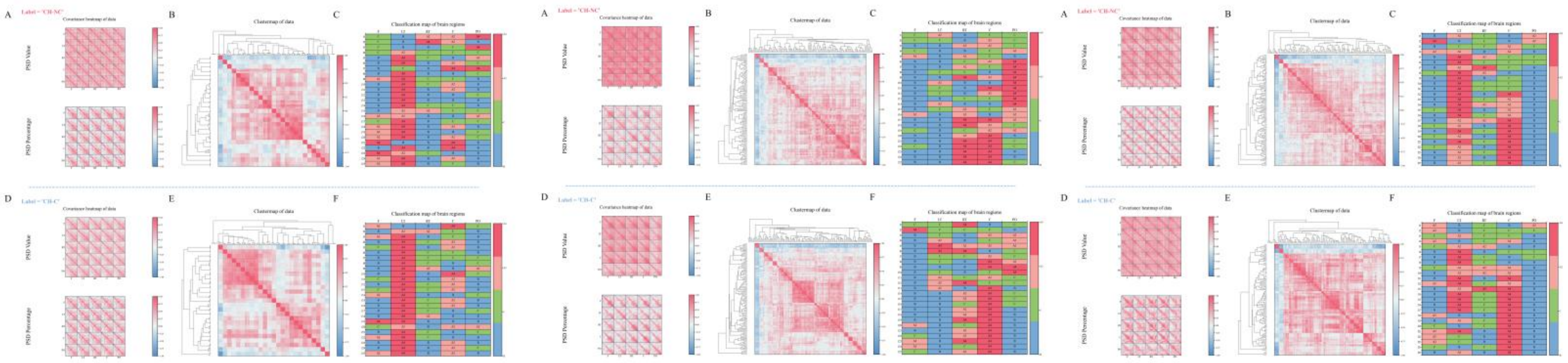

8 min

▲ Assessor 13 ▲ Assessor 14 ▲ Assessor 15

▼ Assessor 16 ▼ Assessor 17 ▼ Assessor 18

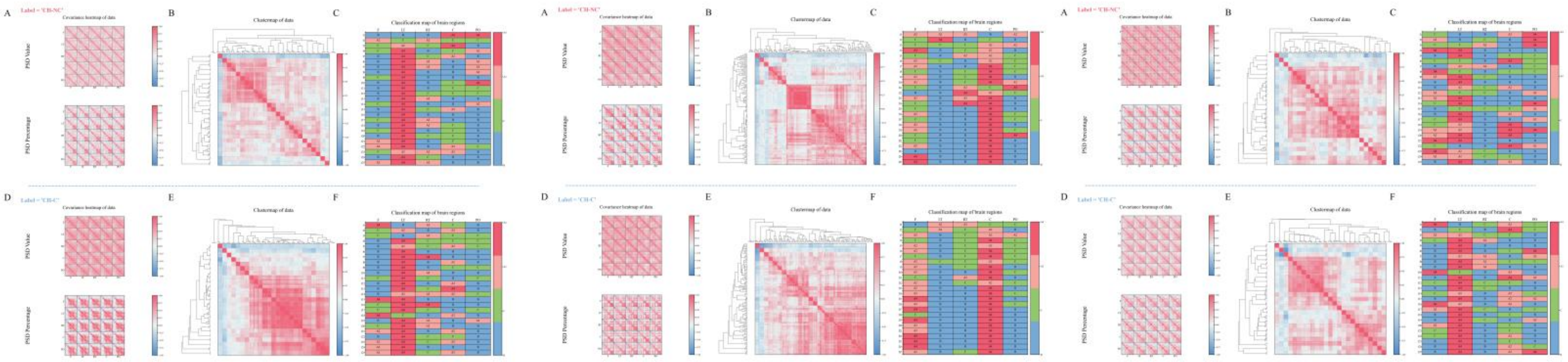

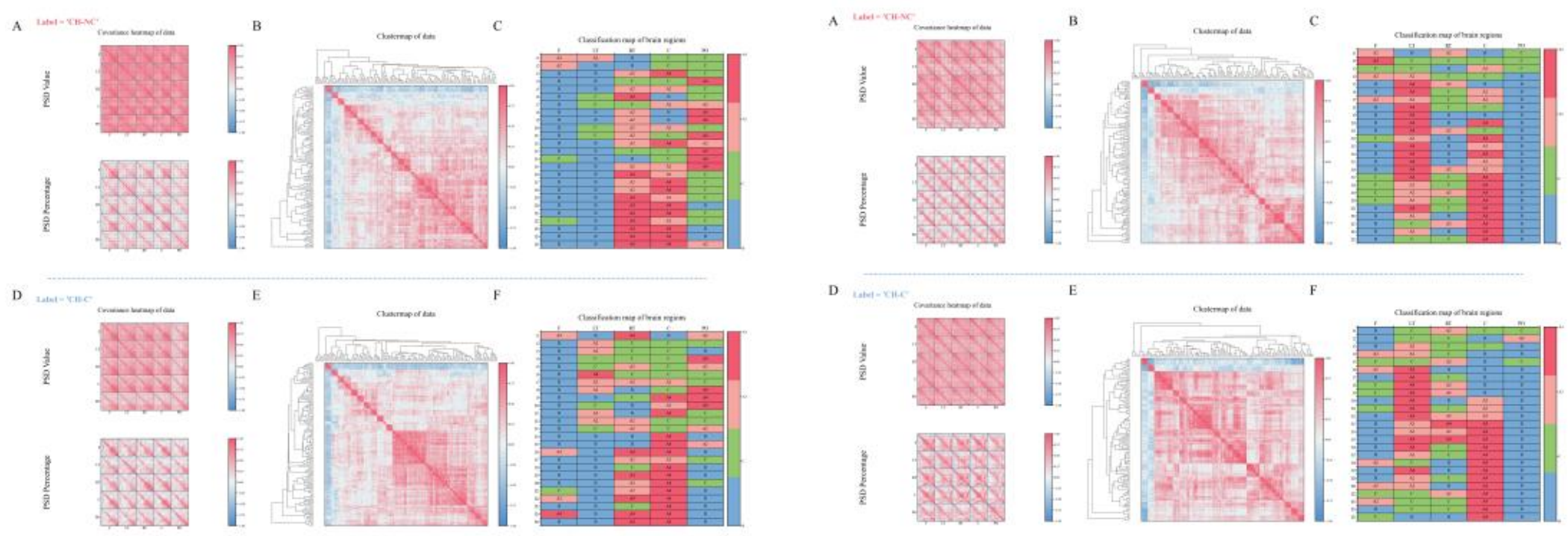

8 min ▲ Assessor 19 ▲ Assessor 20

10 min ▽ Assessor 01 ▽ Assessor 02 ▽ Assessor 03

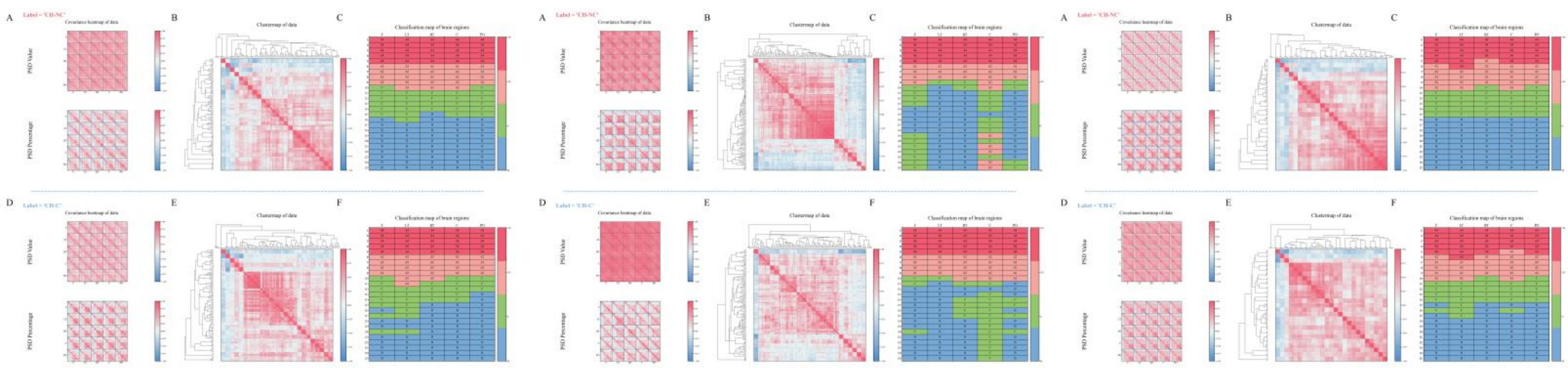

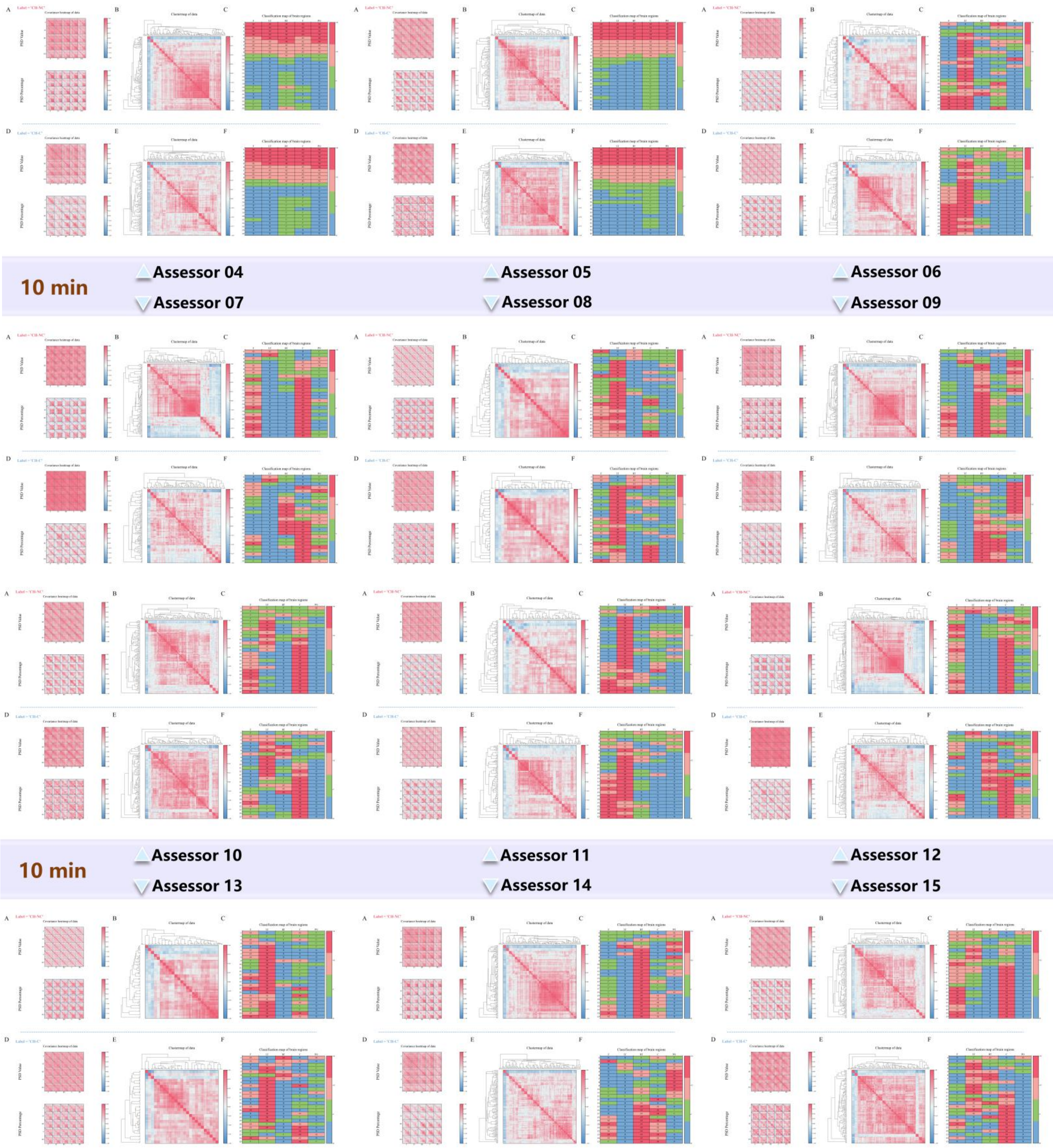
10 min
Assessor 04
Assessor 07
Assessor 05
Assessor 08
Assessor 06
Assessor 09
10 min
Assessor 10
Assessor 13
Assessor 11
Assessor 14
Assessor 12
Assessor 15

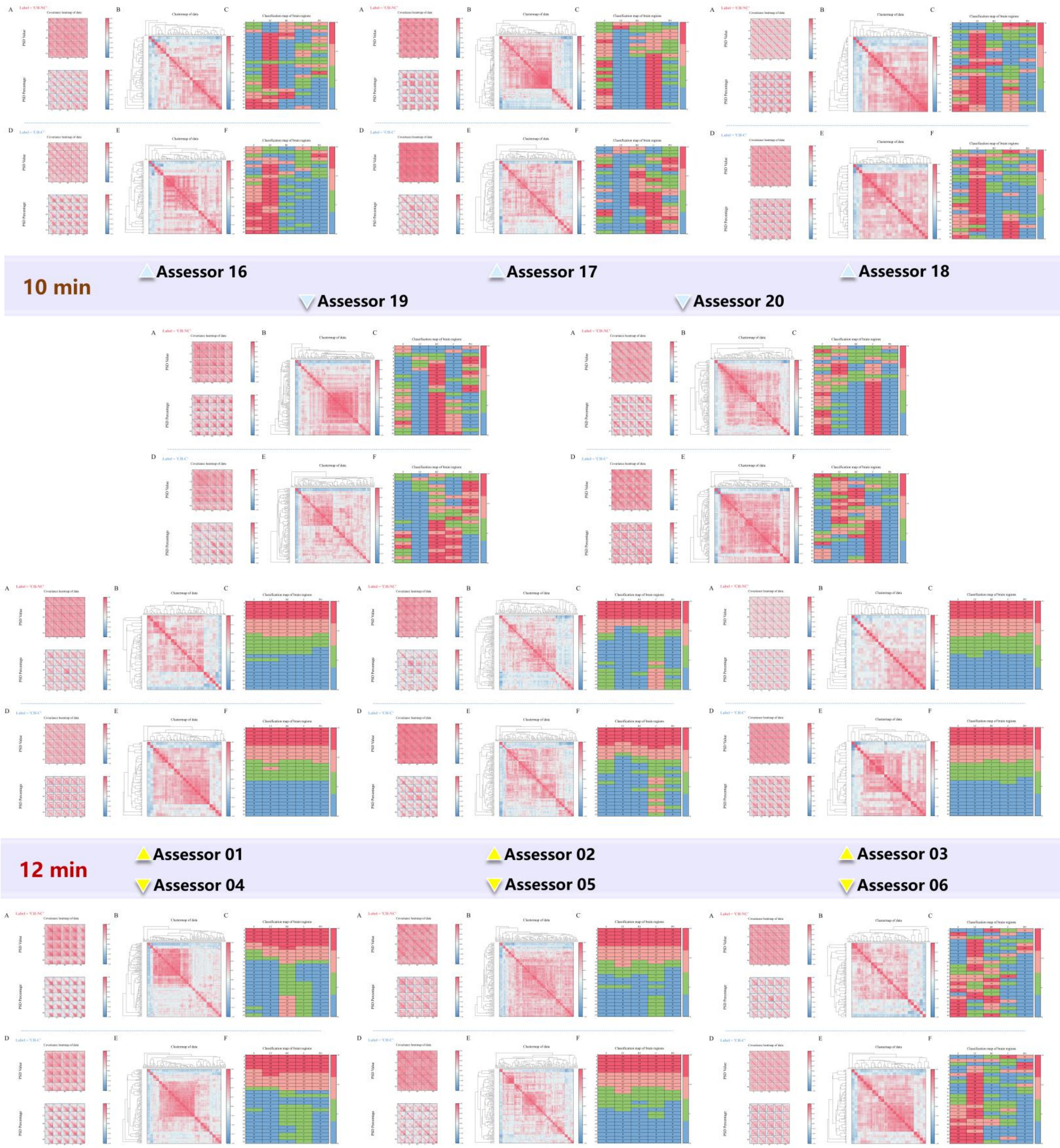
Assessor 16
Assessor 17
Assessor 18
10 min
Assessor 19
Assessor 20
Assessor 01
Assessor 02
Assessor 03
12 min
Assessor 04
Assessor 05
Assessor 06

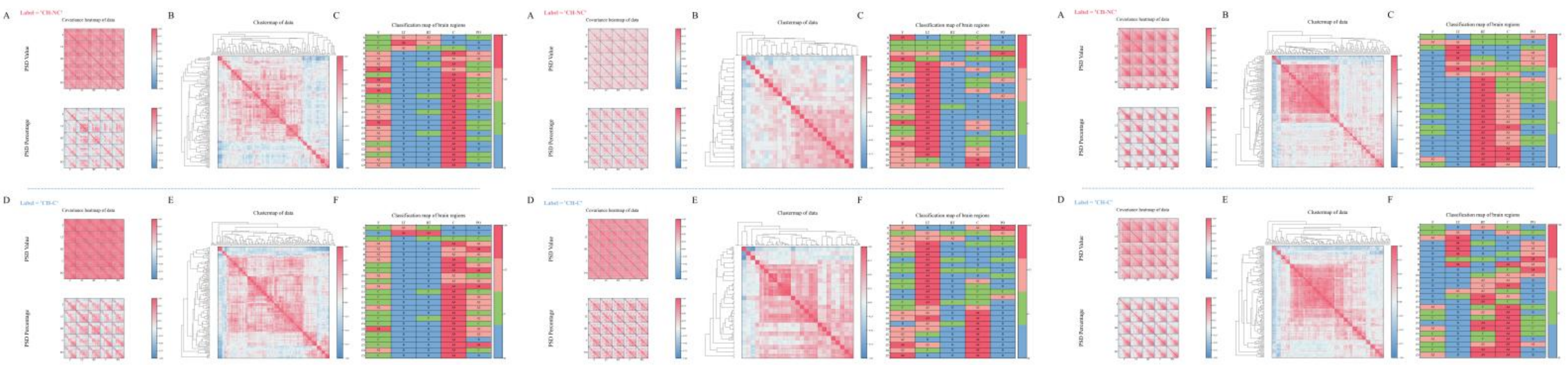

12 min

▲Assessor 07 ▲Assessor 08 ▲Assessor 09

▼Assessor 10 ▼Assessor 11 ▼Assessor 12

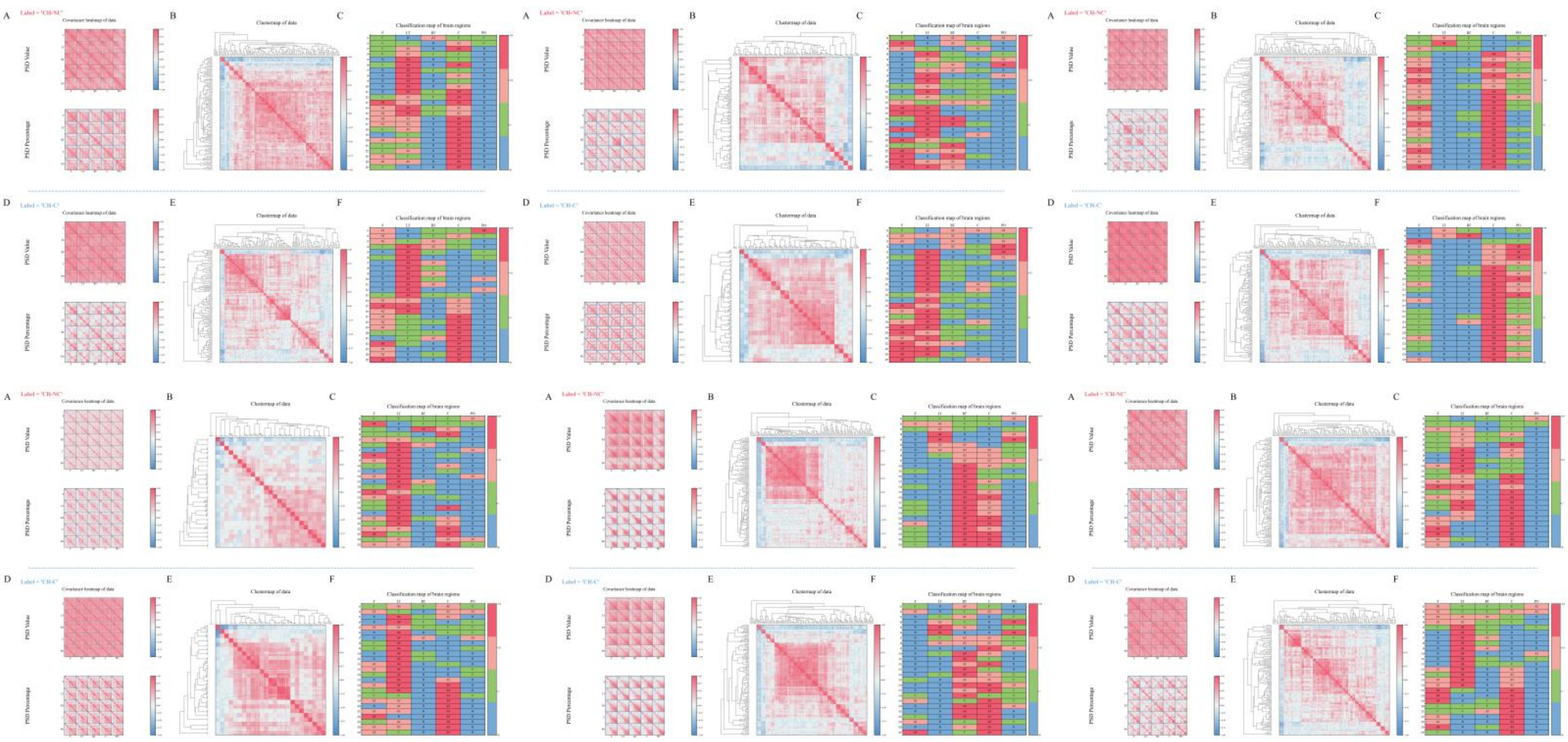

12 min

▲Assessor 13 ▲Assessor 14 ▲Assessor 15

▼Assessor 16 ▼Assessor 17 ▼Assessor 18

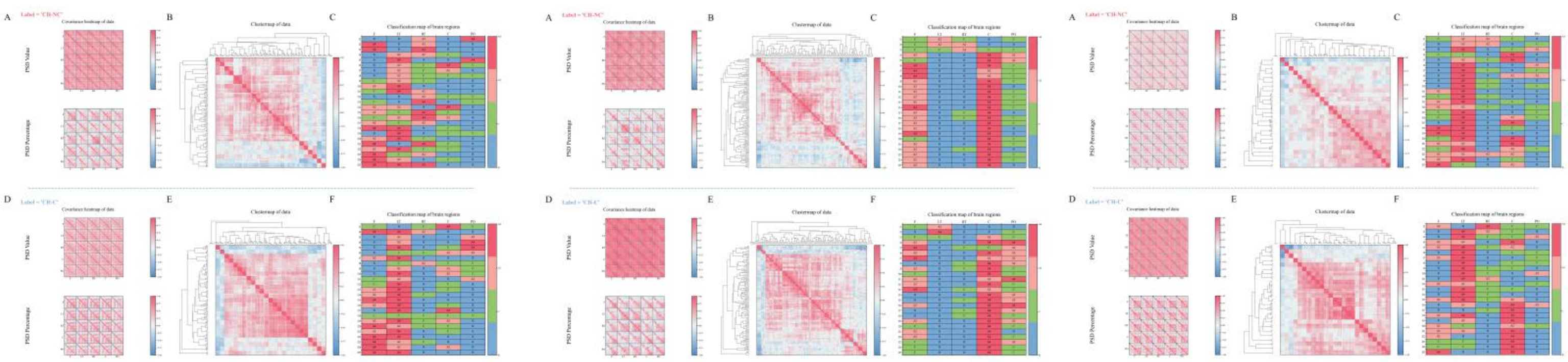

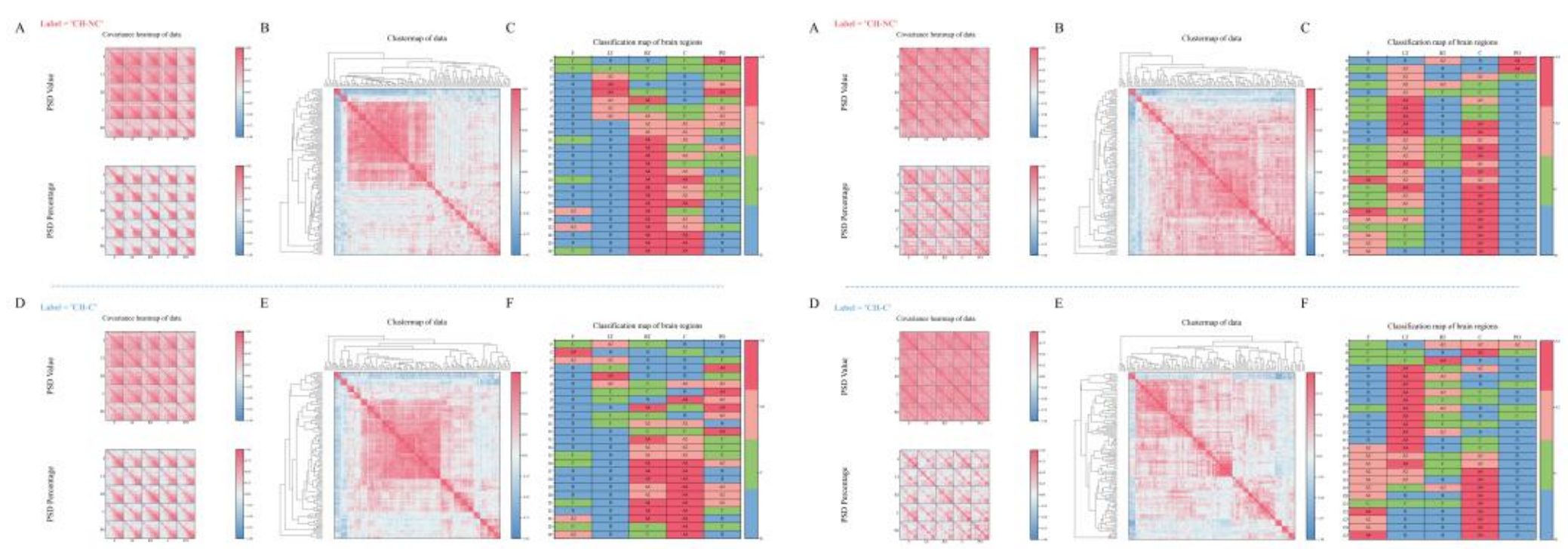

Assessor 19 Assessor 20

12 min

FIGURE S8. Regional power spectral density profiles under three experimental conditions across grilling durations. (A) Group-averaged PSD curves across 20 participants in five brain regions (frontal, left temporal, right temporal, central, parieto-occipital) under the CH-C, CH-NC and CH-R conditions. Results are shown separately for 8, 10 and 12 min of grilling, with shaded bands representing the standard error of the mean. (B) Overlaid individual PSD traces from all participants, illustrating the consistency of the low-frequency enhancement pattern induced by retronasal aroma input.

(A)

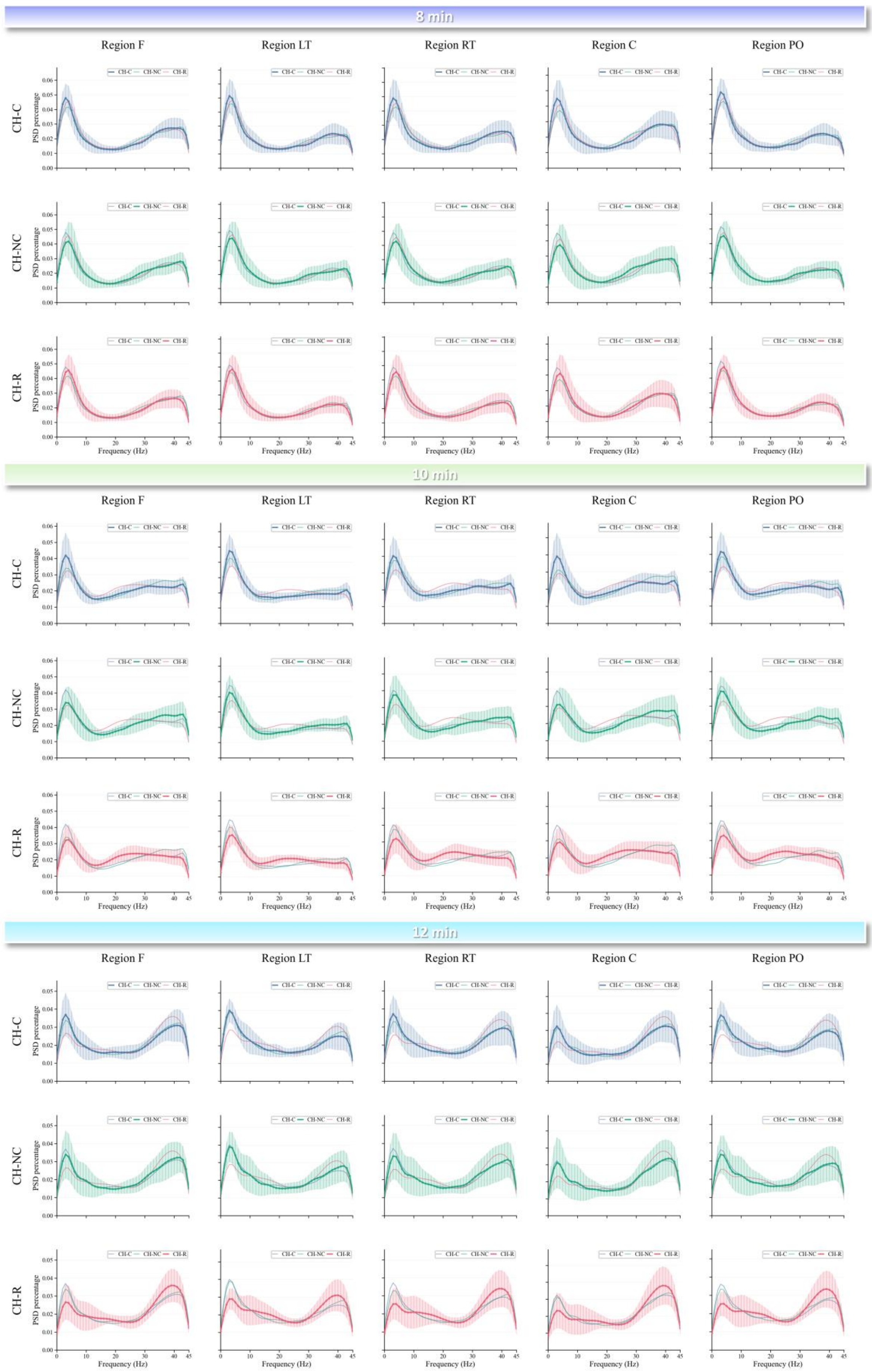

8 min
Region F
Region LT
Region RT
Region C
Region PO
CH-C
CH-NC
CH-R
PSD percentage
Frequency (Hz)
10 min
12 min

(B)

8 min samples assessors 1-10

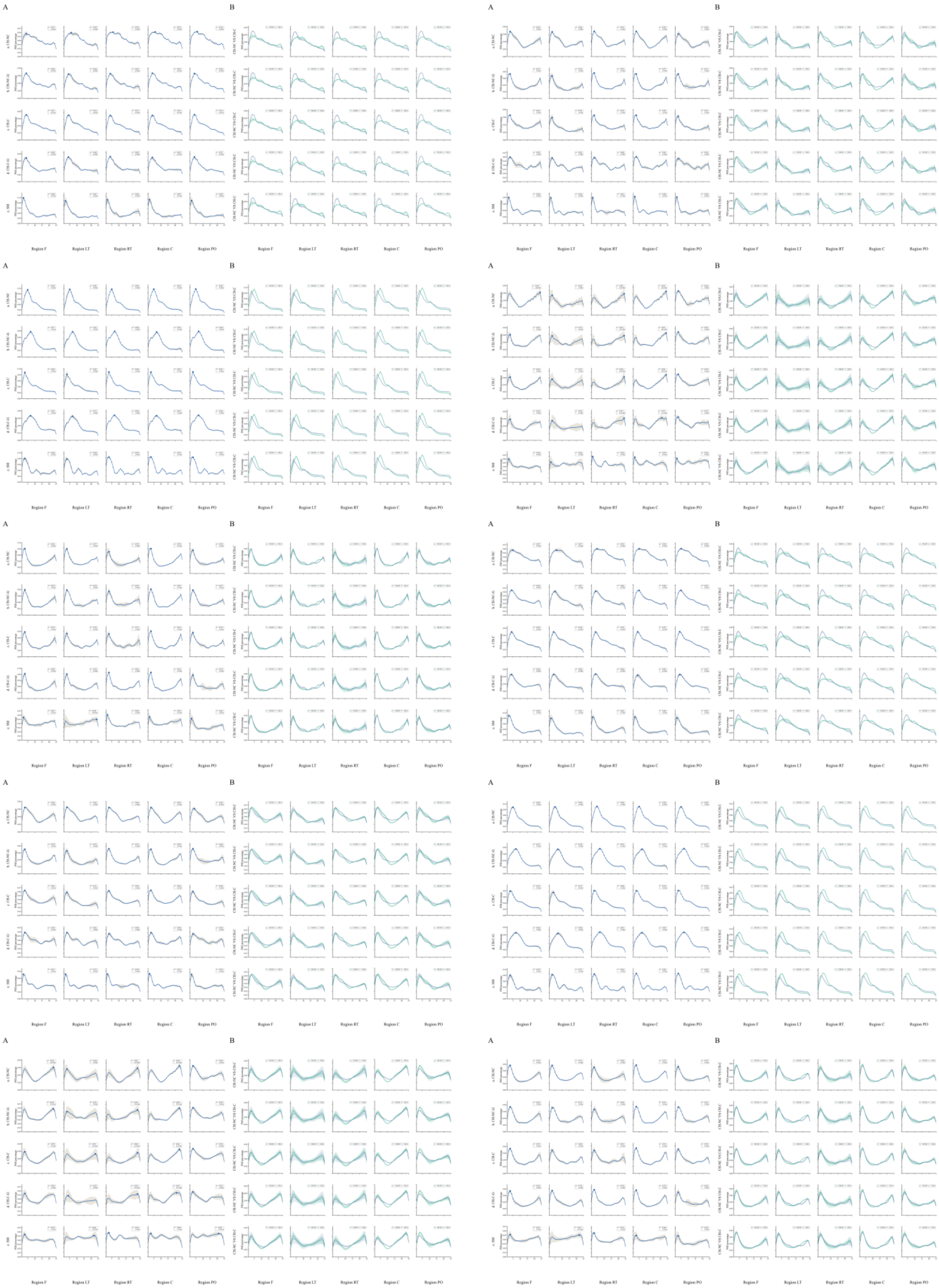

8 min samples assessors 11-20

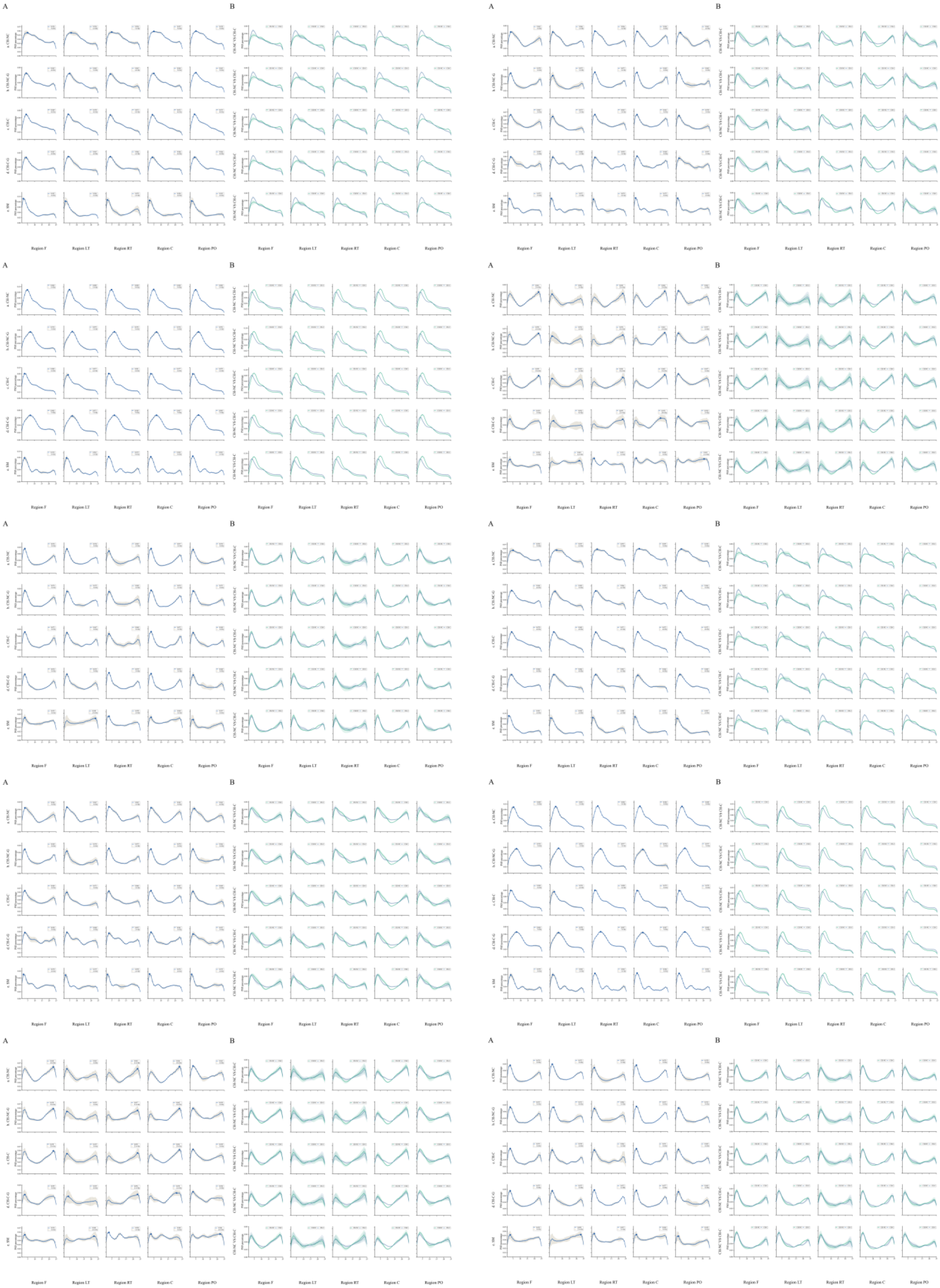

10 min samples assessors 1-10

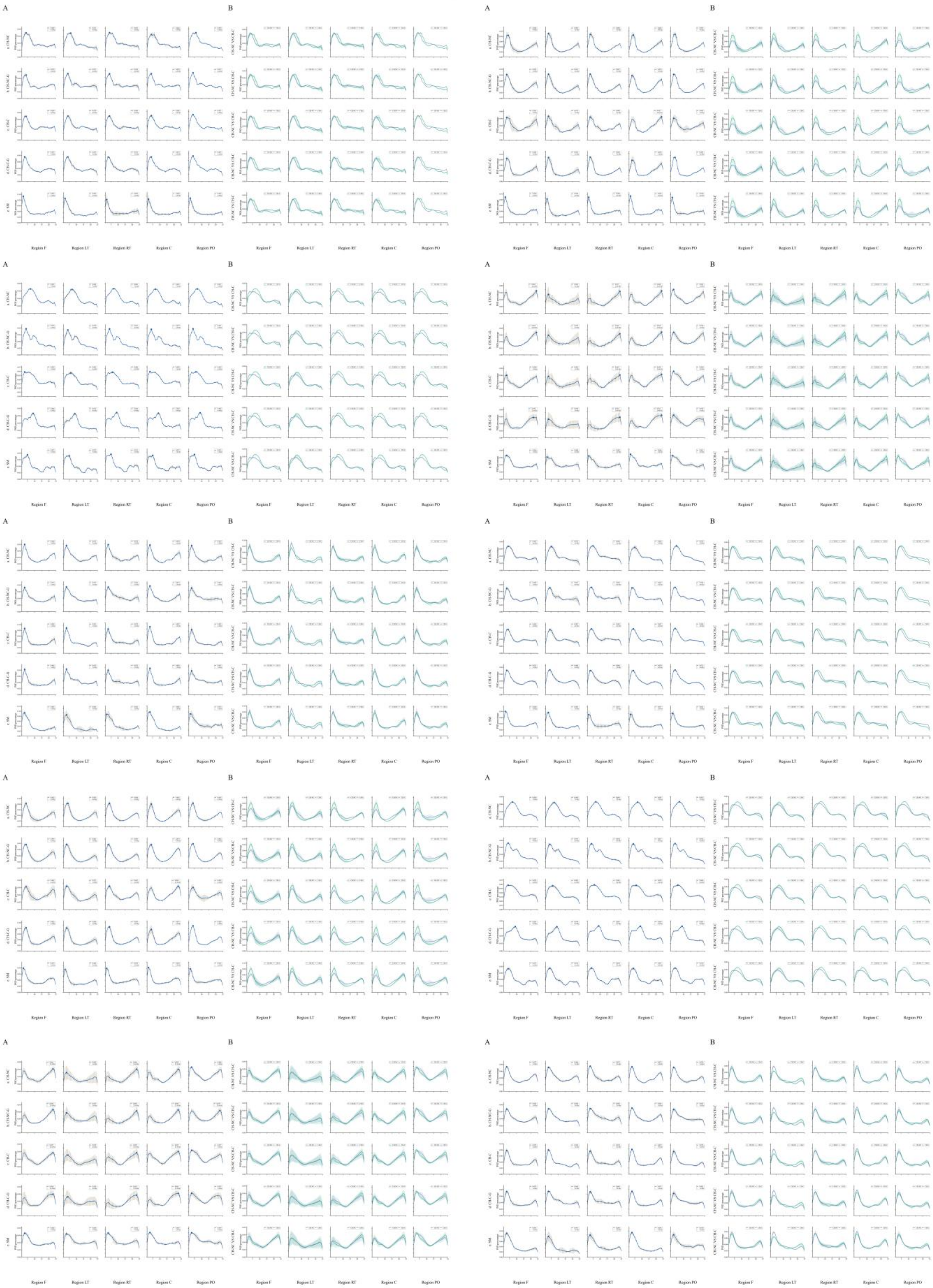

10 min samples assessors 11-20

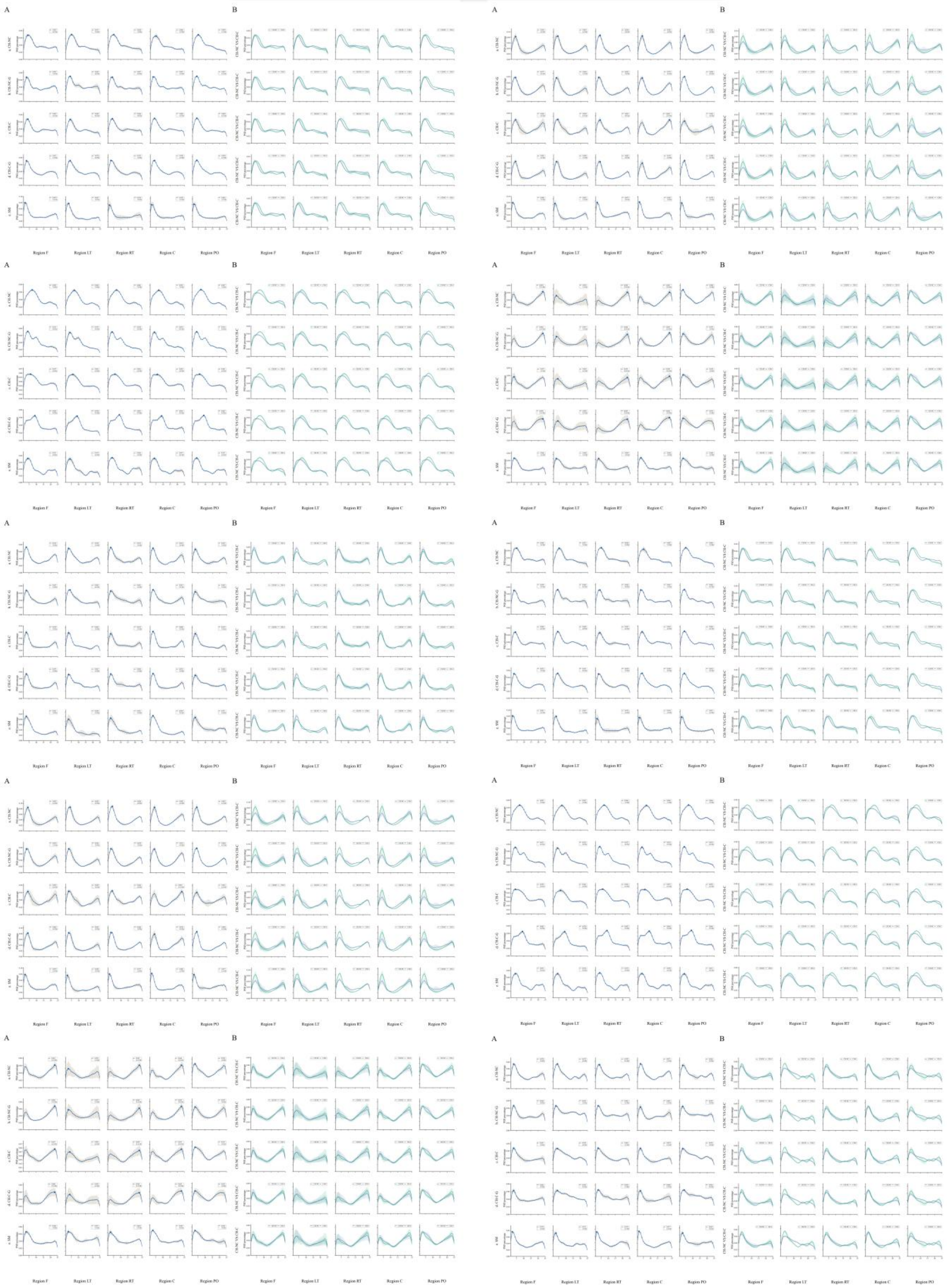

12 min samples assessors 1-10

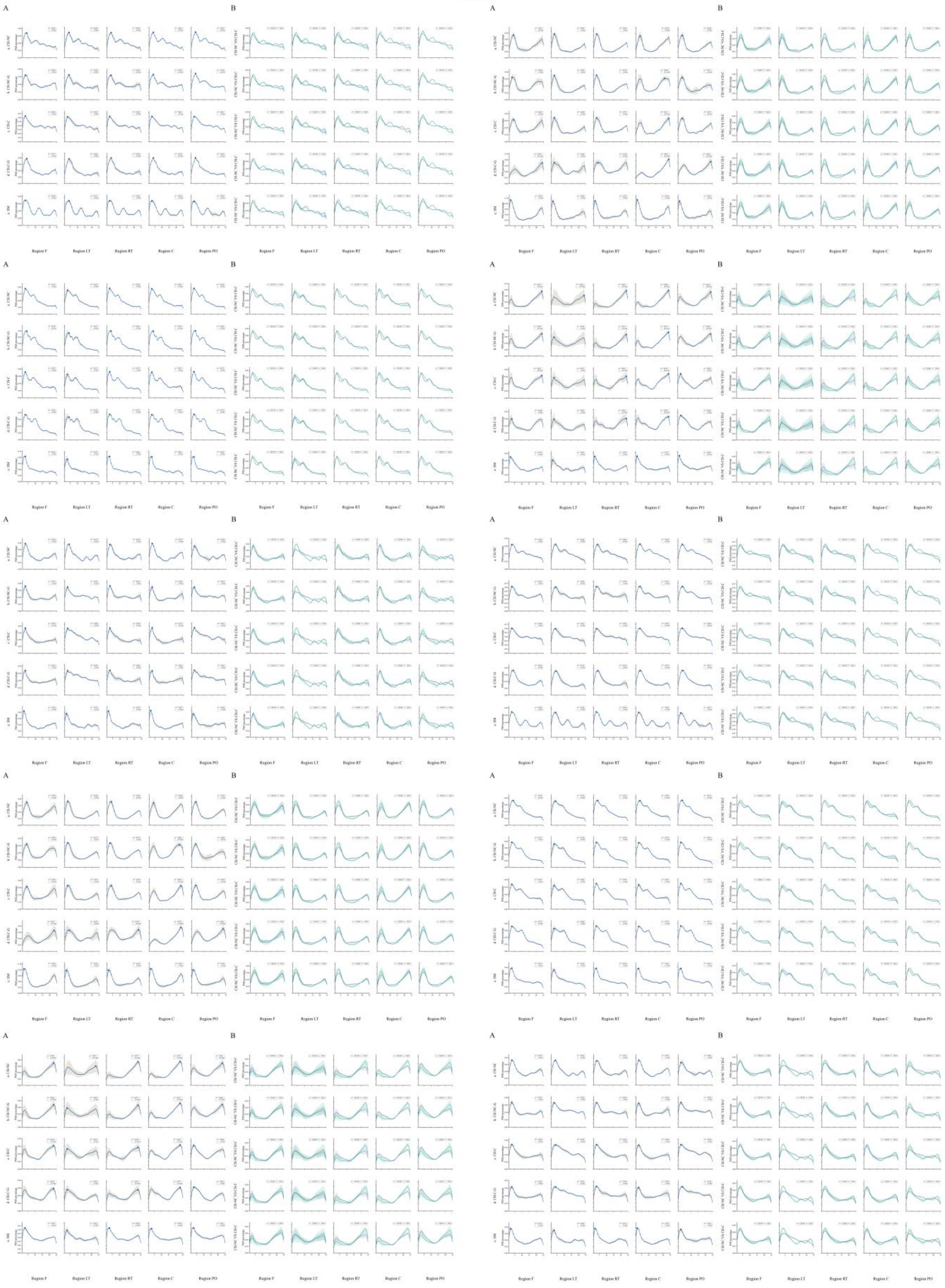

12 min samples assessors 11-20

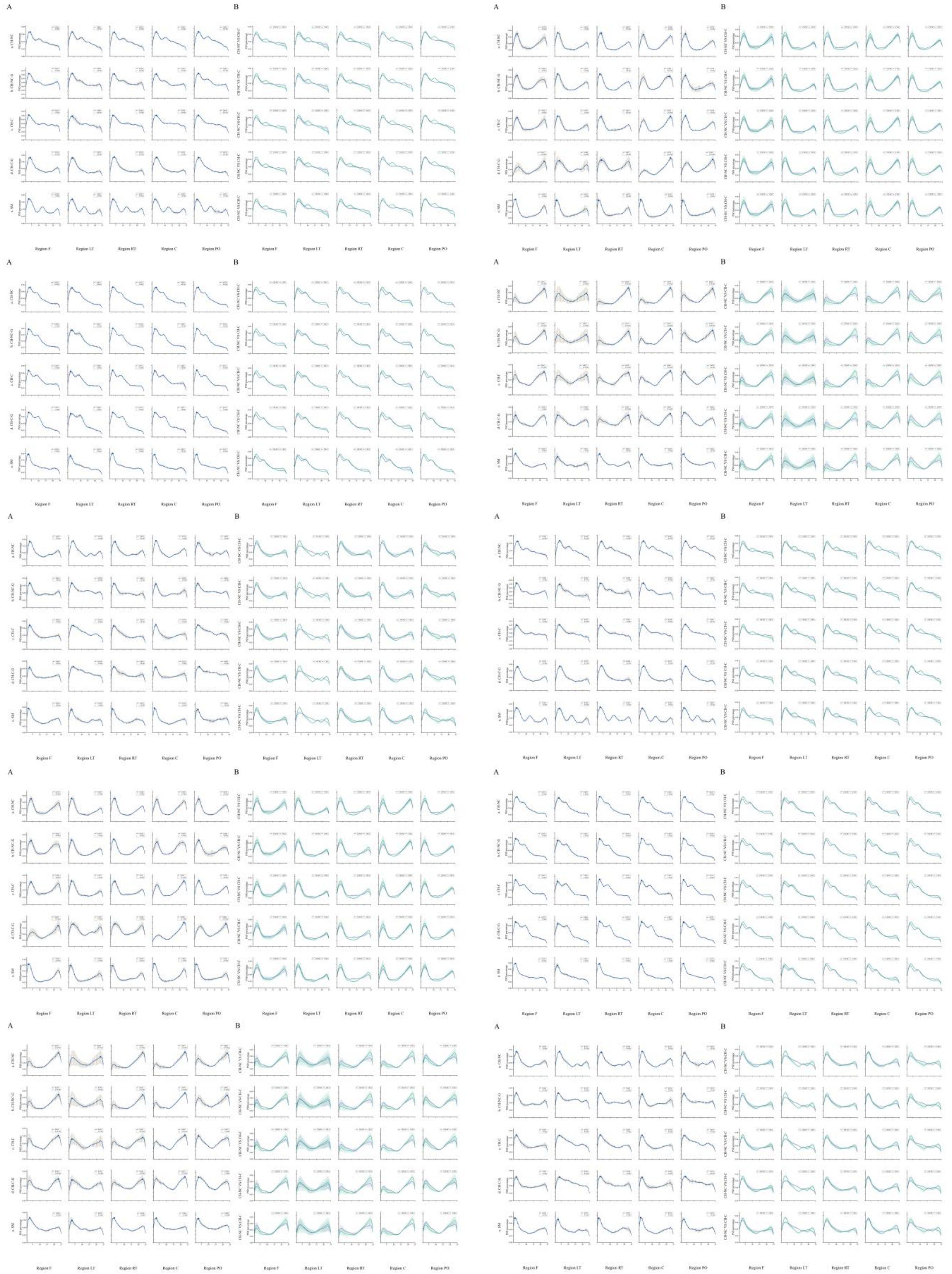

FIGURE S9. Inter-channel covariance patterns and spatial clustering of EEG signals across CH-NC, CH-C and CH-R conditions. (A) Group-averaged inter-channel covariance heatmaps, hierarchical clustering dendrograms and brain region parcellation maps for 8, 10 and 12 min grilled lamb skewers, comparing the spatial organization of functional connectivity under intact chewing (CH-NC), nasal occlusion (CH-C) and isolated retronasal aroma (CH-R) conditions. (B) Individual-level covariance patterns from 20 participants, demonstrating the inter-individual consistency of connectivity network differences induced by retronasal aroma input.

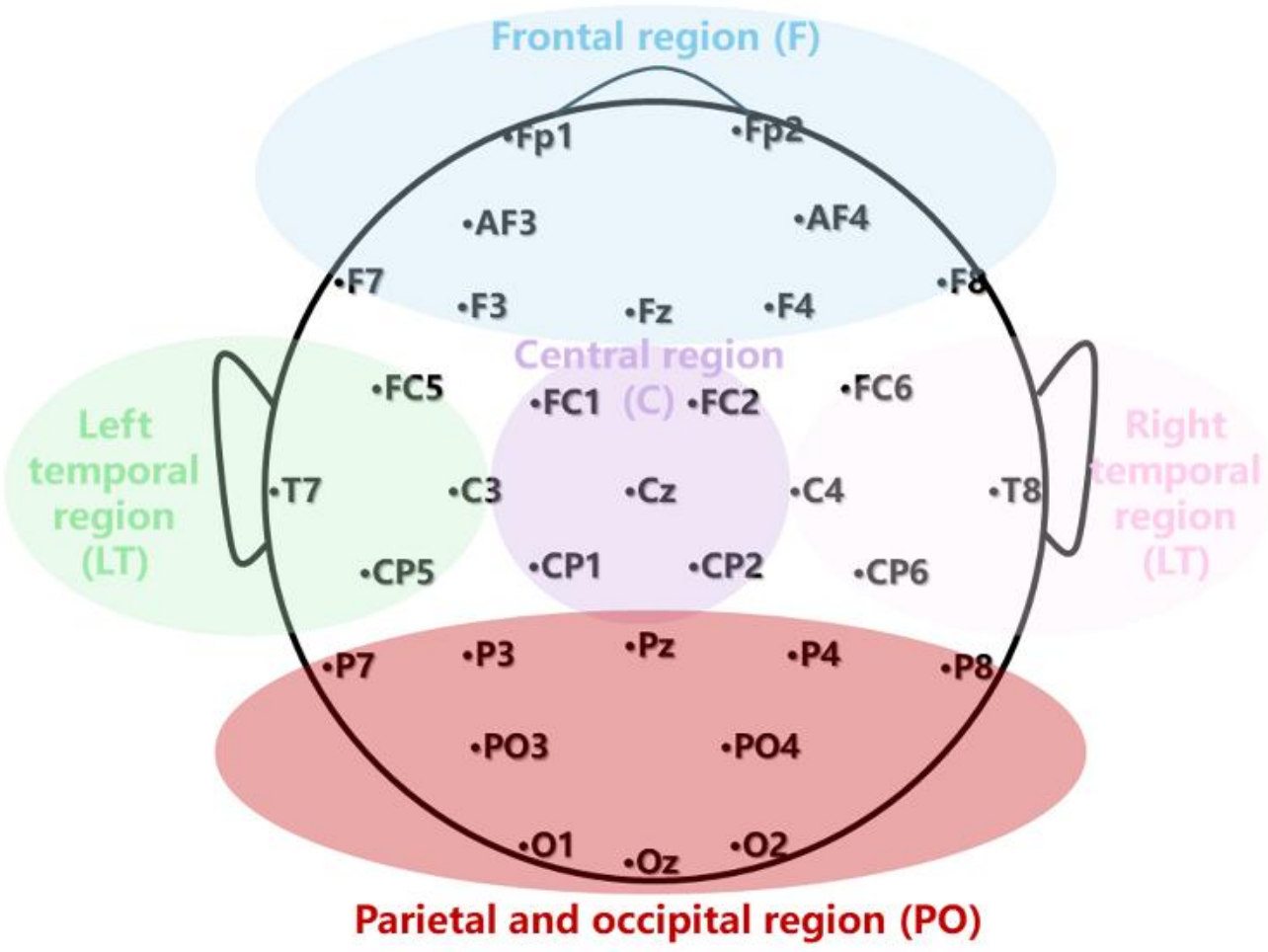

FIGURE S10. Schematic diagram of EEG electrode placement.

Table S1 Temporal Dominance of Sensations (TDS) metrics of dynamic sensory dominance during oral processing of grilled lamb skewers with varying grilling times.

Sample	Attribute	DR_max	T_max	D_90	D_sig	T_first	T_last	AAS
8 min	Chewy	0.2306	60.00	3.88	10.38	49.62	60.00	0.36
8 min	Fatty aroma	0.1239	0.00	0.00	0.00	0.00	0.00	0.00
8 min	Fatty retronasal odor	0.4303	37.55	18.58	39.66	15.16	54.82	1.28
8 min	Grilled aroma	0.0594	0.00	0.00	0.00	0.00	0.00	0.00
8 min	Grilled retronasal odor	0.4051	18.99	10.65	31.08	6.27	37.35	0.97
8 min	Grilled-lamb retronasal odor	0.2905	53.60	14.70	48.81	11.19	60.00	1.15
8 min	Juiciness	0.2322	0.00	1.53	8.46	0.00	8.46	0.30
8 min	Meaty aroma	0.1071	0.00	0.00	0.00	0.00	0.00	0.00
8 min	Meaty flavor	0.3025	0.00	2.12	7.63	0.00	7.63	0.36
8 min	Muttony flavor	0.1965	60.00	1.23	4.77	55.23	60.00	0.16
8 min	Salty	0.1972	45.12	16.60	28.58	27.46	56.04	0.57
8 min	Smoky retronasal odor	0.2171	19.19	16.55	32.57	5.65	38.22	0.71
8 min	Tender	0.2820	0.00	3.40	9.74	0.00	9.74	0.34
8 min	Umami	0.2692	44.70	17.15	33.67	23.95	57.62	0.61
10 min	Chewy	0.3403	60.00	1.78	8.82	51.18	60.00	0.34
10 min	Fatty aroma	0.0972	0.00	0.00	0.00	0.00	0.00	0.00
10 min	Fatty retronasal odor	0.3636	38.57	11.28	35.23	18.51	53.74	1.01
10 min	Grilled aroma	0.0703	0.00	0.00	0.00	0.00	0.00	0.00
10 min	Grilled retronasal odor	0.3350	18.46	9.38	28.27	6.46	34.73	0.90
10 min	Grilled-lamb retronasal odor	0.3625	18.82	13.44	38.07	21.93	60.00	1.08
10 min	Juiciness	0.3494	0.00	1.37	7.28	0.00	7.28	0.23
10 min	Meaty aroma	0.1849	0.00	0.93	3.45	0.00	3.45	0.12
10 min	Meaty flavor	0.2126	0.00	1.63	4.81	0.00	4.81	0.17
10 min	Muttony flavor	0.0360	0.00	0.00	0.00	0.00	0.00	0.00

Sample	Attribute	DR_max	T_max	D_90	D_sig	T_first	T_last	AAS
10 min	Salty	0.1417	27.35	9.55	18.71	18.83	37.55	0.11
10 min	Smoky retronasal odor	0.2569	16.66	14.17	27.66	5.80	33.46	0.84
10 min	Tender	0.2204	60.00	1.43	5.33	54.67	60.00	0.20
10 min	Umami	0.2389	46.89	12.10	28.76	29.78	58.54	0.64
12 min	Chewy	0.2242	60.00	2.88	13.06	0.00	60.00	0.36
12 min	Fatty aroma	0.0710	0.00	1.91	0.00	0.00	0.00	0.00
12 min	Fatty retronasal odor	0.4055	36.03	12.15	36.92	14.55	51.47	1.28
12 min	Grilled aroma	0.0685	0.00	0.00	0.00	0.00	0.00	0.00
12 min	Grilled retronasal odor	0.2951	21.55	13.13	30.52	8.85	39.37	0.93
12 min	Grilled-lamb retronasal odor	0.3804	52.23	12.61	30.64	29.36	60.00	1.11
12 min	Juiciness	0.0612	60.00	0.00	0.00	0.00	0.00	0.00
12 min	Meaty aroma	0.0970	0.00	0.00	0.00	0.00	0.00	0.00
12 min	Meaty flavor	0.2052	0.00	1.3	5.20	0.00	5.20	0.20
12 min	Muttony flavor	0.1315	60.00	0.73	1.44	58.56	60.00	0.08
12 min	Salty	0.1515	22.90	8.92	15.92	14.52	30.44	0.17
12 min	Smoky retronasal odor	0.2990	15.12	10.20	28.24	3.52	31.76	0.88
12 min	Tender	0.2140	0.00	1.12	5.38	0.00	5.38	0.22
12 min	Umami	0.2064	47.50	12.93	24.41	32.12	56.53	0.45

Note: a qualitative comparison was conducted on the curves, focusing on the Dr_max (maximum dominance ratio), T_max (time of D_max occurrence), D_90 (duration when Dr_max reaches 90%), D_sig (duration of significant dominance), T_first (time of first significant dominance occurrence), T_last (time of last significant dominance occurrence), and the area above the significance threshold curve (AAS).

Table S2 Temporal Dominance of Emotions (TDE) metrics of dynamic sensory dominance during oral processing of grilled lamb skewers with varying grilling times.

Sample	Attribute	DR_max	T_max	D_90	D_sig	T_first	T_last	AAS
8 min	Active	0.5022	43.40	10.92	30.03	7.24	51.88	1.54
8 min	Adventurous	0.1313	20.83	0.00	0.00	0.00	0.00	0.00
8 min	Calm	0.3724	60.00	7.79	17.71	42.29	60.00	0.75
8 min	Daring	0.3087	0.00	1.51	7.24	0.00	7.24	0.29
8 min	Disgusted	0.2344	60.00	1.55	5.07	54.93	60.00	0.25
8 min	Energetic	0.4101	0.00	1.86	9.89	0.00	9.89	0.42
8 min	Fun	0.1132	22.95	0.00	0.00	0.00	0.00	0.00
8 min	Happy	0.1923	35.47	13.89	22.92	23.77	46.69	0.69
8 min	Interested	0.1572	21.41	5.11	9.36	16.88	26.24	0.07
8 min	Nostalgic	0.1140	10.23	0.00	0.00	0.00	0.00	0.00
8 min	Pleasantly surprised	0.1063	37.27	0.00	0.00	0.00	0.00	0.00
8 min	Unpleasantly surprised	0.2235	60.00	1.24	5.48	54.52	60.00	0.26
8 min	Vigorous	0.1776	33.38	11.24	21.30	22.29	43.59	0.62
10 min	Active	0.3067	11.32	7.60	32.48	4.37	54.08	1.02
10 min	Adventurous	0.1815	20.19	11.14	15.97	12.91	28.88	0.54
10 min	Calm	0.4540	60.00	1.25	13.18	46.82	60.00	0.55
10 min	Daring	0.3286	0.00	1.10	7.39	0.00	7.39	0.28
10 min	Disgusted	0.2975	60.00	1.02	5.92	54.08	60.00	0.32
10 min	Energetic	0.2401	0.00	1.16	5.03	0.00	5.03	0.23
10 min	Fun	0.0782	12.31	0.00	0.00	0.00	0.00	0.00
10 min	Happy	0.2687	42.14	10.50	22.88	29.66	52.54	0.69
10 min	Interested	0.1796	19.27	6.05	15.95	12.41	28.36	0.48
10 min	Nostalgic	0.1530	0.00	2.37	4.11	0.00	4.11	0.19

Sample	Attribute	DR_max	T_max	D_90	D_sig	T_first	T_last	AAS
10 min	Pleasantly surprised	0.1351	34.81	0.00	0.00	30.03	37.19	0.06
10 min	Unpleasantly surprised	0.1443	60.00	0.00	0.34	59.66	60.00	0.16
10 min	Vigorous	0.2582	40.11	8.94	25.31	26.03	51.34	0.72
12 min	Active	0.4286	60.00	1.13	26.71	5.02	60.00	0.89
12 min	Adventurous	0.1708	16.86	3.53	14.84	11.78	26.62	0.43
12 min	Calm	0.2505	60.00	1.22	5.74	54.26	60.00	0.24
12 min	Daring	0.3028	0.00	1.82	7.41	0.00	7.41	0.29
12 min	Disgusted	0.1291	60.00	0.00	0.00	0.00	0.00	0.00
12 min	Energetic	0.1238	45.66	0.00	0.00	0.00	0.00	0.00
12 min	Fun	0.0689	20.12	0.00	0.00	0.00	0.00	0.00
12 min	Happy	0.2833	43.48	8.91	26.03	28.73	54.76	0.81
12 min	Interested	0.1863	18.60	6.25	16.35	11.33	27.68	0.54
12 min	Nostalgic	0.4035	0.00	1.91	11.33	0.00	11.33	0.47
12 min	Pleasantly surprised	0.1770	34.41	8.50	19.90	24.02	43.92	0.63
12 min	Unpleasantly surprised	0.1304	60.00	0.00	0.00	0.00	0.00	0.00
12 min	Vigorous	0.2782	39.21	9.69	27.51	24.02	51.53	0.85

Note: a qualitative comparison was conducted on the curves, focusing on the Dr_max (maximum dominance ratio), T_max (time of D_max occurrence), D_90 (duration when Dr_max reaches 90%), D_sig (duration of significant dominance), T_first (time of first significant dominance occurrence), T_last (time of last significant dominance occurrence), and the area above the significance threshold curve (AAS).

Table S3 Results of statistical data analysis describing the effects of mastication time and core temperature on bolus properties of grilled lamb skewers. F and p values are derived from linear mixed models with Grilling time and mastication time as fixed factor, and participant as random effect.

Bolus properties of grilled lamb skewers	Grilling time		Mastication time	
	F value	*P* value	*F* value	*P* value
**Bolus composition**				
Moisture content (% w/w)	4.89	**0.015**	119.54	**<0.001**
Fat content (% w/w)	349.90	**<0.001**	209.04	**<0.001**
Saliva uptake (% w/w)	5.85	**0.01**	66.32	**<0.001**
Sodium ion concentration	74.12	**<0.001**	274.95	**<0.001**
**Texture attributes**				
Hardness	106.94	**<0.001**	208.68	**<0.001**
Cohesiveness	9.31	**0.001**	138.15	**<0.001**
Gumminess	121.46	**<0.001**	5.11	**0.016**
**Microstructure characteristics**				
NumParticles	3.16	0.064	57.00	**<0.001**
MeanArea	9.71	0.001	39.72	**<0.001**
AreaStd	2.35	0.121	30.88	**<0.001**
AreaRatio	3.69	**0.043**	83.87	**<0.001**
MeanCircularity	5.01	**0.017**	63.90	**<0.001**

Table S4 Results of human saliva analysis.

Salivary physiological indicators	Gender	Resting state	Chewing state
pH	Male	7.58±0.06^{Ab}	7.72±0.09^{Aa}
	Female	7.49±0.03^{Ab}	7.67±0.05^{Aa}
MUC5B (mg/L)	Male	120.34±14.06^{Ab}	156.12±13.46^{Aa}
	Female	117.51±11.11^{Ab}	152.08±14.76^{Aa}
MUC7 (mg/L)	Male	89.93±9.01^{Ab}	113.58±13.76^{Aa}
	Female	85.77±3.01^{Ab}	103.28±13.25^{Aa}
Sodium ion concentration (mg/kg)	Male	109.62±5.24^{Bb}	145.47±16.45^{Aa}
	Female	109.36±2.09^{Bb}	144.24±8.97^{Aa}

Note: Different uppercase letters (A, B, etc.) indicate significant differences in salivary parameters between males and females under the same condition (resting or chewing state) ($P < 0.05$); different lowercase letters (a, b, etc.) indicate significant differences in salivary parameters between different conditions within the same gender ($P < 0.05$).

Table S5 Key simulated parameters of intranasal airflow dynamics and aroma-mucosa collision, including maximum/mean velocity field, maximum/mean collision intensity, and peak location coordinates (Z-axis), for grilled lamb skewers with different grilling times.

Key parameters		Grilled lamb skewers		
		8 min	10 min	12 min
Velocity Field	Max	0.473	1.332	2.280
	Mean	0.310	0.651	1.128
Collision Intensity	Max	0.550	1.000	1.005
	Mean	0.220	0.329	0.591
Peak Locations	Velocity Energy	Z = 22.5 mm	Z = 20.6 mm	Z = 13.2 mm
	Collision Energy	Z = 22.0 mm	Z = 22.0 mm	Z = 22.5 mm

Table S6. Sensitivity analysis of inlet velocity perturbations in nasal airflow simulation.

Velocity setting	Mean inlet velocity (m/s)	Mean airflow velocity	Mean collision intensity	Hotspot Z position (mm)	Hotspot X position (px)	Map correlation vs baseline
-10% inlet velocity	1.66	0.532	1.025	22.1	31	0.983
Baseline	1.84	0.591	1.128	22.5	32	1.000
+10% inlet velocity	2.02	0.650	1.236	22.9	33	0.978

Table S7 The identification result of aroma perceived from the retronasal cavity based on breath GC-IMS

Count	Compound	CAS #	Formula	MW	RI	Rt [sec]	Dt [a.u.]	8-10s	8-30s	8-60s	10-10s	10-30s	10-60s	12-10s	12-30s	12-60s
	Aldehydes															
1	Propanal	123-38-6	C3H6O	58.1	512.4	160.71	1.06	212.4±3.01aA	165.62±10.59aA	200.91±38.87aA	211.37±7.4aA	111.86±43.06bB	155.91±11.12abA	106.67±8.69aB	88.38±3.66aB	162.01±78.65aA
2	Isobutanal	78-84-2	C4H8O	72.1	540.9	173.99	1.09	435.5±81.18aA	237.23±9.66bA	129.75±37.27cA	236.89±3.45aB	234.93±4.82aA	114.56±13.27bA	262.28±21.04aB	212.34±5.79bB	129.2±30.84cA
3	2-Methyl-2-propenal	78-85-3	C4H6O	70.1	572.2	189.79	1.06	2287.82±146.2aA	787.53±89.46bB	1843.47±782.56abA	1924.99±9.6aB	1272.86±200.82bA	732.82±103.78cB	697.04±37.62aC	674.14±35.68aB	752.45±209.53aB
4	3-Methylbutanal	590-86-3	C5H10O	86.1	628.1	221.75	1.17	138.27±34.8bB	302.57±49.44aB	115.15±33.12bA	88.54±16.53bC	206.03±40.41aB	108.29±15.61bA	305.31±18.84bA	686.47±63.2aA	119.16±22.97cA
5	(E)-2-Butenal-D	123-73-9	C4H6O	70.1	665	239.12	1.2	114.97±23.2aA	104.88±15.57aA	89.73±24.4aA	102.01±5.06aA	97.72±11.8aA	63.37±2.32bA	112.7±5.66aA	99.82±1.09abA	76.4±16.95bA

Count	Compound	CAS #	Formula	MW	RI	Rt [sec]	Dt [a.u.]	8-10s	8-30s	8-60s	10-10s	10-30s	10-60s	12-10s	12-30s	12-60s
6	(E)-2-Butenal-M	123-73-9	C4H6O	70.1	5.2650.8	236.16	1.04	348.94±6.56aA	210.21±8.29bA	208.57±46.09bB	291.89±16.46aB	234.3±25.6aA	301.71±62.99aB	332.09±12.47bA	233.12±17.95bA	456.66±85.07aA
7	n-Pentanal	110-62-3	C5H10O	86.1	696.4	270.51	1.19	174.85±9.76aA	139.6±15.83aA	189.59±105.62aA	222.18±8.94aA	203.53±33.87aA	126.13±27.18bA	210.97±42.98aA	223.09±63.05aA	88.4±14.18bA
8	(E)-2-Pentenal	1576-87-0	C5H8O	84.1	725.3	302.89	1.09	399.9±42.9aB	320.86±273.19aA	314.48±124.82aA	200.18±14.64bC	307.15±31.69aA	209.02±62.65abA	862.16±114.82aA	569.83±160.9bA	152.46±13.31cA
9	Hexanal-M	66-25-1	C6H12O	100.2	795.7	398.25	1.28	228.46±20.34aA	161.71±9.17bA	132.48±10.98cA	159.56±8.52aB	176.67±12.02aA	144.01±23.68aA	150.89±13.03aB	182.39±12.34aA	183.66±58.26aA
10	Hexanal-D	66-25-1	C6H12O	100.2	798.5	400.17	1.32	36.03±2.74aA	41.58±2.29aA	41.11±10.28aA	38.18±1.52aA	41.82±3.92aA	43.6±2.29aA	39.32±2.99aA	37.57±0.69aA	147.64±184.91aA
	Alcohols															

Count	Compound	CAS #	Formula	MW	RI	Rt [sec]	Dt [a.u.]	8-10s	8-30s	8-60s	10-10s	10-30s	10-60s	12-10s	12-30s	12-60s
11	Ethanol-D	64-17-5	C2H6O	46.1	454.9	136.96	1.13	2830.15±529.95aA	2941.66±185.86aA	2473.69±173.12aA	2649.12±96.01aA	2494.51±54.18abB	2429.81±83.89bA	2883.05±40.9aA	2910.68±108.65aA	2323.02±268.62bA
12	Ethanol-M	64-17-5	C2H6O	46.1	455.9	137.32	1.04	3342.39±408.74aAB	3408.42±537.38aA	3791.08±90.41aB	3598.49±96.7aA	3636.53±236.39aA	3863.82±159.66aAB	3049.9±35.65bB	3072.94±40.33bA	4073.49±50.13aA
13	2-Methyl-2-propanol	75-65-0	C4H10O	74.1	524.3	166.11	1.14	243.95±45.53bB	555.42±100.04aA	245.23±38.78bA	230.07±21.57bB	351.98±26.6aB	268.08±57.66bA	510±24.56bA	556.91±11.83aA	294.9±11.25cA
14	1-Propanol-D	71-23-8	C3H8O	60.1	554.2	180.55	1.26	998.2±18.97aA	651±72.85bAB	614.22±133.54bA	1110.22±59.05aA	839.3±149.98bA	663.4±81.09bA	717.09±75.73aB	629.7±45.86aB	620.94±112.89aA
15	1-Propanol-M	71-23-8	C3H8O	60.1	555.6	181.21	1.11	381.9±71.83aB	121.14±2.49bA	104.62±38.34bA	530.66±45.17aA	220.15±87.11bA	116.18±31.41bA	252.46±16.14aC	213.83±21.95aA	125.84±49.12bA
16	2-Propanethiol-M	75-33-2	C3H8S	76.2	55	182.47	1.14	239.99±5.94aA	168.46±5.35bA	163.26±36.18bB	245.79±23.55aA	182.45±25.23bA	217.12±32.39abB	248.81±6.89abA	187.31±8.47bA	318.54±71.88aA

Count	Compound	CAS #	Formula	MW	RI	Rt [sec]	Dt [a.u.]	8-10s	8-30s	8-60s	10-10s	10-30s	10-60s	12-10s	12-30s	12-60s
17	2-Propanethiol-D	75-33-2	C3H8S	76.2	6.1563.9	185.45	1.41	92.52±21.95bB	195.21±62.72aB	83.15±56.44bA	64.88±25.65bB	106.24±23.51aC	60.52±5.13bA	707.07±7.06bA	1027.32±36.84aA	70.23±45.83cA
18	2-Butanol	78-92-2	C4H10O	74.1	585.4	196.88	1.15	114.97±23.2aA	104.88±15.57aA	89.73±24.4aA	102.01±5.06aA	97.72±11.8aA	63.37±2.32bA	112.7±5.66aA	99.82±1.09aA	76.4±16.95bA
19	n-butanol	71-36-3	C4H10O	74.1	669.2	248.6	1.18	282.94±4.35aA	234.44±6.49bA	195.87±6.67cB	249.4±4.53aB	268.28±43.48aA	258.08±45.08aAB	308.26±21.27aA	302.2±39.79aA	340.75±80.5aA
20	2-pentanol	6032-29-7	C5H12O	88.1	699	273.28	1.21	142.19±15.29aA	91.26±2.83bA	74.54±17.61bA	85.44±16.91aB	83.72±9.55aA	51.14±7.49bA	74.04±11.42aB	67±3.56aB	62.4±4.58aA
	Esters															
21	ethyl formate-D	109-94-4	C3H6O2	74.1	502.9	156.53	1.23	351.84±23.49abB	386.43±6.04aB	334.26±35.62bA	350.15±30.41aB	424.48±38.56aAB	357.38±44.1aA	434.84±7.01abA	442.26±12.65aA	379.11±49.31bA

Count	Compound	CAS#	Formula	MW	RI	Rt [sec]	Dt [a.u.]	8-10s	8-30s	8-60s	10-10s	10-30s	10-60s	12-10s	12-30s	12-60s
22	ethyl formate-M	109-94-4	C3H6O2	74.1	504.7	157.31	1.09	157.63±10.91bB	239.8±44.33aB	128.05±12.61bA	115.21±5.42aC	156.15±42.64aC	107.39±8.12aAB	345.3±10.47bA	654.93±10.96aA	100.65±16.09cB
23	acetic acid methyl ester-D	79-20-9	C3H6O2	74.1	526.2	166.99	1.19	220.64±9.2aA	199.36±15.33aA	257.71±56.14aA	204.25±9.89aB	162.84±19.43bB	220.13±27.53aA	152.82±2.66bC	162.58±8.41bB	223.44±25.03aA
24	acetic acid methyl ester-M	79-20-9	C3H6O2	74.1	528.7	168.17	1.03	404.5±17.91bA	372.92±6.63bAB	477.45±28.22aA	387.85±17.87bA	383.09±15.16bA	476.09±64.42aA	353.29±6.39bB	355.33±5.36bB	488.14±37.42aA
25	ethyl formate	109-94-4	C3H6O2	74.1	566.1	186.61	1.2	404.93±113.79bB	837.81±104.8aB	459.54±298.42abA	391.42±62.24bB	566.73±111.96aC	465.72±68.16abA	1177.38±57.42aA	1274.35±61.36aA	503.87±218.92bA
26	methyl propionate-M	554-12-1	C4H8O2	88.1	610.2	210.97	1.09	2370.31±74.88aA	1895.03±137.17aA	2230.68±596.59aA	2433.37±72.52aA	1029.37±823.68bA	1676.23±139.98abA	638.96±58.15aB	1081.12±168.2aA	1789.41±1038.91aA
27	methyl propionate-D	554-12-1	C4H8O2	88.1	61	211.56	1.33	1207.62±18.32aA	1336.47±69.56aA	1244.55±87.68aA	1244.99±4.75abA	1033.29±201.22bB	1300.28±54.26aA	980.92±35.87bB	1131.03±34.8abAB	1287.52±141.17aA

Count	Compound	CAS #	Formula	MW	RI	Rt [sec]	Dt [a.u.]	8-10s	8-30s	8-60s	10-10s	10-30s	10-60s	12-10s	12-30s	12-60s
28	3-Buten-1-ol, 3-methyl-	763-32-6	C5H10O	86.1	1.2719.2	295.7	1.16	102.4±19.64aAB	66.19±16.84aA	78.98±23.55aA	75.49±28.7abB	113.28±35.35aA	38.45±5.23bA	142.27±31.74aA	109.44±9.46aA	113.3±119.55aA
29	ethyl 2-methylpropanoate	97-62-1	C6H12O2	116.2	743.6	325.35	1.19	58.42±2.12bB	84.97±3.12aB	60.38±7.3bA	57.28±9.47bB	97.69±25.2aB	57.93±8.83bA	173.25±6.65bA	210.1±26.74aA	58.09±13.38cA
30	Ethyl butanoate	105-54-4	C6H12O2	116.2	803.6	410.26	1.21	58.17±4.54aA	53.63±9.62aA	58.22±2.01aA	51.89±5.37aA	55.42±3.77aA	52.89±6.74aA	51.52±3.89aA	60.02±2.09aA	54.98±9.71aA
	Ketones															
31	2-Propanone	67-64-1	C3H6O	58.1	489.8	150.9	1.11	6935.65±21.94aB	6897.44±343.55aA	6940.78±22.37aA	7054.2±17.63aA	7054.32±33.72aA	6991.81±251.29aA	6488.25±93.52bC	6221.3±133.08cB	7174.05±18.38aA
32	Acetone	67-64-1	C3H6O	58.1	466.7	141.54	1.09	1055.83±131.5aAB	1009.38±26.59abB	863.79±23.47bA	873.27±48.24aB	921.46±5.17aC	929.62±25.15aA	1135.14±131.8aA	1261.9±10.68aA	888.24±72.75bA

Count	Compound	CAS #	Formula	MW	RI	Rt [sec]	Dt [a.u.]	8-10s	8-30s	8-60s	10-10s	10-30s	10-60s	12-10s	12-30s	12-60s
33	2,3-butandione	431-03-8	$C_4H_6O_2$	86.1	570.7	189.02	1.16	409.05±30.27aA	129.44±14.62aB	418.74±306.31aA	277.67±29.41aB	125.09±10.91bB	73.36±5.57cA	223.52±25.16aB	232.2±19.24aA	78.74±24.4bA
34	2-Butanone-M	78-93-3	C_4H_8O	72.1	589	198.89	1.06	270.54±51.55aAB	268.24±98.17aA	189.52±132.05aA	174.5±93.56aB	373.17±202.8aA	124.44±14.28aA	364.8±20.96aA	356.99±43.22aA	151.72±37.35bA
35	2-Butanone-D	78-93-3	C_4H_8O	72.1	589.5	199.14	1.24	1350.66±59.25aA	911.92±75.98aA	1404.89±430.95aA	1003.55±100.05aB	999.12±191.47aA	734.77±20.29bB	995.31±10.45aB	993.65±27.73aA	790.68±47.1bB
36	1-penten-3-one	1629-58-9	C_5H_8O	84.1	653.2	237.74	1.08	258.61±22.59aB	305.91±56.05aA	322.35±105.88aA	294.06±7.1abA	332.32±52.82aA	259.98±18.97bA	139.46±14.59bC	196.24±38.6abB	212.51±29.28aA
37	3-Pentanone	96-22-0	$C_5H_{10}O$	86.1	689.2	263.01	1.11	776.98±58.25aA	505.85±62.05aB	719.3±318.43aA	684.77±96.65aAB	689.66±60.2aA	428.9±26.77bA	608.58±14.26bB	793.06±41.71aA	453.27±85.82cA
38	2-butanone 3-hydroxy-D	513-86-0	$C_4H_8O_2$	88.1	705.4	280.19	1.32	4170.37±1170.03aA	79.28±21.09bB	1849.01±2553.73bcA	357.66±155.84aB	145.61±37.21aA	149.55±97.18aA	100.59±15.6aB	72.23±9.81bB	70.07±7.83bA

Count	Compound	CAS#	Formula	MW	RI	Rt [sec]	Dt [a.u.]	8-10s	8-30s	8-60s	10-10s	10-30s	10-60s	12-10s	12-30s	12-60s
39	2-butanone 3-hydroxy-M	513-86-0	C4H8O2	88.1	705.6	280.38	1.08	1937.01±41.35aA	392.13±102.37bA	1187.95±953.43bcA	914.4±195.25aB	331.61±66.89bA	556.76±228.87abA	292.91±37.07aC	263.21±50.41aA	326.85±49.99aA
40	2,3-pentandione	600-14-6	C5H8O2	100.1	706	280.8	1.22	726.68±182.36aA	126.9±30.5bA	388.52±383.8abA	166.73±20.45aB	107.94±16.48bAB	103.7±16.52bA	77.52±5.96bB	78.32±7.11bB	107.34±12.69aA
41	2-hexanone	591-78-6	C6H12O	100.2	761.4	348.71	1.22	115.45±57.3aB	156.66±52.74aA	113.84±102.06aA	143.46±107.32abB	358.78±173.59aA	59.06±15.47bA	409.71±93.29aA	366.7±169.83aA	61.27±8.87bA
42	2-methyltetrahydrofuran-3-one	3188-00-9	C5H8O2	100.1	773.9	366.23	1.1	159.35±14.82aB	147.07±8.96aB	157.01±53.54aA	111.31±23.14bB	146.04±2.71aB	120.12±9.91abA	224.97±39.55abA	313.15±68.37aA	142.42±89.51bA
	Pyrazines															
43	pyrazine	290-37-9	C4H4N2	80.1	708.7	283.84	1.04	75.37±28.54bB	367.54±33.61aA	218.25±144.91abB	316.58±45.51bA	348.41±20.32abA	419.61±41.21aA	279.79±3.81bA	322.71±12.36bA	455.73±41.47aA

Count	Compound	CAS#	Formula	MW	RI	Rt [sec]	Dt [a.u.]	8-10s	8-30s	8-60s	10-10s	10-30s	10-60s	12-10s	12-30s	12-60s
44	2-methylpyrazine	109-08-0	C5H6N2	94.1	792.6	393.64	1.1	164.39±92.7aA	90.73±33.73aB	82.33±15.48aA	103.17±29.54bA	162.85±23.04aA	57.42±12.78cA	164.58±6.27aA	86.83±37.93bB	59.78±19.5bA
45	2-ethylpyrazine	13925-00-3	C6H8N2	108.1	885.7	559.62	1.15	298.94±128.46aB	295.28±193.75aA	175.16±10.37aA	294.28±79.64bB	558.65±26.37aA	149.74±27.8cA	700.07±49.99aA	341.13±114.56bA	139.68±23.19cA
46	2,3-diethylpyrazine	15707-24-1	C8H12N2	136.2	1054	1057.26	1.69	3141.94±313.26bB	4400.18±324.77aA	3814.63±474.85abA	3399.47±527.54bB	5882.46±1168.85aA	4250.74±161.74abA	4663.35±56.87abA	5619.99±629.67aA	4213.9±976.59bA
	Other															
47	Dimethyl disulfide	624-92-0	C2H6S2	94.2	712.9	288.58	1.12	152.79±49.22aB	164.06±61.69aB	156.44±82.15aA	167.3±70.07bB	528.77±162.24aA	86.99±70.03bA	413.46±70.94aA	283.47±131.05aAB	89.18±41.45bA
48	2-Methylbutanoic acid	116-53-0	C5H10O2	102.1	841	472.67	1.48	395.82±75.07bB	818.43±98.96aA	386.47±107.16bA	349.39±18.66bB	555.83±117.45aB	415.18±53.18abA	651.06±44.97aA	657.39±89.23aAB	398.9±120.68bA

Note: Different uppercase letters (A, B, etc.) indicated significant differences among the boluses of 8-min, 10-min and 12-min lamb skewers at the same chewing time (10, 30, and 60 s) ($P < 0.05$). Different lowercase letters (a, b, etc.) indicated significant differences among the boluses at different chewing times (10, 30, and 60 s) for the same grilled lamb skewers ($P < 0.05$). "DT", the drift time of IMS; "RI", retention index; "MW", molecular weight; "Rt", retention time.

.

Table S8. Fisher's z-test comparison of FAA-midline theta coupling under CH-NC and CH-C conditions.

Test	Statistic	Value
CH-NC FAA-theta coupling	r; Fisher's z; 95% CI for r	0.36; 0.377; 0.19 to 0.51
CH-C FAA-theta coupling	r; Fisher's z; 95% CI for r	0.10; 0.100; -0.08 to 0.27
CH-NC vs CH-C	Delta r; Delta z; Z; p	0.26; 0.277; 2.12; 0.034
Effective sample size	n_eff per condition	120; raw window count = 360

Table S9 Model performance under stratified airflow velocity conditions

**Airflow velocity grouping**	**Simulated total number of molecules N (sequence × time steps)**	**R^2**	**RMSE**	**MAE**
Low flow rate	1000	0.912	0.348	0.271
Medium flow rate	1000	0.924	0.336	0.262
High flow rate	1000	0.905	0.356	0.278

Note: The maximum intergroup fluctuations were $\Delta R^2 = 0.019$, ΔRMSE = 0.020, ΔMAE = 0.016.

Table S10 Model performance under stratified MUC5B levels

MUC5B grouping	Simulated total number of molecules N (sequence × time steps)	R^2	RMSE	MAE
Low concentration MUC5B	1000	0.918	0.341	0.266
Medium concentration MUC5B	1000	0.927	0.333	0.258
High concentration MUC5B	1000	0.909	0.352	0.274

Note: The maximum intergroup fluctuations were $\Delta R^2 = 0.018$, ΔRMSE = 0.019, ΔMAE = 0.016.

Table S11 List of sensory attributes and definitions for TDS evaluation of grilled lamb skewers

Attributes	Definition
Fatty aroma	The aroma of oil produced by the volatilization of lamb fat during grilling.
Meaty aroma	The typical cooked meat aroma derived from protein degradation and the Maillard reaction.
Grilled aroma	The aroma is produced by high-temperature charcoal grilling
Grilled-lamb retronasal odor	The distinctive complex aroma of roasted lamb perceived through the retronasal pathway during chewing
Fatty retronasal odor	The aroma of fat perceived retronasally during chewing, characteristic of lamb fat.
Grilled retronasal odor	The grilled aroma perceived retronasally during chewing from high temperatures.
Smoky retronasal odor	The aroma of meat smoked over charcoal, perceived through the retronasal pathway while chewing.
Salty	The basic taste sensation produced by sodium ions from meat stimulating the taste buds.
Umami	The rich and savory taste brought by amino acids such as glutamate in meat.
Muttony flavor	The distinctive flavor of lamb, associated with branched-chain fatty acids, may be perceived as unpleasant.
Meaty flavor	The overall flavor of the cooked meat
Tender	The force required for teeth penetrate the meat reflects its tenderness.
Juiciness	The sensation of juices being released from the meat as you chew
Chewy	Reflecting the ability of meat to withstand repeated chewing.

Table S12 List of sensory attributes and definitions for TDE evaluation of grilled lamb skewers

Attributes	Definition
Active	Rich and varied flavors and textures that continuously engage and stimulate the senses.
Fun	The simple pleasure of chewing derived from the meat's texture.
Disgusted	Unpleasant negative experiences caused by the distinct smell and taste of lamb, either from excessive dehydration or charring.
Daring	Pushing the psychological boundaries of one's acceptance of lamb "doneness."
Adventurous	Systematically exploring the flavor profile influenced by time variables.
Calm	Familiarity with the current flavor and texture, bringing a sense of comfort and relaxation from easy acceptance.
Energetic	The immediate energy and freshness from the burst of meat juices.
Happy	Instant gratification when reaching the perfect balance point of personal preference.
Pleasantly surprised	The actual experience exceeds expectations for a specific roasting time, resulting in a delicious encounter.
Interested	Cognitive curiosity about the linear differences caused by time.
Unpleasantly surprised	A specific roasting time fails to deliver its expected advantages or presents flaws.
Nostalgic	Primitive barbecue memories evoked by specific textures or aromas.
Vigorous	Intense, bold satisfaction from the firm texture and concentrated flavors.

Table S13. Key parameters and boundary conditions used in CFD-particle transport simulation.

Parameter	Setting, unit, and source
Nasal geometry	Average nasal model reconstructed from 130 NasalSeg CT scans by Marching Cubes; unit: mesh; source: NasalSeg dataset [41].
Inlet airflow u_{in}(t)	Measured mastication waveform; unit: m/s; peak velocity calibrated within 10%; source: HKH-11Q airflow sensor.
Outlet/wall boundary	Pressure outlet downstream and no-slip nasal wall; olfactory cleft set as target collision surface; unit: Pa / dimensionless; source: standard nasal CFD setup.
Air properties	rho = 1.225 kg/m3 and mu = 1.8 x 10^-5 Pa*s; source: Model 3 setting.
Particle tracking	Np = 1200 particles; 200 Hz coupling; time-step factor = 0.95; source: simulation setting.
Collision intensity	Wall/near-wall particle-contact density weighted by local transport intensity and normalized to 0-1; unit: a.u.; source: Figure 3 metric.
Particle source flux	Scaled by bolus exposed area and GC-IMS VOC abundance at the matched chewing time; unit: a.u.; source: Model 2-Model 3 coupling.
